# An Efficient Synthesis of Oxygen-Bridged Spirooxindoles via Microwave-Promoted Multicomponent Reaction

**DOI:** 10.3390/molecules28083508

**Published:** 2023-04-16

**Authors:** Yaojing Shi, Hua Zhao, Yufen Zhao

**Affiliations:** Institute of Drug Discovery Technology, Qian Xuesen Collaborative Research Center of Astrochemistry and Space Life Sciences, Ningbo University, Ningbo 315211, China

**Keywords:** green chemistry, microwave, multicomponent reaction, [3+2] cycloaddition, oxygen-bridged spirooxindoles

## Abstract

A microwave-promoted multicomponent reaction of isatins, α-amino acids and 1,4-dihydro-1,4-epoxynaphthalene is achieved under environmentally friendly conditions, delivering oxygen-bridged spirooxindoles within 15 min in good to excellent yields. The attractive features of the 1,3-dipolar cycloaddition are the compatibility of various primary amino acids and the high efficiency of the short reaction time. Moreover, the scale-up reaction and synthetic transformations of spiropyrrolidine oxindole further demonstrate its synthetic utility. This work provides powerful means to expand the structural diversity of spirooxindole as a promising scaffold for novel drug discovery.

## 1. Introduction

Green chemistry is essential to contribute to sustainability, it minimizes the energy requirements, reagent consumption, as well as the generation of wastes [1,2,3]. Microwave technology is in line with the category of green chemistry, since the first reports in the 1980s, microwave technology has proven to be a valuable tool for synthetic organic chemistry [4,5,6,7,8]. Reactions can be completed in minutes usually, and both product yields and selectivity can often be enhanced over conventional approaches. Compared with traditional thermal conditions (oil bath or a heating block), the distinctive features of microwave-assisted reactions include superior reaction efficiency, environmentally benign reaction condition, better control of the reaction process, and its capability to rapidly screen a wide range of experimental parameters. Based on the above characteristics, microwave technology provides a more convenient and ingenious alternative for green synthesis of high-value chemicals.

Multicomponent reactions (MCRs) represent one of the most powerful reactions leading to structurally diverse molecules, which allow the creation of several chemical bonds from simple starting material in one pot [9,10,11,12,13]. The remarkable advantages of multicomponent reactions include high bond-forming efficiency, operational simplicity and superior atom economy, making them convenient over the stepwise methods. Among the various *N*-heterocycles, spirooxindoles are featured widely in a variety of pharmacologically active substances [14,15], which is of great interest to synthetic chemists. [16,17,18,19,20,21,22,23,24]. As one of the most attractive subtypes, spiropyrrolidine oxindoles often exhibit intriguing biological activities [25]. Multicomponent reactions provide key opportunities for the construction of spiropyrrolidine oxindoles, and significant efforts have been made toward the designing of novel and practical strategies to access this important class of compounds [26,27,28,29,30,31].

**Scheme 1:**
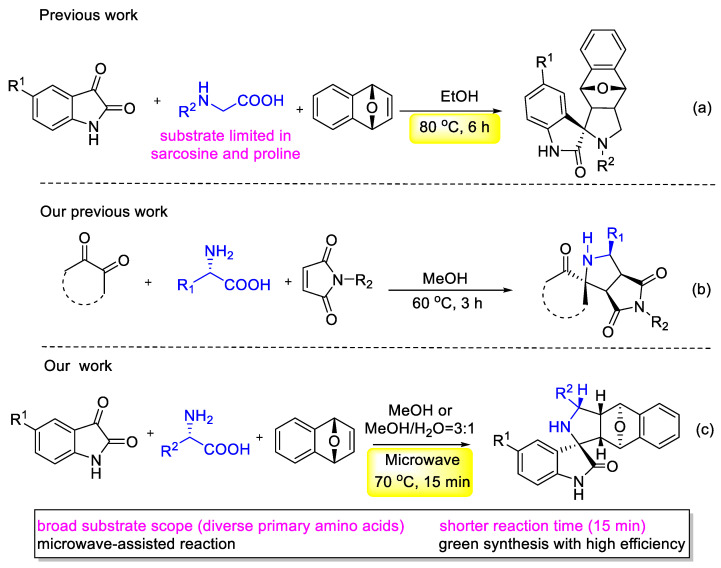
(**a**) Previous work of 1,3-dipolar cycloaddition of isatins, α-amino acids and oxabicyclic alkenes, (**b**) Our previous work of 1,3-dipolar cycloaddition, and (**c**) Microwave-promoted multicomponent reaction with diverse primary amino acids as reaction partner (This Work).

We notice that oxabicyclic alkenes show potentially interesting reactivities owing to the inherent strain, and they have been widely employed in both ring-retentive and ring-opening catalytic transformations, including dimerization [32], cycloaddition [33,34,35], ring-opening/rearrangement reaction [36], hydrofunctionalization [37,38,39,40], and C−H activation [41,42]. Though great success has been achieved in traditional 1,3-dipolar cycloaddition of azomethine ylides prepared from isatins and α-amino acids with various electron-deficient alkenes as dienophiles, the strained oxabicyclic alkenes are scarcely involved. To the best of our knowledge, there is only an elegant work disclosed by Parthasarathy and co-workers (Scheme 1a). They explored 1,3-dipolar cycloaddition of azomethine ylides with heterobicyclic alkenes at 80 °C for 6 h [43]. Despite considerable progress, it cannot be ignored that it remains a few challenges in the current method. One is the limited amino acids substrate scope, as the reported work focuses on sarcosine and proline. Moreover, the required reaction time often lasts for hours. Therefore, the synthesis of oxygen-bridged spirooxindoles in several minutes with diverse primary amino acids as substrates is quite challenging and not established. With these problems in mind, and in continuation of our interest in the 1,3-dipolar cycloaddition (Scheme 1b) [44], we wish to offer a microwave-promoted multicomponent reaction for the synthesis of oxygen-bridged spirooxindoles in a very short time with diverse primary amino acids as reaction partners (Scheme 1c).

## 2. Results and Discussion

We commenced our investigation with isatin **1a**, proline **2a** and 1,4-dihydro-1,4-epoxynaphthalene **3a** as model substrates. To our delight, microwave irradiation can promote the desired annulation reaction under catalyst- or additive-free conditions. Treatment of **1a, 2a** and **3a** in THF at 70 °C for 15 min, the desired corresponding [3+2] annulation product **4a** was obtained as a single diastereomer in 40% yield (Table 1, entry 1). Encouraged by the initial result, we then focused on solvent screening; typical polar and nonpolar solvents were tested for the reaction. Generally, the results revealed that the solvents have a great influence on the reaction outcome, and alcohols are better than other solvents (Table 1, entries 2–8). Notably, MeOH gave the optimal results (73% yield, Table 1, entry 5). Further extensive studies regarding the temperature were conducted; however, improving or lowering the temperature was not beneficial to the reaction outcome (entries 9–11). A further increase in the reaction time (20, 25 or 30 min) did not significantly improve the reaction yield (entries 12–14), and a shorter time (10 min) indicated a negative effect on the result of the reaction (entry 15). Finally, the yield could be improved to 83% by switching the equivalent of reactants (entries 16–17).

**Scheme 2:**
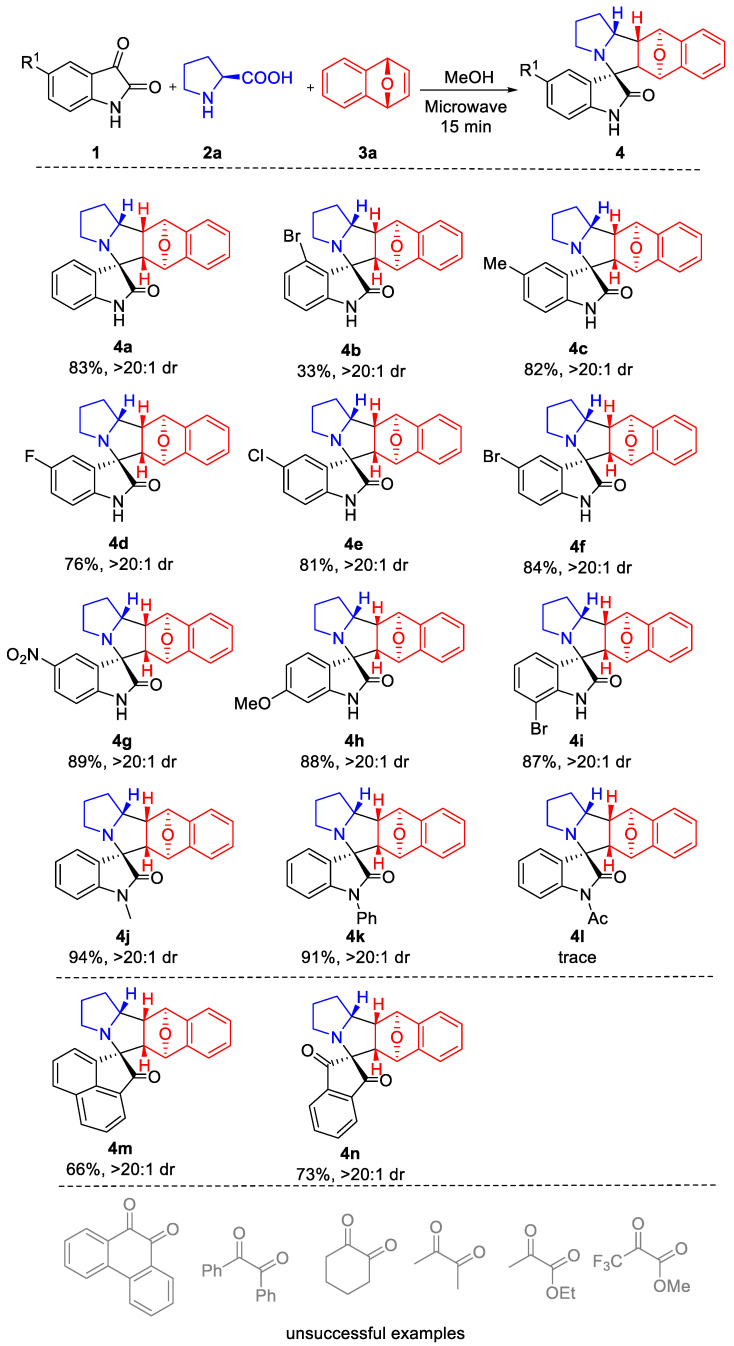
Reaction scope with respect to isatins and α-dicarbonyl compounds. Reagents and conditions: **1** (0.2 mmol), **2a** (0.2 mmol), **3a** (0.3 mmol), MeOH (2 mL), 70 °C, 15 min under air in a microwave reactor.

With the optimal conditions in hand, we explored the substrates’ scope of isatins, and the results were summarized in Scheme 2. Generally, isatins with various substitutions were suitable to the reaction condition. When 4-bromoisatin was subjected to the 1,3-dipole cycloaddition, product **4b** was obtained in 33% yield. We speculated that the lower reaction activity was attributed to a large steric hindrance group nearby the reactive center. Isatins equipped with electron-donating (methyl) or electron-withdrawing group (fluoro, chloride, bromide, nitro) at the C5 position of the benzene ring were well-tolerated for the cycloaddition, affording the desired adducts in good to excellent yields (**4c**–**4g**, 72–89% yields). Meanwhile, 6-methoxy-isatin participated in the three-component reaction smoothly, and **4h** was separated in 88% yield. When the substitution of bromide was far away from the active center, there was no obvious influence on the reaction result (87% yield of **4i**). Next, isatins with the *N*-protection group were explored, and the outcome showed that alkyl and phenyl groups were friendly to this reaction, giving **4j** and **4k** in 94% and 91% yields, respectively. However, N-acetyl protected isatin furnished only trace products. Furthermore, other diketones and their analogs such as acenaphthequinone and ninhydrin were also found to be effective, as demonstrated in the successful installation of **4m** and **4n**. However, other diketones including 9,10-phenanthrenequinone, 1,2-diphenyl ethanedione, cyclohexanedione, acetylacetone, ethyl pyruvate and ethyl 3,3,3- trifluoropyruvate failed to undergo this reaction under standard conditions.

**Scheme 3:**
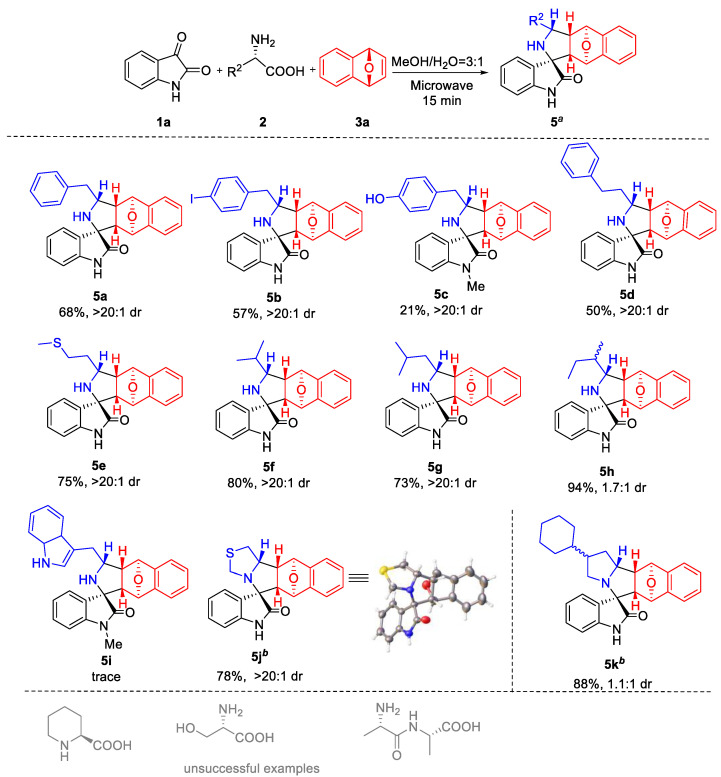
Reaction scope with respect to amino acids *^a^*. *^a^* Reagents and conditions: **1a** (0.20 mmol), **2** (0.2 mmol), **3a** (0.3 mmol), MeOH/H_2_O = 3:1 (2 mL), 70 °C, 15 min under air in a microwave reactor. *^b^* MeOH (2 mL) was used as solvent.

Next, to examine the feasibility of this reaction, we examined the variation of the amino acid component toward the formation of oxygen-bridged spirooxindoles (Scheme 3). Initially, the natural L-alanine was found to be unfruitful for the optimal condition, and no desired product could be isolated from the reaction mixture. We speculated that the side reactions of azomethine ylides from isatin and L-alanine are largely preferred over [3+2] cycloadditions with a dipolarophile, which makes this reaction particularly challenging. Gratefully, we found that the L-phenylalanine proceeded smoothly in methanol−water medium (MeOH:H_2_O = 3:1), but changing the solvent still had no positive effect on alanine. Under similar conditions, the present cycloaddition reaction was also successfully extended to other primary α-amino acids with side chains, thus greatly expanding the types of spirooxindoles accessible using this method. 4-Iodo-L-phenylalanine and L-tyrosine led to the **5b** and **5c** in 57% and 21% yields, respectively. It is worth noting that N-methyl protection is necessary for **5c**, otherwise, no expected product was observed in the reaction system. As a common intermediate in about 20 new antihypertensive drugs in the world, L-homophenylalanine showed quite promising reactivities, leading to the desired product **5d** in moderate yield. The three-component reaction of isatin with L-methionine, L-lysine and L-leucine with **3a** resulted in the expected products in 73–80% yields as single isomers. It was found that the L-isoleucine afforded the spirooxindoles **5h** as chromatographically separable diastereomers (1.7:1 dr). The use of tryptophan as a reaction partner proved to have low efficiency, delivering the corresponding product **5i** in only trace yield. It was hypothesized that in the presence of unprotected amino acid residues, the side reactions were preferred over the expected cyclization reaction. Next, L-thiproline was tested, and **5j** was obtained with a 78% yield. The structure and relative stereochemistry of **5j** was unambiguously established by Single-crystal X-ray analysis (CCDC: 2243483). When *trans*-4-cyclohexyl-L-proline was selected for the cycloaddition, the two diastereomers of **5k** were isolated in almost the same yield (46% vs. 42%). In contrast, the utilization of L-piperidine-2-carboxylic acid, L-serine and peptide L-Ala-L-Ala-OH proved unsuccessful for the reaction conditions.

**Scheme 4:**
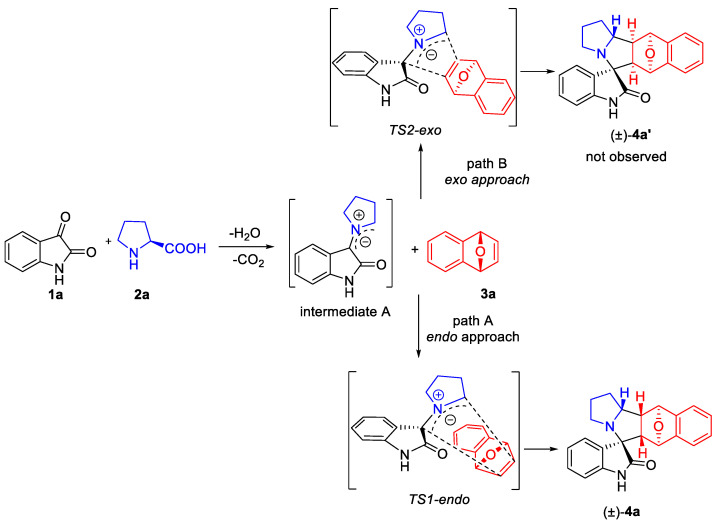
Proposed reaction mechanisms.

Based on the experimental results and the reported literature [45], possible mechanisms for cycloaddition were proposed as shown in Scheme 4. First, the condensation of isatin **1a** with proline **2a**, followed by decarboxylation furnishes ylide intermediate **A**, which reacts as a 1,3-dipole with **3a** through two possible pathways A and B. Path A leads to the formation of the thermodynamically more stable *endo*-cycloadduct **4a**, whereas affording the *exo*-cycloadduct **4a’** through path B is probably disfavored.

**Scheme 5:**
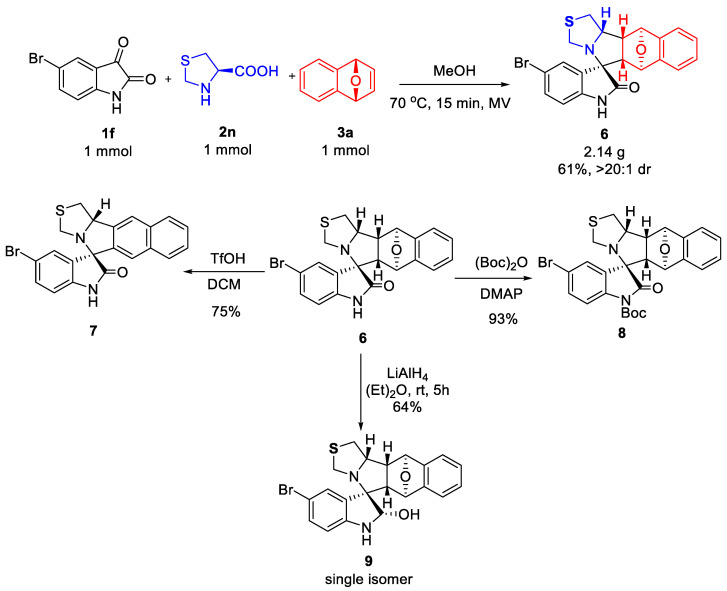
Gram scale experiment and derivatization of compound **6**.

To explore the synthetic utility of this established protocol, we investigated the one-pot three-component [3+2] cycloaddition cascade on a gram scale, affording the corresponding **6** in synthetically useful yield (61%) with excellent diastereoselectivity (Sheme 5). To further illustrate the synthetic values of this three-component reaction, the derivatizations of the gram reaction product were carried out (Scheme 5). First, in the presence of TfOH, compound **6** was successfully transformed into product **7** by deoxyaromatization. Then, **6** was treated with (Boc)_2_O/DMAP, affording *N*-Boc substituted spirooxindole **8** in good yield. Next, by reduction of **6** with LiAlH_4_ in diethyl ether at ambient temperature, 2-hydroxyindoline **9** was obtained as a single diastereomer in 64% yield. These representative examples highlight the advantages and potential application of this method.

## 3. Materials and Methods

### 3.1. General Information

All starting materials were purchased from commercial suppliers and used without further purification unless otherwise stated. Thin-layer chromatography (TLC) was conducted with 0.25 mm Tsingdao silica gel plates (60F-254) and visualized by exposure to UV light (254 nm). Flash column chromatography was performed on Tsingdao silica gel (200–300 mesh) and neutral/basic aluminum oxide (200–300 mesh). ^1^H NMR spectra were recorded with Bruker spectrometers (400 or 500 MHz) and reported relative to deuterated solvent signals or tetramethylsilane internal standard signals (see Supplementary Materials). Data for ^1^H NMR spectra were reported as follows: chemical shift (δ/ppm), multiplicity (s = singlet, d = doublet, t = triplet, q = quartet, m = multiplet, br = broad), coupling constant (J/Hz) and integration. ^13^C NMR spectra were recorded with Bruker spectrometers (125 MHz). Data for ^13^C NMR spectra were reported in terms of chemical shift. ^19^F NMR spectra were recorded with Bruker spectrometers (470 MHz). High-resolution mass spectrometry (HRMS) was conducted with a Bruker Apex IV RTMS. All microwave reactions were conducted in sealed glass vials with a microwave reactor, Discover SP from CEM Corp.

### 3.2. General Procedure for the Microwave Assisted 1,3-dipolar Cycloaddition of Azomethine Ylides Prepared from Isatins and α-Amino Acids with 1,4-dihydro-1,4-epoxynaphthalene

A glass vial was charged with Isatins **1** (0.20 mmol, 1.0 equiv), α-Amino Acids **2** (0.2 mmol, 1.0 equiv), 1,4-dihydro-1,4-epoxynaphthalene **3** (0.3 mmol, 1.5 equiv) and 2 mL of solvent (MeOH or MeOH/H_2_O = 3:1). The resulting mixture was placed in a monowave 200 microwave synthesis reactor and stirred at 70 °C for 15 min under air. After completion of the reaction as monitored by TLC, the resulting crude product was concentrated under reduced pressure, and the resulting crude product was purified by column chromatography to provide the desired product.

#### 3.2.1. 1′,2′,3′,5a’,6′,11′,11a’,11b’-Octahydrospiro[indoline-3,5′-[6,11]epoxybenzo[f]pyrrolo [2,1-a]isoindol]-2-one (**4a**)

Eluent = petroleum ether/EtOAc (1:1). white solid (57 mg, 83%). mp: 172−175 °C. ^1^H NMR (500 MHz, CDCl_3_) δ 8.52 (s, 1H), 7.54 (d, *J* = 7.5 Hz, 1H), 7.29 (td, *J* = 7.5, 1.0 Hz, 1H), 7.24 (d, *J* = 7.0 Hz, 1H), 7.16–6.99 (m, 4H), 6.91 (d, *J* = 8.0 Hz, 1H), 5.43 (s, 1H), 5.12 (s, 1H), 4.23–4.19 (m, 1H), 2.89 (d, *J* = 8.0 Hz, 1H), 2.82 (dt, *J* = 9.0, 7.0 Hz, 1H), 2.75 (t, *J* = 8.0 Hz, 1H), 2.48 (td, *J* = 9.0, 3.5 Hz, 1H), 2.01–1.90 (m, 3H), 1.88–1.81 (m, 1H). ^13^C NMR (125 MHz, CDCl_3_) δ 182.3, 146.9, 145.3, 141.9, 129.2, 128.4, 127.2, 126.9, 126.6, 122.3, 119.6, 119.0, 110.2, 80.3, 80.1, 71.3, 65.6, 58.4, 48.7, 46.2, 26.8, 25.5. HRMS (ESI-TOF): *m/z* calcd. for C_22_H_21_N_2_O_2_: 345.1603 [M+H]^+^; found: 345.1600.

#### 3.2.2. 4-Bromo-1′,2′,3′,5a’,6′,11′,11a’,11b’-octahydrospiro[indoline-3,5′-[6,11]epoxybenzo[f]pyrrolo [2,1-a]isoindol]-2-one (**4b**)

Eluent = petroleum ether/EtOAc (5:1). white solid (28 mg, 33%). mp: 248–250 °C. ^1^H NMR (400 MHz, DMSO-*d*_6_) δ 7.40 (d, *J* = 7.2 Hz, 1H), 7.31–7.24 (m, 3H), 7.22–7.13 (m, 2H), 6.90 (d, *J* = 7.2 Hz, 1H), 5.45 (s, 1H), 5.32 (s, 1H), 3.79–3.69 (m, 1H), 2.73 (t, *J* = 8.8 Hz, 1H), 2.64–2.61 (m, 1H), 2.48 (d, *J* = 7.2 Hz, 1H), 2.40–2.34 (m, 1H), 2.09–1.96 (m, 3H), 1.85–1.77 (m, 1H). ^13^C NMR (125 MHz, DMSO-*d*_6_) δ 181.9, 147.1, 145.3, 144.7, 131.1, 128.0, 127.6, 127.0, 126.9, 120.7, 120.4, 119.2, 109.7, 83.4, 80.1, 78.1, 71.9, 61.7, 49.9, 49.1, 32.6, 28.4. HRMS (ESI-TOF): *m/z* calcd. for C_22_H_20_BrN_2_O_2_: 423.0708 [M+H]^+^; found: 423.0706.

#### 3.2.3. 5-Methyl-1′,2′,3′,5a’,6′,11′,11a’,11b’-octahydrospiro[indoline-3,5′-[6,11]epoxybenzo[f]pyrrolo [2,1-a]isoindol]-2-one (**4c**)

Eluent = petroleum ether/EtOAc (1:1). white solid (59 mg, 82%). mp: 183−185 °C. ^1^H NMR (500 MHz, CDCl_3_) δ 8.21 (s, 1H), 7.38 (s, 1H), 7.27 (d, *J* = 7.5 Hz, 1H), 7.20–7.07 (m, 4H), 6.82 (d, *J* = 8.0 Hz, 1H), 5.47 (s, 1H), 5.22 (s, 1H), 4.26–4.22 (m, 1H), 2.89 (d, *J* = 7.5 Hz, 1H), 2.86 (t, *J* = 8.0 Hz, 1H), 2.74 (t, *J* = 8.0 Hz, 1H), 2.49 (td, *J* = 9.0, 4.0 Hz, 1H), 2.39 (s, 3H), 2.04–1.87 (m, 4H). ^13^C NMR (125 MHz, CDCl_3_) δ 181.6, 146.9, 145.3, 139.4, 131.8, 129.5, 128.9, 127.1, 126.9, 126.6, 119.6, 119.0, 109.8, 80.4, 80.0, 70.8, 65.4, 58.3, 48.4, 45.8, 26.8, 25.2, 21.3. HRMS (ESI-TOF): *m/z* calcd. for C_23_H_23_N_2_O_2_: 359.1760 [M+H]^+^; found: 359.1753. 

#### 3.2.4. 5-Fluoro-1′,2′,3′,5a’,6′,11′,11a’,11b’-octahydrospiro[indoline-3,5′-[6,11]epoxybenzo[f]pyrrolo [2,1-a]isoindol]-2-one (**4d**)

Eluent = MeOH/DCM (1:80). white solid (55 mg, 76%). mp: 156–158 °C. ^1^H NMR (500 MHz, CDCl_3_) δ 8.30 (s, 1H), 7.33 (dd, *J* = 8.5, 3.0 Hz, 1H), 7.25 (d, *J* = 7.5 Hz, 1H), 7.14 (td, *J* = 7.0, 2.0 Hz, 1H), 7.12–7.06 (m, 2H), 7.01 (td, *J* = 8.5, 2.5 Hz, 1H), 6.84 (dd, *J* = 8.5, 4.5 Hz, 1H), 5.42 (s, 1H), 5.08 (s, 1H), 4.19–4.15 (m, 1H), 2.92 (d, *J* = 8.0 Hz, 1H), 2.76–2.72 (m, 2H), 2.50 (td, *J* = 9.0, 4.0 Hz, 1H), 2.06–1.82 (m, 4H). ^13^C NMR (125 MHz, CDCl_3_) δ 182.1, 158.9 (*J* = 240.0 Hz), 146.9, 145.0, 137.7, 128.9, 127.0, 126.6, 119.7, 119.0, 116.2 (*J* = 25.0 Hz), 115.7 (*J* = 23.7 Hz), 110.5 (*J* = 8.7 Hz), 80.2, 80.1, 71.6, 65.9, 58.5, 48.5, 45.7, 26.8, 25.4. ^19^F NMR (471 MHz, CDCl_3_) δ -120.3. HRMS (ESI-TOF): *m/z* calcd. for C_22_H_20_FN_2_O_2_: 363.1509 [M+H]^+^; found: 363.1501.

#### 3.2.5. 5-Chloro-1′,2′,3′,5a’,6′,11′,11a’,11b’-octahydrospiro[indoline-3,5′-[6,11]epoxybenzo[f]pyrrolo [2,1-a]isoindol]-2-one (**4e**) 

Eluent = petroleum ether/EtOAc (1:1). white solid (61 mg, 81%). mp: 211–213 °C. ^1^H NMR (500 MHz, DMSO-*d*_6_) δ 10.42 (s, 1H), 7.36 (d, *J* = 9.0 Hz, 1H), 7.34 (s, 1H), 7.31 (d, *J* = 7.0 Hz, 1H), 7.23 (d, *J* = 7.0 Hz, 1H), 7.14 (t, *J* = 7.5 Hz, 1H), 7.09 (t, *J* = 7.5 Hz, 1H), 6.89 (d, *J* = 8.5 Hz, 1H), 5.53 (s, 1H), 5.11 (s, 1H), 4.06–3.83 (m, 1H), 2.67 (d, *J* = 7.5 Hz, 1H), 2.65–2.56 (m, 1H), 2.47 (t, *J* = 8.0 Hz, 1H), 2.28–2.16 (m, 1H), 2.01–1.66 (m, 4H). ^13^C NMR (125 MHz, DMSO-*d*_6_) δ 180.4, 147.7, 145.7, 142.6, 129.5, 127.8, 127.1, 126.7, 125.8, 120.4, 119.4, 111.7, 80.1, 79.6, 70.4, 65.3, 58.0, 48.8, 45.6, 26.8, 25.1. HRMS (ESI-TOF): *m/z* calcd. for C_22_H_20_ClN_2_O_2_: 379.1213 [M+H]^+^; found: 379.1206.

#### 3.2.6. 5-Bromo-1′,2′,3′,5a’,6′,11′,11a’,11b’-octahydrospiro[indoline-3,5′-[6,11]epoxybenzo[f]pyrrolo [2,1-a]isoindol]-2-one (**4f**)

Eluent = petroleum ether/EtOAc (1:1). white solid (71 mg, 84%). mp: 256−258 °C. ^1^H NMR (500 MHz, CDCl_3_) δ 7.77 (s, 1H), 7.68 (d, *J* = 2.0 Hz, 1H), 7.44 (dd, *J* = 8.5, 2.0 Hz, 1H), 7.24 (d, *J* = 8.0 Hz, 1H), 7.20–7.05 (m, 3H), 6.80 (d, *J* = 8.0 Hz, 1H), 5.44 (s, 1H), 5.13 (s, 1H), 4.20–4.15 (m, 1H), 2.89 (d, *J* = 7.5 Hz, 1H), 2.80–2.76 (q, *J* = 7.5 Hz, 1H), 2.71 (t, *J* = 8.0 Hz, 1H), 2.49–2.41 (m, 1H), 2.06–2.00 (m, 1H), 1.95–1.85 (m, 3H). ^13^C NMR (125 MHz, CDCl_3_) δ 181.1, 146.9, 144.9, 140.7, 132.2, 131.2, 129.4, 127.0, 126.7, 119.7, 119.0, 115.3, 111.4, 80.2, 80.0, 70.9, 65.6, 58.5, 48.4, 45.6, 26.9, 25.2. HRMS (ESI-TOF): *m/z* calcd. for C_22_H_20_BrN_2_O_2_: 423.0708 [M+H]^+^; found: 423.0702.

#### 3.2.7. 5-Nitro-1′,2′,3′,5a’,6′,11′,11a’,11b’-octahydrospiro[indoline-3,5′-[6,11]epoxybenzo[f]pyrrolo [2,1-a]isoindol]-2-one (**4g**) 

Eluent = petroleum ether/EtOAc (2:1). white solid (69 mg, 89%). mp: 176−178 °C. ^1^H NMR (500 MHz, DMSO-*d*_6_) δ 11.00 (s, 1H), 8.28 (dd, *J* = 8.5, 2.0 Hz, 1H), 8.20 (d, *J* = 2.5 Hz, 1H), 7.32 (d, *J* = 7.0 Hz, 1H), 7.19 (d, *J* = 7.0 Hz, 1H), 7.15 (t, *J* = 7.0 Hz, 1H), 7.11–7.08 (m, 1H), 7.07 (d, *J* = 8.5 Hz, 1H), 5.57 (s, 1H), 5.17 (s, 1H), 4.00–3.96 (m, 1H), 2.75 (d, *J* = 7.5 Hz, 1H), 2.69–2.56 (m, 1H), 2.49 (d, *J* = 7.5 Hz, 1H), 2.25 (td, *J* = 8.5, 3.5 Hz, 1H), 1.97–1.79 (m, 3H), 1.79–1.69 (m, 1H). ^13^C NMR (125 MHz, DMSO-*d*_6_) δ 181.1, 150.2, 147.8, 145.6, 142.5, 128.4, 127.1, 126.9, 126.7, 123.4, 120.4, 119.4, 110.4, 80.1, 79.6, 69.9, 65.4, 58.2, 48.8, 45.5, 26.8, 25.1. HRMS (ESI-TOF): *m/z* calcd. for C_22_H_20_N_3_O_4_: 390.1454 [M+H]^+^; found:390.1449.

#### 3.2.8. 6-Methoxy-1′,2′,3′,5a’,6′,11′,11a’,11b’-octahydrospiro[indoline-3,5′-[6,11]epoxybenzo[f]pyrrolo [2,1-a]isoindol]-2-one (**4h**) 

Eluent = petroleum ether/EtOAc (2:1). white solid (66mg, 88%). mp: 196−198 °C. ^1^H NMR (400 MHz, CDCl_3_) δ 8.82 (s, 1H), 7.46 (d, *J* = 8.0 Hz, 1H), 7.28 (d, *J* = 7.2 Hz, 1H), 7.19–7.07 (m, 3H), 6.63 (dd, *J* = 8.4, 2.4 Hz, 1H), 6.55 (d, *J* = 2.4 Hz, 1H), 5.45 (s, 1H), 5.12 (s, 1H), 4.22–4.19 (m, 1H), 3.85 (s, 3H), 2.91 (d, *J* = 8.0 Hz, 1H), 2.86 (t, *J* = 8.0 Hz, 1H), 2.80 (t, *J* = 8.0 Hz, 1H), 2.55 (td, *J* = 8.8, 3.2 Hz, 1H), 2.08–1.95 (m, 3H), 1.90–1.83 (m, 1H). ^13^C NMR (125 MHz, CDCl_3_) δ 182.7, 160.8, 146.8, 145.3, 143.3, 129.2, 126.9, 126.6, 119.6, 119.0, 118.5, 107.1, 97.4, 80.3, 80.1, 71.4, 65.6, 58.1, 55.5, 48.9, 46.6, 26.7, 25.7. HRMS (ESI-TOF): *m/z* calcd. for C_23_H_23_N_2_O_3_: 375.1709 [M+H]^+^; found: 375.1700.

#### 3.2.9. 7-Bromo-1′,2′,3′,5a’,6′,11′,11a’,11b’-octahydrospiro[indoline-3,5′-[6,11]epoxybenzo[f]pyrrolo [2,1-a]isoindol]-2-one (**4i**)

Eluent = petroleum ether/EtOAc (5:1). white solid (73 mg, 87%). mp: 220−223 °C. ^1^H NMR (500 MHz, CDCl_3_) δ 7.49 (d, *J* = 7.5 Hz, 1H), 7.44 (dd, *J* = 8.0, 1.0 Hz, 1H), 7.40 (s, 1H), 7.24 (d, *J* = 9.5 Hz,1H), 7.14 (td, *J* = 7.0, 2.0 Hz, 1H), 7.12–7.06 (m, 2H), 7.01 (t, *J* = 7.5 Hz, 1H), 5.43 (s, 1H), 5.11 (s, 1H), 4.17–4.15 (m, 1H), 2.90 (d, *J* = 7.5 Hz, 1H), 2.79–2.72 (m, 1H), 2.71 (t, *J* = 8.0 Hz, 1H), 2.45 (td, *J* = 8.5, 4.5 Hz, 1H), 2.04–1.96 (m, 1H), 1.96–1.82 (m, 3H). ^13^C NMR (125 MHz, CDCl_3_) δ 180.1, 146.9, 145.0, 140.8, 131.8, 128.8, 127.2, 127.0, 126.7, 123.6, 119.7, 119.0, 103.3, 80.3, 80.0, 72.0, 65.6, 58.7, 48.3, 45.5, 26.9, 25.2. HRMS (ESI-TOF): *m/z* calcd. for C_22_H_20_BrN_2_O_2_: 423.0708 [M+H]^+^; found: 423.0702.

#### 3.2.10. 1-Methyl-1′,2′,3′,5a’,6′,11′,11a’,11b’-octahydrospiro[indoline-3,5′-[6,11]epoxybenzo[f]pyrrolo [2,1-a]isoindol]-2-one (**4j**)

Eluent = petroleum ether/EtOAc (1:1). white solid (67 mg, 94%). mp: 195−197 °C. ^1^H NMR (400 MHz, CDCl_3_) δ 7.58 (dd, *J* = 7.6, 1.2 Hz, 1H), 7.38 (td, *J* = 7.6, 1.2 Hz, 1H), 7.24 (d, *J* = 6.8 Hz, 1H), 7.15–7.09 (m, 2H), 7.07–7.04 (m, 2H), 6.88 (d, *J* = 8.0 Hz, 1H), 5.45 (s, 1H), 5.18 (s, 1H), 4.29–4.23 (m, 1H), 3.17 (s, 3H), 2.88 (dt, *J* = 8.4, 7.2 Hz, 1H), 2.81 (d, *J* = 7.6 Hz, 1H), 2.73 (t, *J* = 8.0 Hz, 1H), 2.41 (td, *J* = 8.8, 3.2 Hz, 1H), 2.02–1.92 (m, 3H), 1.89–1.79 (m, 1H). ^13^C NMR (125 MHz, CDCl_3_) δ 179.6, 147.0, 145.4, 144.9, 129.2, 128.0, 126.8, 126.7, 126.5, 122.3, 119.5, 119.0, 108.2, 80.4, 80.1, 70.4, 65.1, 58.3, 49.1, 46.3, 26.9, 26.1, 25.3. HRMS (ESI-TOF): *m/z* calcd. for C_23_H_23_N_2_O_2_: 359.1760 [M+H]^+^; found: 359.1755.

#### 3.2.11. 1-Phenyl-1′,2′,3′,5a’,6′,11′,11a’,11b’-octahydrospiro[indoline-3,5′-[6,11]epoxybenzo[f]pyrrolo [2,1-a]isoindol]-2-one (**4k**)

Eluent = petroleum ether/EtOAc (3:1). white solid (76 mg, 91%). mp: 205−207 °C. ^1^H NMR (400 MHz, CDCl_3_) δ 7.66 (d, *J* = 7.6 Hz, 1H), 7.50 (t, *J* = 7.6 Hz, 2H), 7.41 (d, *J* = 7.6 Hz, 2H), 7.37 (d, *J* = 7.6 Hz, 1H), 7.30 (t, *J* = 7.6 Hz, 1H), 7.25 (d, *J* = 7.6 Hz, 1H), 7.21–7.06 (m, 4H), 6.88 (d, *J* = 8.0 Hz, 1H), 5.49 (s, 1H), 5.33 (s, 1H), 4.29–4,23 (m, 1H), 2.97–2.91 (m, 1H), 2.93 (d, *J* = 7.6 Hz, 1H), 2.71 (t, *J* = 7.6 Hz, 1H), 2.47 (td, *J* = 8.0, 4.0 Hz, 1H), 2.13–1.89 (m, 4H). ^13^C NMR (125 MHz, CDCl_3_) δ 178.7, 147.1, 145.4, 144.7, 134.4, 129.5, 129.1, 128.3, 127.9, 126.9, 126.7, 126.6, 122.8, 119.6, 119.0, 109.5, 80.5, 80.1, 69.8, 65.0, 58.7, 48.5, 45.5, 26.9, 25.0. HRMS (ESI-TOF): *m/z* calcd. for C_28_H_25_N_2_O_2_:421.1916 [M+H]^+^; found: 421.1909.

#### 3.2.12. 1′,2′,3′,5a’,6′,11′,11a’,11b’-Octahydro-2H-spiro[acenaphthylene-1,5′-[6,11]epoxybenzo[f]pyrrolo [2,1-a]isoindol]-2-one (**4m**)

Eluent = petroleum ether/EtOAc (4:1). yellow solid (50 mg, 66%). mp: 228−230 °C. ^1^H NMR (500 MHz, CDCl_3_) δ 8.17 (d, *J* = 8.0 Hz, 1H), 7.98 (d, *J* = 2.5 Hz, 1H), 7.96 (d, *J* = 4.0 Hz, 1H), 7.88 (d, *J* = 7.0 Hz, 1H), 7.78 (d, *J* = 7.0 Hz, 1H), 7.75 (d, *J* = 7.0 Hz, 1H), 7.29 (d, *J* = 6.0 Hz, 1H), 7.15 (t, *J* = 7.5 Hz, 1H), 7.08 (t, *J* = 7.5 Hz, 1H), 7.03 (d, *J* = 7.5 Hz, 1H), 5.52 (s, 1H), 5.26 (s, 1H), 4.34–4.29 (m, 1H), 2.97–2.89 (m, 2H), 2.86 (t, *J* = 8.0 Hz, 1H), 2.40 (td, *J* = 8.5, 3.5 Hz, 1H), 2.18–1.95 (m, 3H), 1.91–1.79 (m, 1H). ^13^C NMR (125 MHz, CDCl_3_) δ 206.9, 146.9, 145.6, 142.9, 136.9, 131.6, 131.3, 130.9, 128.4, 128.2, 126.8, 126.6, 125.1, 124.8, 122.1, 119.5, 119.0, 80.8, 80.3, 74.8, 65.6, 57.9, 50.1, 47.2, 26.9, 25.7. HRMS (ESI-TOF): *m/z* calcd. for C_26_H_22_NO_2_: 380.1651 [M+H]^+^; found: 380.1643.

#### 3.2.13. 1′,2′,3′,5a’,6′,11′,11a’,11b’-Octahydrospiro[indene-2,5′-[6,11]epoxybenzo[f]pyrrolo [2,1-a]isoindole]-1,3-dione (**4n**) 

Eluent = petroleum ether/EtOAc (3:1). yellow solid (52 mg, 73%). mp: 144−146 °C. ^1^H NMR (500 MHz, DMSO-*d*_6_) δ 8.06–8.02 (m, 1H), 7.99 (td, *J* = 5.4, 2.7 Hz, 2H), 7.96 (dq, *J* = 5.8, 2.1, 1.5 Hz, 1H), 7.30 (d, *J* = 7.0 Hz, 1H), 7.20–7.15 (m, 1H), 7.15–7.04 (m, 2H), 5.72 (s, 1H), 5.46 (s, 1H), 3.90 (td, *J* = 8.1, 6.4 Hz, 1H), 3.12 (ddd, *J* = 9.8, 8.2, 6.4 Hz, 1H), 2.64 (d, *J* = 7.5 Hz, 1H), 2.56 (t, *J* = 7.9 Hz, 1H), 2.25 (td, *J* = 7.8, 2.2 Hz, 1H), 2.04 (dtd, *J* = 12.5, 8.6, 6.3 Hz, 1H), 1.88 (dtt, *J* = 11.1, 5.7, 2.9 Hz, 2H), 1.67–1.54 (m, 1H). ^13^C NMR (125 MHz, DMSO-*d*_6_) δ 198.4, 147.4, 146.9, 142.7, 140.5, 137.0, 136.4, 126.8, 126.8, 124.4, 123.6, 119.9, 119.8, 79.4, 78.4, 70.6, 65.4, 55.8, 51.7, 47.7, 27.0, 25.4. HRMS (ESI-TOF): *m/z* calcd. for C_23_H_20_NO_3_: 358.1443 [M+H]^+^; found: 358.1440.

#### 3.2.14. 3′-Benzyl-2′,3′,3a’,4′,9′,9a’-hexahydrospiro[indoline-3,1′-[4,9]epoxybenzo[f]isoindol]-2-oneEluent (**5a**)

Petroleum ether/EtOAc (1:1). white solid (54 mg, 68%). mp: 135−137 °C. ^1^H NMR (500 MHz, CDCl_3_) δ 7.78 (s, 1H), 7.57 (d, *J* = 7.5 Hz, 1H), 7.40 (d, *J* = 7.5 Hz, 2H), 7.37–7.29 (m, 3H), 7.24 (t, *J* = 7.5 Hz, 1H), 7.18–7.09 (m, 5H), 6.88 (d, *J* = 8.0 Hz, 1H), 5.72 (s, 1H), 5.41 (s, 1H), 4.55–4.50 (m, 1H), 3.20 (dd, *J* = 12.0, 6.0 Hz, 1H), 3.16 (dd, *J* = 12.0, 7.0 Hz, 1H), 2.69 (t, *J* = 7.0 Hz, 1H), 2.62 (d, *J* = 6.5 Hz, 1H). ^13^C NMR (125 MHz, CDCl_3_) δ 181.5, 146.8, 145.1, 141.3, 139.3, 129.3, 128.9, 128.8, 128.6, 126.9, 126.6, 126.4, 126.3, 122.8, 119.5, 118.9, 109.9, 81.6, 80.4, 69.4, 59.1, 53.9, 51.7, 37.8. HRMS (ESI-TOF): *m/z* calcd. for C_26_H_23_N_2_O_2_: 395.1760 [M+H]^+^; found: 395.1752.

#### 3.2.15. 3′-(4-Iodobenzyl)-2′,3′,3a’,4′,9′,9a’-hexahydrospiro[indoline-3,1′-[4,9]epoxybenzo[f]isoindol]-2-one (**5b**)

Eluent = petroleum ether/EtOAc (2:1). white solid (59 mg, 57%). mp: 159−161 °C. ^1^H NMR (500 MHz, CDCl_3_) δ 7.65 (d, *J* = 8.5 Hz, 2H), 7.56 (d, *J* = 7.5 Hz, 1H), 7.52 (s, 1H), 7.34–7.29 (m, 1H), 7.22–7.17 (m, 1H), 7.17–7.08 (m, 5H), 6.88 (d, *J* = 7.5 Hz, 1H), 5.67 (s, 1H), 5.42 (s, 1H), 4.49–4.45 (m, 1H), 3.14 (dd, *J* = 14.0, 6.0 Hz, 1H), 3.14 (dd, *J* = 13.5, 8.5 Hz, 1H), 2.70 (t, *J* = 7.0 Hz, 1H), 2.62 (d, *J* = 7.0 Hz, 1H). ^13^C NMR (125 MHz, CDCl_3_) δ 181.4, 146.7, 145.1, 141.3, 139.0, 137.6, 130.9, 129.4, 127.0, 126.7, 126.3, 122.8, 119.5, 118.9, 110.0, 108.0, 91.5, 81.6, 80.4, 69.4, 58.9, 53.9, 51.7, 37.4. HRMS (ESI-TOF): *m/z* calcd. for C_26_H_22_IN_2_O_2_: 521.0726 [M+H]^+^; found: 521.0723.

#### 3.2.16. 3′-(4-Hydroxybenzyl)-1-methyl-2′,3′,3a’,4′,9′,9a’-hexahydrospiro[indoline-3,1′-[4,9]epoxybenzo[f]isoindol]-2-one (**5c**)

Eluent = petroleum ether/EtOAc (1:1). yellow solid (18 mg, 21%). mp: 258−260 °C. ^1^H NMR (400 MHz, CDCl_3_) δ 7.57 (d, *J* = 7.4 Hz, 1H), 7.38 (t, *J* = 7.7 Hz, 1H), 7.17 (dt, *J* = 7.7, 4.0 Hz, 2H), 7.14–7.03 (m, 5H), 6.86 (d, *J* = 7.8 Hz, 1H), 6.61 (d, *J* = 8.1 Hz, 2H), 5.70 (s, 1H), 5.43 (s, 1H), 4.49 (q, *J* = 7.3 Hz, 1H), 3.12 (s, 3H), 3.11–3.06 (m, 1H), 2.98 (dd, *J* = 14.0, 8.5 Hz, 1H), 2.70 (t, *J* = 7.0 Hz, 1H), 2.59 (d, *J* = 6.9 Hz, 1H). ^13^C NMR (125 MHz, CDCl_3_) δ 179.3, 154.8, 146.7, 145.0, 144.3, 130.3, 129.6, 128.0, 127.0, 126.7, 125.8, 123.1, 119.4, 118.9, 115.6, 108.6, 81.7, 80.5, 69.4, 59.9, 53.9, 52.1, 36.8, 26.1. HRMS (ESI-TOF): *m/z* calcd. for C_27_H_25_N_2_O_3_: 425.1865 [M+H]^+^; found: 425.1860.

#### 3.2.17. 3′-Phenethyl-2′,3′,3a’,4′,9′,9a’-hexahydrospiro[indoline-3,1′-[4,9]epoxybenzo[f]isoindol]-2-one (**5d**)

Eluent = petroleum ether/EtOAc (1:1). white solid (41 mg, 50%). mp: 212−214 °C. ^1^H NMR (400 MHz, CDCl_3_) δ 7.84 (s, 1H), 7.50 (d, *J* = 7.6 Hz, 1H), 7.35–7.27 (m, 5H), 7.25–7.21 (m, 1H), 7.20–7.06 (m, 5H), 6.91 (d, *J* = 7.6 Hz, 1H), 5.54 (s, 1H), 5.41 (s, 1H), 4.18–4.11 (m, 1H), 2.94 (ddd, *J* = 14.0, 10.0, 6.0 Hz, 1H), 2.81 (ddd, *J* = 14.0, 10.0, 6.4 Hz, 1H), 2.68 (t, *J* = 7.2 Hz, 1H), 2.63 (d, *J* = 7.2 Hz, 1H), 2.22–2.11 (m, 2H). ^13^C NMR (125 MHz, CDCl_3_) δ 181.0, 146.8, 145.1, 142.1, 141.4, 129.4, 128.5, 128.4, 127.0, 126.7, 126.1, 122.8, 119.5, 118.9, 110.1, 81.7, 80.4, 69.6, 58.9, 54.0, 52.5, 34.4, 33.6. HRMS (ESI-TOF): *m/z* calcd. for C_27_H_25_N_2_O_2_: 409.1916 [M+H]^+^; found: 409.1912.

#### 3.2.18. 3′-(2-(Methylthio)ethyl)-2′,3′,3a’,4′,9′,9a’-hexahydrospiro[indoline-3,1′-[4,9]epoxybenzo[f]isoindol]-2-one (**5e**) 

Eluent = MeOH/DCM (50:1). white solid (57 mg, 75%). mp: 203−205 °C. ^1^H NMR (500 MHz, CDCl_3_) δ 7.58 (s, 1H), 7.48 (d, *J* = 7.5 Hz, 1H), 7.31 (t, *J* = 8.0 Hz, 1H), 7.25 (s, 1H), 7.17–7.08 (m, 4H), 6.90 (d, *J* = 8.0 Hz, 1H), 5.57 (s, 1H), 5.40 (s, 1H), 4.16–4.10 (m, 1H), 3.74–3.70 (m, 1H), 2.76 (td, *J* = 13.0, 5.5 Hz, 1H), 2.72–2.66 (m, 2H), 2.62 (d, *J* = 7.0 Hz, 1H), 2.18 (s, 3H), 2.18–2.13 (m, 1H), 2.12–2.06 (m, 1H). ^13^C NMR (125 MHz, CDCl_3_) δ 180.8, 146.7, 145.1, 141.3, 129.4, 128.6, 127.0, 126.7, 126.0, 122.8, 119.5, 118.9, 110.0, 81.7, 80.4, 69.6, 58.3, 54.0, 52.5, 32.5, 31.7, 15.9. HRMS (ESI-TOF): *m/z* calcd. for C_22_H_23_N_2_O_2_S: 379.1480 [M+H]^+^; found: 379.1474.

#### 3.2.19. 3′-Isopropyl-2′,3′,3a’,4′,9′,9a’-hexahydrospiro[indoline-3,1′-[4,9]epoxybenzo[f]isoindol]-2-one (**5f**)

Eluent = petroleum ether/EtOAc (2:1). White solid (56 mg, 80%). Mp: 259−261 °C. ^1^H NMR (500 MHz, CDCl_3_) δ 7.84 (s, 1H), 7.53 (d, *J* = 7.5 Hz, 1H), 7.32 (td, *J* = 8.0, 1.5 Hz, 1H), 7.28 (d, *J* = 7.0 Hz, 1H), 7.19–7.10 (m, 4H), 6.91 (d, *J* = 8.0 Hz, 1H), 5.60 (s, 1H), 5.39 (s, 1H), 3.72–3.70 (m, 1H), 2.69 (t, *J* = 6.5 Hz, 1H), 2.63 (d, *J* = 7.0 Hz, 1H), 2.04–1.98 (m, 2H), 1.20 (d, *J* = 6.5 Hz, 3H), 1.14 (d, *J* = 6.5 Hz, 3H). ^13^C NMR (125 MHz, CDCl_3_) δ 181.4, 147.3, 145.1, 141.3, 129.3, 128.9, 127.0, 126.6, 126.3, 122.8, 119.6, 118.6, 109.9, 81.8, 80.4, 69.6, 65.5, 53.7, 51.8, 30.1, 21.4, 20.9. HRMS (ESI-TOF): *m/z* calcd. For C_22_H_23_N_2_O_2_: 347.1760 [M+H]^+^; found: 347.1753.

#### 3.2.20. 3′-Isobutyl-2′,3′,3a’,4′,9′,9a’-hexahydrospiro[indoline-3,1′-[4,9]epoxybenzo[f]isoindol]-2-one (**5g**)

Eluent = petroleum ether/EtOAc (2:1). white solid (53mg, 73%). mp: 250−252 °C. ^1^H NMR (400 MHz, CDCl_3_) δ 7.48 (d, *J* = 7.6 Hz, 1H), 7.31 (dd, *J* = 8.0, 6.8 Hz, 1H), 7.24 (d, *J* = 8.0, Hz,1H), 7.16–7.07 (m, 4H), 6.89 (d, *J* = 7.6 Hz, 1H), 5.57 (s, 1H), 5.40 (s, 1H), 4.15–4.10 (m, 1H), 2.66–2.60 (m, 2H), 1.87–1.81 (m, 1H), 1.72–1.67 (m, 2H), 1.03 (d, *J* = 6.4 Hz, 3H), 1.02 (d, *J* = 6.8 Hz, 3H). ^13^C NMR (125 MHz, CDCl_3_) δ 181.0, 147.0, 145.2, 141.4, 129.3, 128.7, 127.0, 126.6, 126.0, 122.7, 119.5, 118.8, 110.0, 81.7, 80.4, 69.7, 56.6, 54.0, 52.7, 40.5, 26.4, 23.3, 22.8. HRMS (ESI-TOF): *m/z* calcd. for C_23_H_25_N_2_O_2_: 361.1916 [M+H]^+^; found: 361.1907.

#### 3.2.21. 3′-(*sec*-Butyl)-2′,3′,3a’,4′,9′,9a’-hexahydrospiro[indoline-3,1′-[4,9]epoxybenzo[f]isoindol]-2-one (**5h**)

For major isomer: Eluent = petroleum ether/EtOAc (2:1). green solid (42 mg, 59%). mp: 141−143 °C. ^1^H NMR (400 MHz, CDCl_3_) δ 7.78 (s, 1H), 7.51 (d, *J* = 7.6 Hz, 1H), 7.34–7.26 (m, 2H), 7.19–7.07 (m, 4H), 6.88 (d, *J* = 7.6 Hz, 1H), 5.53 (s, 1H), 5.37 (s, 1H), 3.81–3.77 (m, 1H), 2.68 (t, *J* = 6.8 Hz, 1H), 2.61 (d, *J* = 6.8 Hz, 1H), 1.80–1.71 (m, 2H), 1.41–1.30 (m, 1H), 1.10–1.04 (m, 6H). ^13^C NMR (125 MHz, CDCl_3_) δ 181.5, 147.3, 145.1, 141.4, 129.3, 128.9, 127.0, 126.6, 126.2, 122.7, 119.6, 118.6, 110.0, 81.9, 80.4, 69.6, 63.5, 53.4, 51.9, 36.0, 27.2, 16.8, 10.5. HRMS (ESI-TOF): *m/z* calcd. for C_23_H_25_N_2_O_2_: 361.1916 [M+H]^+^; found: 361.1910. 

For minor isomer: Eluent = petroleum ether/EtOAc (2:1). green solid (25 mg, 35%). mp: 141−143 °C. ^1^H NMR (400 MHz, CDCl_3_) δ 7.71 (s, 1H), 7.51 (d, *J* = 7.2 Hz, 1H), 7.33–7.26 (m, 2H), 7.18–7.06 (m, 4H), 6.89 (d, *J* = 8.0 Hz, 1H), 5.58 (s, 1H), 5.37 (s, 1H), 3.81–3.77 (m, 1H), 2.66 (t, *J* = 6.8 Hz, 1H), 2.60 (d, *J* = 6.8 Hz, 1H), 1.82–1.70 (m, 2H), 1.34–1.28 (m, 1H), 1.15 (d, *J* = 6.4 Hz, 3H), 0.94 (t, *J* = 7.2 Hz, 3H). ^13^C NMR (125 MHz, CDCl_3_) δ 181.4, 147.2, 145.1, 141.3, 129.3, 129.0, 127.0, 126.6, 126.3, 122.7, 119.6, 118.6, 109.9, 81.8, 80.6, 69.4, 64.0, 53.6, 51.7, 36.5, 27.2, 17.3, 11.3. HRMS (ESI-TOF): *m/z* calcd. for C_23_H_25_N_2_O_2_: 361.1916 [M+H]^+^; found: 361.1908.

#### 3.2.22. 1′,5a’,6′,11′,11a’,11b’-Hexahydro-3′H-spiro[indoline-3,5′-[6,11]epoxybenzo[f]thiazolo [4,3-a]isoindol]-2-one (**5j**)

Eluent = petroleum ether/EtOAc (1:1). white solid (57 mg, 78%). mp: 157−159 °C. ^1^H NMR (500 MHz, CDCl_3_) δ 9.04 (s, 1H), 7.58 (d, *J* = 7.5 Hz, 1H), 7.33 (td, *J* = 7.5, 1.0 Hz, 1H), 7.30 (d, *J* = 7.0 Hz, 1H), 7.19–7.17 (m, 1H), 7.16–7.11 (m, 3H), 6.97 (dd, *J* = 8.0, 1.0 Hz, 1H), 5.41 (s, 1H), 4.86 (s, 1H), 4.30 (dt, *J* = 10.5, 6.5 Hz, 1H), 3.96 (d, *J* = 8.5 Hz, 1H), 3.39 (d, *J* = 8.5 Hz, 1H), 3.28 (t, *J* = 10.0 Hz, 1H), 3.09–3.04 (m, 3H). ^13^C NMR (125 MHz, CDCl_3_) δ 181.7, 145.9, 144.8, 141.6, 129.8, 128.6, 127.1, 126.9, 124.8, 122.9, 119.7, 119.3, 110.3, 80.0, 79.6, 72.5, 70.1, 58.2, 50.2, 47.4, 31.8. HRMS (ESI-TOF): *m/z* calcd. for C_22_H_19_N_2_O_2_S: 363.1167 [M+H]^+^; found: 363.1163.

#### 3.2.23. 2′-Cyclohexyl-1′,2′,3′,5a’,6′,11′,11a’,11b’-octahydrospiro[indoline-3,5′-[6,11]epoxybenzo[f]pyrrolo [2,1-a]isoindol]-2-one (**5k**)

For major isomer: Eluent =petroleum ether/EtOAc (2:1). white solid (39 mg, 46%). mp: 141−143 °C. ^1^H NMR (400 MHz, CDCl_3_) δ 8.48 (s, 1H), 7.56 (d, *J* = 7.6 Hz, 1H), 7.29 (t, *J* = 8.0 Hz, 1H), 7.24 (d, *J* = 7.2 Hz, 1H), 7.15–7.05 (m, 4H), 6.91 (d, *J* = 8.0 Hz, 1H), 5.42 (s, 1H), 5.15 (s, 1H), 4.13–4.11 (m, 1H), 2.85 (d, *J* = 7.6 Hz, 1H), 2.79 (t, *J* = 7.6 Hz, 1H), 2.67 (t, *J* = 7.6 Hz, 1H), 2.24 (dd, *J* = 10.0, 6.8 Hz, 1H), 2.14–2.06 (m, 2H), 1.84–1.45 (m, 7H), 1.16–1.07 (m, 3H), 0.96–0.74 (m, 2H). ^13^C NMR (125 MHz, CDCl_3_) δ 181.6, 147.3, 145.2, 141.9, 129.1, 128.3, 127.4, 126.8, 126.5, 122.4, 119.6, 118.8, 110.0, 80.6, 80.0, 70.9, 64.8, 57.8, 50.0, 48.5, 46.3, 42.4, 31.8, 31.4, 29.5, 26.7, 26.3, 26.2. HRMS (ESI-TOF): *m/z* calcd. for C_28_H_31_N_2_O_2_: 427.2386 [M+H]^+^; found: 427.2380. 

For minor isomer: Eluent =petroleum ether/EtOAc (3:1). white solid (36 mg, 42%). mp: 141−143 °C. ^1^H NMR (400 MHz, CDCl_3_) δ 7.93 (s, 1H), 7.55 (d, *J* = 7.2 Hz, 1H), 7.31 (t, *J* = 7.6 Hz, 1H), 7.24 (d, *J* = 7.2 Hz, 1H), 7.16–7.04 (m, 4H), 6.91 (d, *J* = 8.0 Hz, 1H), 5.47 (s, 1H), 5.20 (s, 1H), 4.37–4.31 (m, 1H), 2.81 (d, *J* = 7.6 Hz, 1H), 2.76 (d, *J* = 8.8 Hz, 1H), 2.72 (t, *J* = 8.0 Hz, 1H), 2.56 (t, *J* = 7.2 Hz, 1H), 2.12–1.98 (m, 2H), 1.83–1.50 (m, 7H), 1.21–1.05 (m, 3H), 0.99–0.82 (m, 2H). ^13^C NMR (126 MHz, CDCl_3_) δ 181.9, 146.8, 145.6, 141.9, 129.2, 128.9, 126.9, 126.9, 126.6, 122.1, 119.5, 119.0, 110.1, 80.3, 80.2, 71.0, 65.2, 58.2, 51.6, 49.9, 47.8, 42.1, 32.2, 31.9, 31.0, 26.5, 26.2, 26.1. HRMS (ESI-TOF): *m/z* calcd. for C_28_H_31_N_2_O_2_: 427.2386 [M+H]^+^; found: 427.2382.

#### 3.2.24. 5-Bromo-1′,5a’,6′,11′,11a’,11b’-hexahydro-3′H-spiro[indoline-3,5′-[6,11]epoxybenzo[f]thiazolo [4,3-a]isoindol]-2-one (**6**)

Eluent = petroleum ether/EtOAc (2:1). white solid (2.14 g, 61%). mp: 280−282 °C. ^1^H NMR (400 MHz, DMSO-*d*_6_) δ 10.63 (s, 1H), 7.49 (dd, *J* = 8.4, 2.0 Hz, 1H), 7.44 (d, *J* = 2.0 Hz, 1H), 7.33 (d, *J* = 7.2 Hz, 1H), 7.25 (d, *J* = 6.8 Hz, 1H), 7.20–7.08 (m, 2H), 6.87 (d, *J* = 8.4 Hz, 1H), 5.53 (s, 1H), 4.82 (s, 1H), 4.06–4.00 (m, 1H), 3.71 (d, *J* = 8.8 Hz, 1H), 3.18 (d, *J* = 8.4 Hz, 1H), 3.05–2.94 (m, 2H), 2.83 (t, *J* = 8.0 Hz, 1H), 2.76 (d, *J* = 8.4 Hz, 1H). ^13^C NMR (125 MHz, DMSO-*d*_6_) δ 179.4, 146.8, 145.5, 143.0, 132.6, 131.0, 127.8, 127.2, 127.0, 120.5, 119.7, 113.8, 112.1, 79.7, 79.4, 71.9, 70.3, 57.9, 50.1, 47.3, 31.7. HRMS (ESI-TOF): *m/z* calcd. for C_21_H_18_BrN_2_O_2_S: 441.0272 [M+H]^+^; found: 441.0268.

### 3.3. General Procedure for Deoxyaromatization of Cycloaddition Product ***6***

A mixture of the cycloaddition product **6** (44 mg, 0.1 mmol) and DCM (1 mL) was added to a 25 mL sealed tube at 0 °C. Later, triflic acid (0.1 mL) was added to the system dropwise. Then, the reaction mixture was stirred at room temperature for 3 h. After the reaction was completed, the reaction mixture was diluted with 5.0 mL of DCM and filtered through a plug of Celite, followed by washing with 2 mL of saturated NaHCO_3_ (aq.). and extracted with DCM three times. The combined organic layers were dried over Na_2_SO_4_ and filtered, and the solvent was removed under reduced pressure to give the crude product, which was purified by column chromatography on silica gel.

#### 5′-Bromo-1,11b-dihydro-3H-spiro[benzo[f]thiazolo [4,3-a]isoindole-5,3′-indolin]-2′-one (**7**) 

Eluent = petroleum ether/EtOAc (2:1). white solid (32 mg, 75%). mp: 233−235 °C. ^1^H NMR (400 MHz, DMSO-*d*_6_) δ 10.93 (s, 1H), 8.10 (s, 1H), 8.05 (d, *J* = 8.0 Hz, 1H), 7.94 (d, *J* = 8.0 Hz, 1H), 7.63–7.57 (m, 2H), 7.54 (ddd, *J* = 8.4, 6.8, 1.2 Hz, 1H), 7.44 (s, 1H), 7.14 (d, *J* = 2.0 Hz, 1H), 7.07 (d, *J* = 8.4 Hz, 1H), 5.29 (t, *J* = 7.2 Hz, 1H), 4.28 (d, *J* = 10.4 Hz, 1H), 3.81 (d, *J* = 10.4 Hz, 1H), 3.69 (dd, *J* = 10.4, 7.2 Hz, 1H), 2.88 (dd, *J* = 10.8, 7.2 Hz, 1H). ^13^C NMR (125 MHz, CDCl_3_) δ 183.0, 147.2, 145.7, 144.6, 138.5, 138.3, 138.0, 135.5, 134.2, 133.2, 133.1, 131.6, 131.2, 126.5, 126.0, 118.8, 117.4, 81.6, 78.3, 59.3, 44.7. HRMS (ESI-TOF): *m/z* calcd. for C_21_H_16_BrN_2_OS: 423.0167 [M+H]^+^; found: 423.0162.

### 3.4. General Procedure for the Synthesis of N-Boc Substituted Spirooxindole ***8***

The cycloaddition product **6** (44 mg, 0.1 mmol), DMAP (2.4 mg, 0.02 mmol) and THF were added to a 25 mL sealed tube at 0 °C. Later, the mixture of (Boc)_2_O (0.12 mmol) and THF (1 mL) was added to the system dropwise. Then, the reaction mixture was stirred at room temperature for 2 h. After the reaction was completed, the reaction mixture was diluted with 10.0 mL of EtOAc and filtered through a plug of Celite, followed by washing with 2 mL of H_2_O three times, and the organic phase was dried with anhydrous sodium sulfate and filtered, and the solvent was removed under reduced pressure to give the crude product, which was purified by column chromatography on silica gel.

#### *tert*-Butyl -5-bromo-2-oxo-1′,5a’,6′,11′,11a’,11b’-hexahydro-3′H-spiro[indoline-3,5′-[6,11]epoxybenzo[f]thiazolo [4,3-a]isoindole]-1-carboxylate (**8**)

Eluent = petroleum ether/EtOAc (5:1). white solid (51 mg, 93%). mp: 182−184 °C. ^1^H NMR (400 MHz, CDCl_3_) δ 7.86 (d, *J* = 8.4 Hz, 1H), 7.75 (d, *J* = 2.4 Hz, 1H), 7.55 (dd, *J* = 8.4, 2.0 Hz, 1H), 7.29–7.27 (m, 1H), 7.14 -7.09 (m, 3H), 5.44 (s, 1H), 5.01 (s, 1H), 4.31 (dt, *J* = 10.8, 6.8 Hz, 1H), 3.68 (d, *J* = 6.8 Hz, 1H), 3.34 (d, *J* = 6.8 Hz, 1H), 3.13 (t, *J* = 9.6 Hz, 1H), 2.99–2.95 (m, 2H), 2.83 (t, *J* = 8.0 Hz, 1H), 1.62 (s, 9H). ^13^C NMR (125 MHz, CDCl_3_) δ 176.20, 148.94, 146.14, 144.35, 139.38, 133.02, 130.31, 127.20, 126.96, 126.65, 119.78, 119.18, 118.08, 116.60, 85.09, 79.81, 79.74, 69.76, 68.90, 59.84, 46.95, 45.98, 30.32, 28.06. HRMS (ESI-TOF): *m/z* calcd. for C_26_H_26_BrN_2_O_4_S: 541.0797 [M+H]^+^; found: 541.0796. 

### 3.5. General Procedure for the Reduction of Cycloadduct ***6*** with Lithium Aluminum Hydride 

To a solution of LiAlH_4_ (43 mg, 1.1 mmol) in anhydrous diethyl ether (2 mL) under an argon atmosphere was added cycloadduct **6** (50 mg, 0.11 mmol), and the reaction mixture was stirred at room temperature for 4 h. After that, the mixture was cooled to 0 °C and 0.5 mL of H_2_O was added to this mixture. Then, 0.5 mL of a 10% NaOH solution and 0.5 mL of another portion of H_2_O were added to it. The reaction mixture was stirred for 5 min at ambient temperature. The organic layer was separated, and the aqueous phase was extracted with EtOAc (3 × 5 mL). The total organic mixture was dried over anhydrous Na_2_SO_4_ and concentrated under reduced pressure to give the crude product, which was purified by column chromatography on silica gel.

#### 5-Bromo-1′,5a’,6′,11′,11a’,11b’-hexahydro-3′H-spiro[indoline-3,5′-[6,11]epoxybenzo[f]thiazolo [4,3-a]isoindol]-2-ol (**9**)

Eluent = petroleum ether/EtOAc (3:1). white solid (33 mg, 64%). mp: 147−149 °C.^1^H NMR (500 MHz, CDCl_3_) δ 7.46 (d, *J* = 2.0 Hz, 1H), 7.29–7.25 (m, 3H), 7.14–7.07 (m, 4H), 6.60 (d, *J* = 8.5 Hz, 1H), 5.53 (s, 1H), 5.07 (s, 1H), 3.78 (d, *J* = 9.0 Hz, 1H), 3.10–3.06 (m, 1H), 2.94 (d, *J* = 9.0 Hz, 1H), 2.83–2.76 (m, 2H), 2.62 (t, *J* = 7.0 Hz, 1H), 2.42 (d, *J* = 7.5 Hz, 1H). ^13^C NMR (125 MHz, CDCl_3_) δ 150.7, 146.4, 146.3, 131.9, 129.0, 126.7, 126.6, 119.4, 119.2, 111.6, 110.9, 81.0, 79.5, 77.0, 67.4, 54.3, 51.8, 47.3, 34.5, 24.1. HRMS (ESI-TOF): *m/z* calcd. for C_21_H_20_BrN_2_O_2_S: 443.0429 [M+H]^+^; found: 443.0424.

### 3.6. Crystallography

Crystal data for **5j** C_21_H_18_N_2_O_2_S, M = 450.54 (**5j** + solvent: EtOAc), orthorhombic, a = 13.0295(3) Å, b = 9.2645(2) Å, c = 21.1483(4) Å, α = 90°, β = 94.03°, γ = 90°, V = 2546.54(9) Å3, space group P21/n(14), Mu = 1.382 mm^−1^. Z = 4, T = 267 K, Dx = 1175 g/cm^3^, which were used in all calculations. The final R1 was 0.0443 (I > 2σ(I)) and wR2 was 0.1398 (all data).

The results of the X-ray diffraction analysis for compound **5j** were deposited with the Cambridge Crystallographic Data Centre (CCDC 2243483). 

## 4. Conclusions

Microwave-promoted multicomponent reactions of isatins, α-amino acids and 1,4-dihydro-1,4-epoxynaphthalene have been demonstrated. Under environmentally friendly conditions, a variety of oxygen-bridged spirooxindoles were delivered in good to excellent yields within 15 min. This cascade protocol shows excellent diastereoselectivity and remarkable functional group tolerance, and a diversity of amino acids can be involved in this transformation. This work provides a powerful means to expand the structural diversity of spirooxindole as a promising scaffold for novel drug discovery.

## Figures and Tables

**Table 1:** Optimization of reaction conditions ^a^.

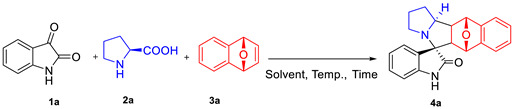				
Entry	Solvent	Tep.(°C)	Time	Yield(100%) ^b^
1	THF	70	15 min	40
2	DMF	70	15 min	33
3	DMSO	70	15 min	46
4	Toluene	70	15 min	8
5	MeOH	70	15 min	73
6	EtOH	70	15 min	60
7	*^i^*PrOH	70	15 min	70
8	*^t^*BuOH	70	15 min	48
9	MeOH	60	15 min	61
10	MeOH	80	15 min	60
11	MeOH	90	15 min	64
12	MeOH	70	20 min	69
13	MeOH	70	25 min	73
14	MeOH	70	30 min	72
15	MeOH	70	10 min	61
16 ^c^	MeOH	70	15 min	81
17 ^d^	MeOH	70	15 min	83

^a^ Reaction conditions: **1a** (0.1 mmol), **2a** (0.1 mmol), and **3a** (0.1 mmol) were sealed in a glass vial without degassing of air and placed in a microwave reactor. ^b^ Isolated yields. ^c^ **1a**:**2a**:**3a** = 1:1:1.2. ^d^ **1a**:**2a**:**3a** = 1:1:1.5.

## Data Availability

The data presented in this study are available on request from the corresponding author.

## Supplementary Materials

The following supporting information can be downloaded at: https://www.mdpi.com/article/10.3390/molecules28083508/s1, including ^1^H, ^13^C NMR and ^19^F spectra of [3+2] cycloaddition products **4**–**6**, and ^1^H and ^13^C NMR NMR spectra of the derivatizations of the gram reaction product **7**–**9**.

## Sample Availability

Samples of the compounds **4**–**9** are available from the authors.
