# Supplementary material for: An Efficient Synthesis of Oxygen-Bridged Spirooxindoles via Microwave-Promoted Multicomponent Reaction
Source: Molecules. 2023 Apr 16;28(8):3508. doi: 10.3390/molecules28083508 (PMC10146779; doi:10.3390/molecules28083508)
Supplement: Supplementary file 1 [file molecules-28-03508-s001.zip › molecules-2318647-supplementary.pdf]

*Supporting Information*

# An Efficient Synthesis of Oxygen-bridged Spirooxindoles via Microwave-Promoted Multicomponent Reaction

Yaojing Shi, Hua Zhao*, Yufen Zhao

Institute of Drug Discovery Technology, Qian Xuesen Collaborative Research Centerof Astrochemistry and Space Life Sciences, Ningbo University, Ningbo 315211, Zhejiang, China

zhaohua@nbu.edu.cn

# 1. $^{1}$H, $^{13}$C and $^{19}$F NMR Spectra of Compound

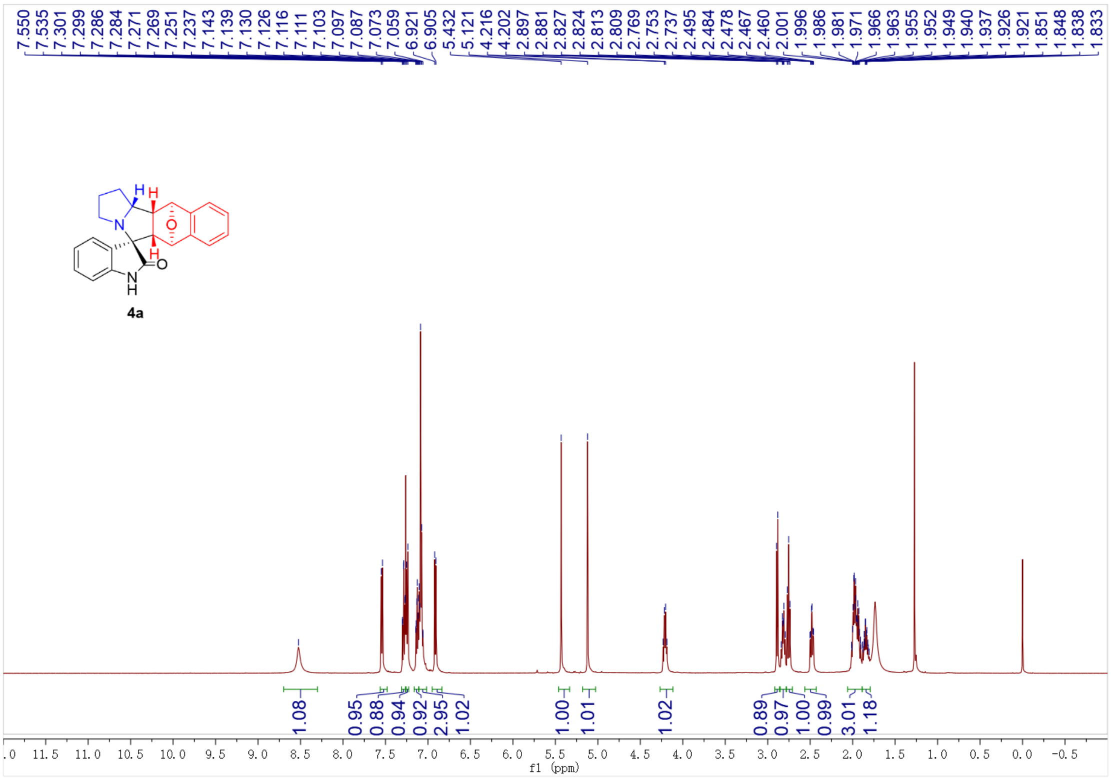


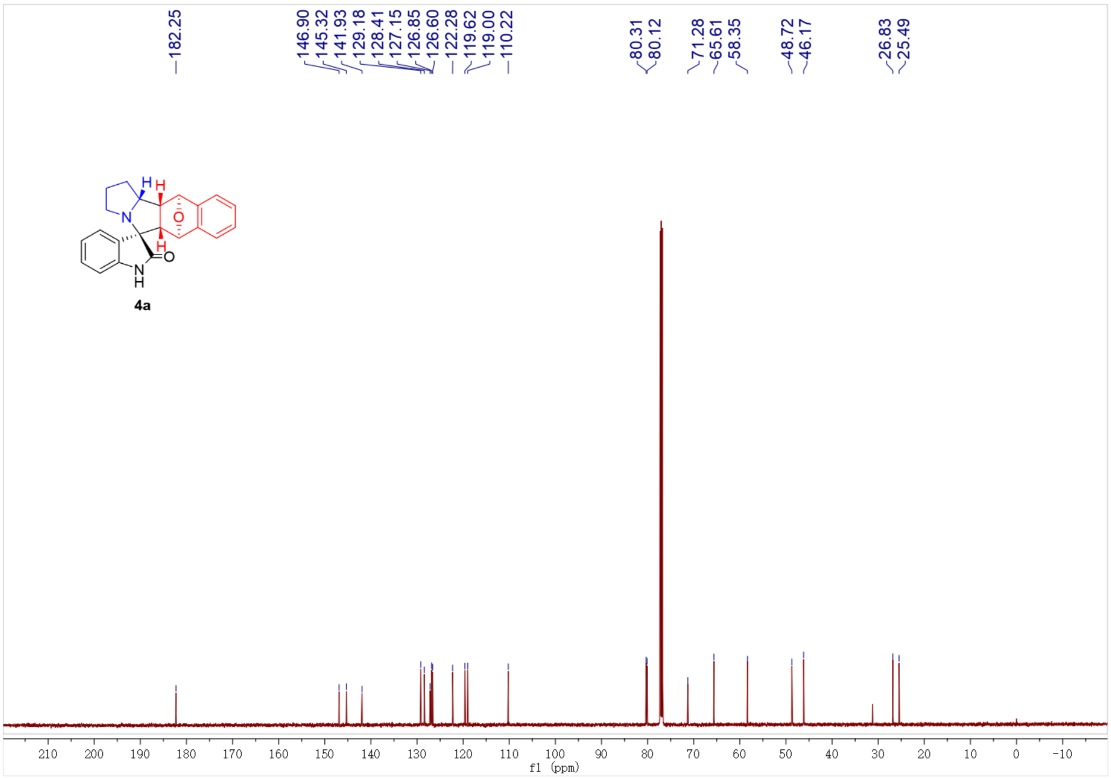


**$^{1}$H NMR** (500 MHz, $CDCl_3$), **$^{13}$C NMR** (125 MHz, $CDCl_3$) spectrum of **4a**

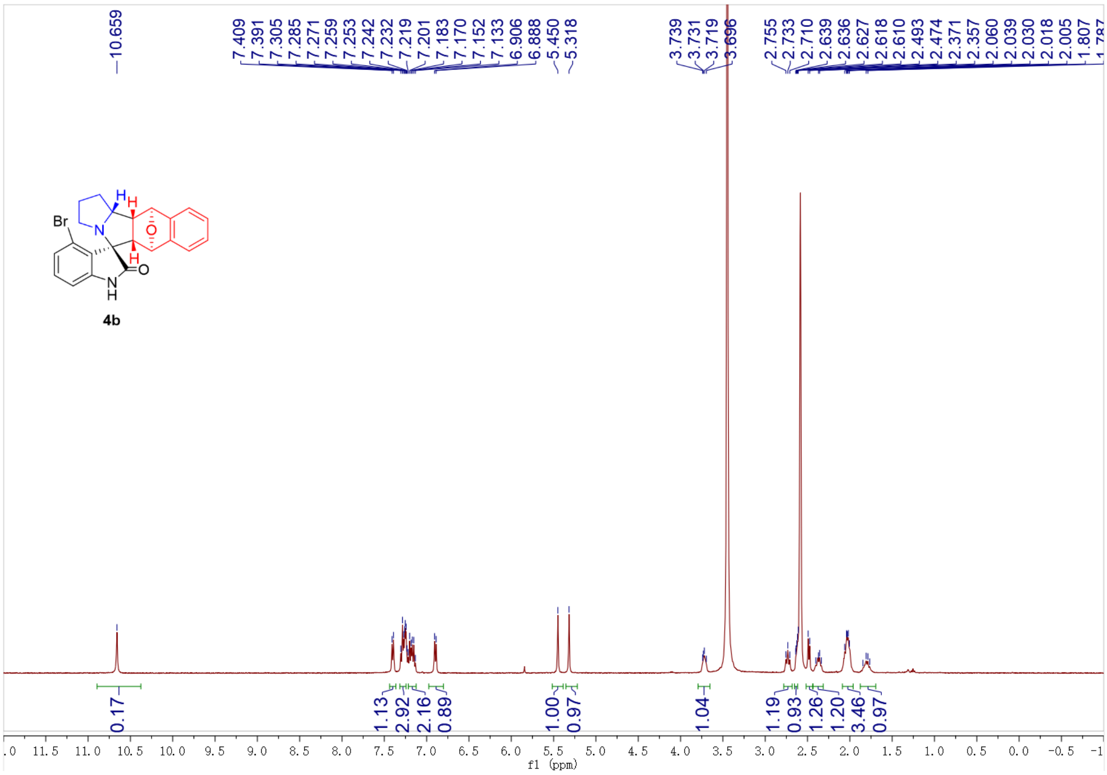


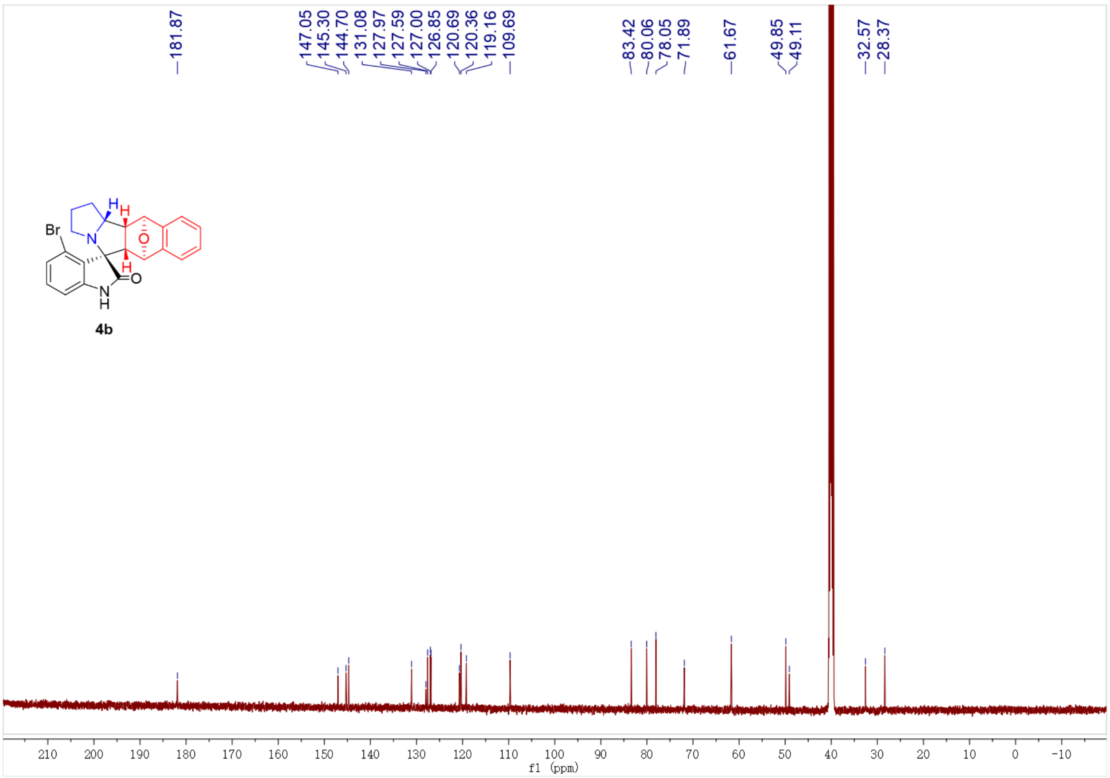


**$^{1}$H NMR** (400 MHz, DMSO-$d_6$), **$^{13}$C NMR** (125 MHz, DMSO-$d_6$) spectrum of **4b**

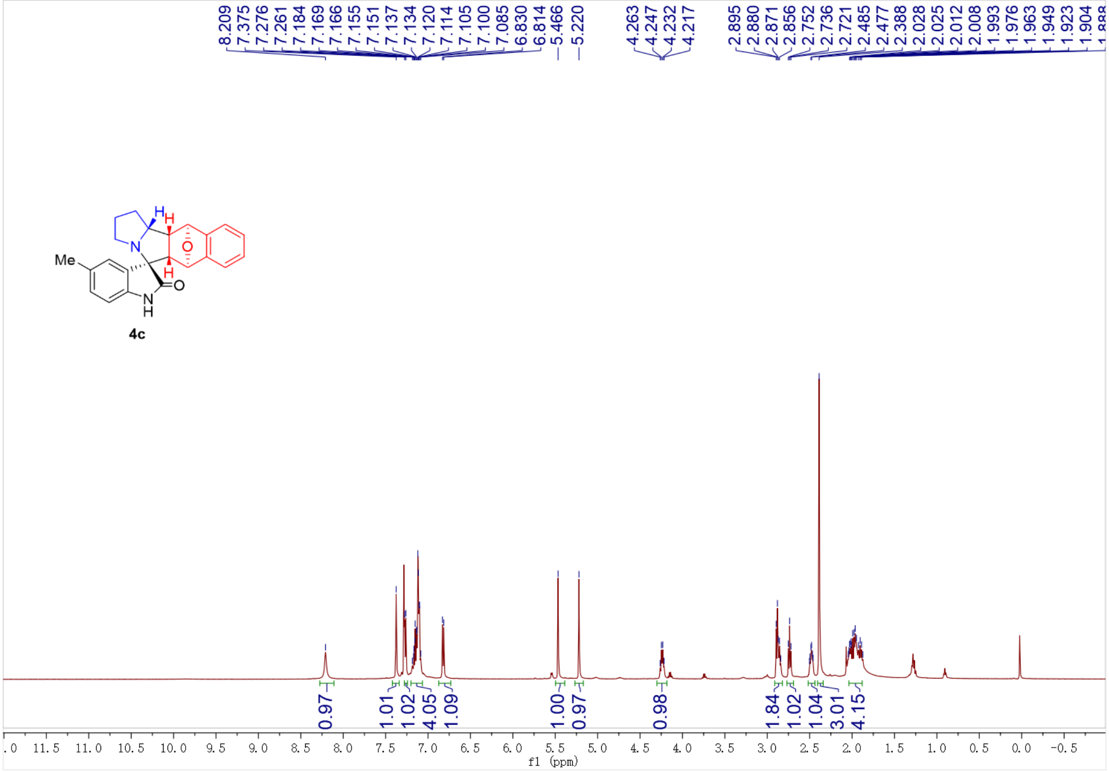


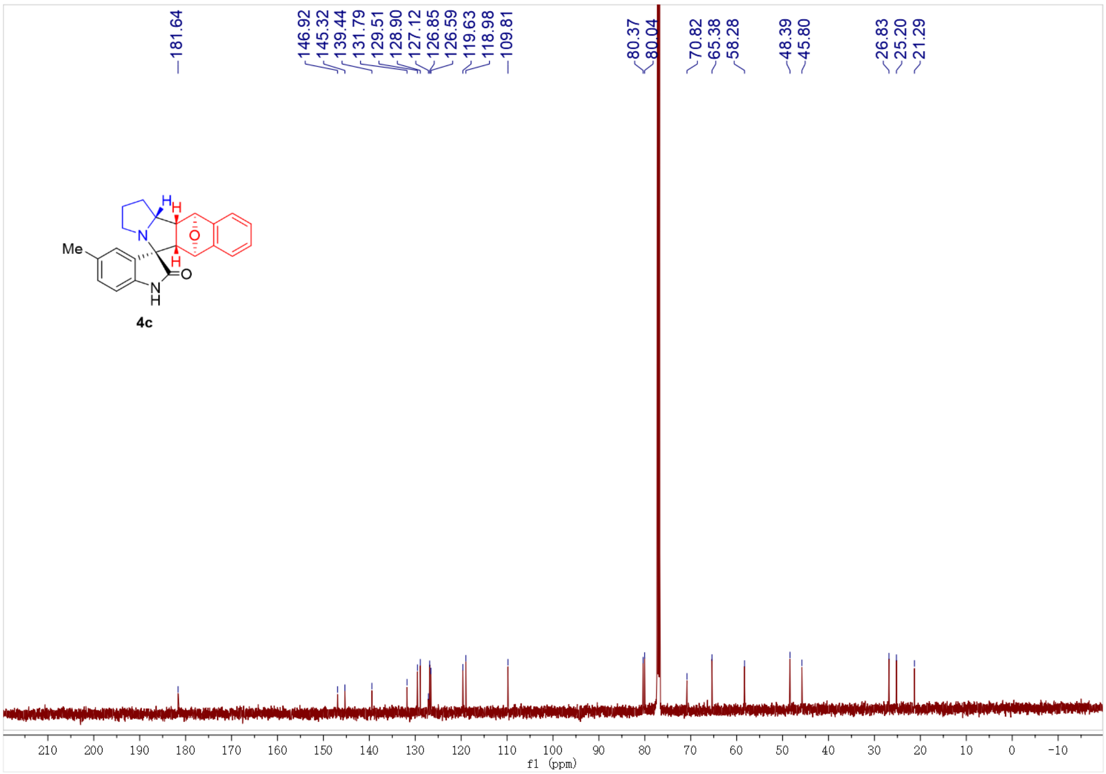


**$^{1}$H NMR** (500 MHz, $CDCl_3$), **$^{13}$C NMR** (125 MHz, $CDCl_3$) spectrum of **4c**

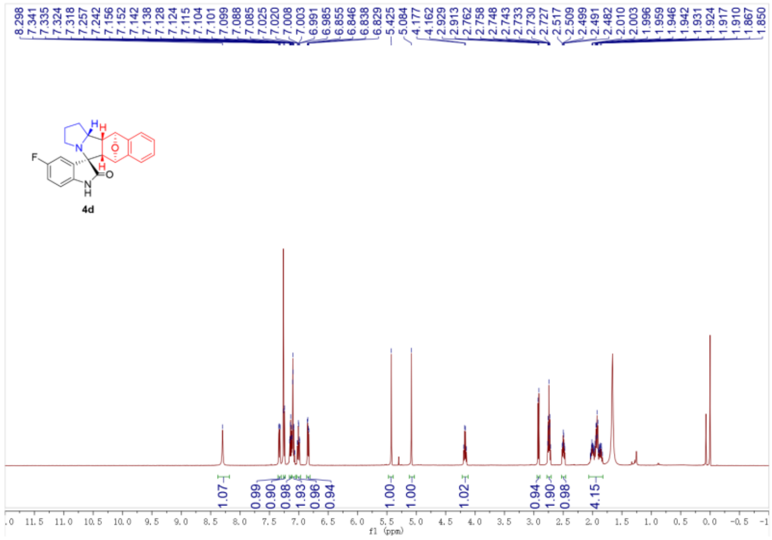


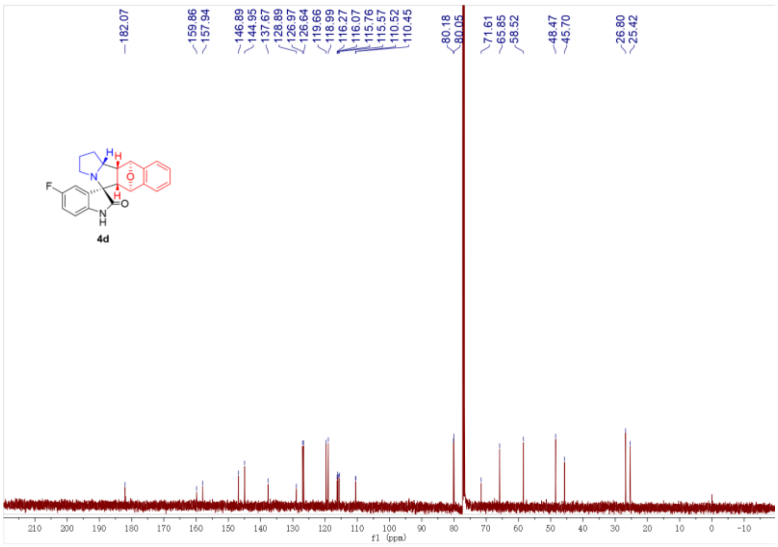


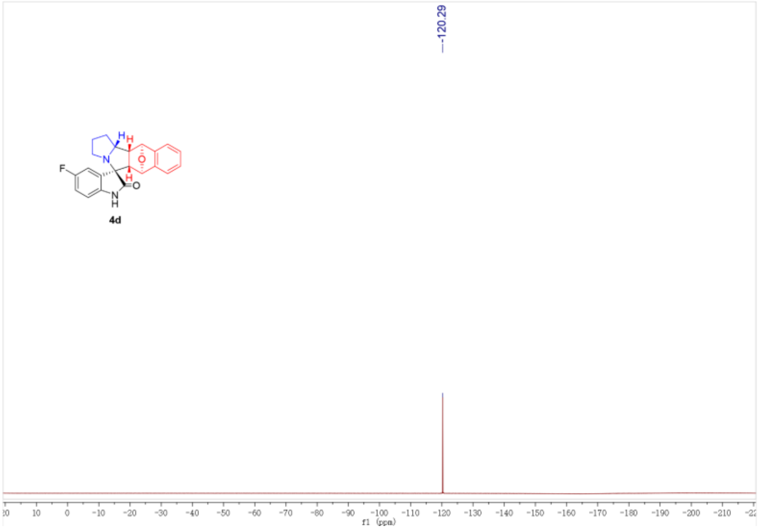


**$^{1}$H NMR** (500 MHz, $CDCl_3$), **$^{13}$C NMR** (125 MHz, $CDCl_3$) and **$^{19}$F NMR** (471 MHz, $CDCl_3$) spectrum of **4d**

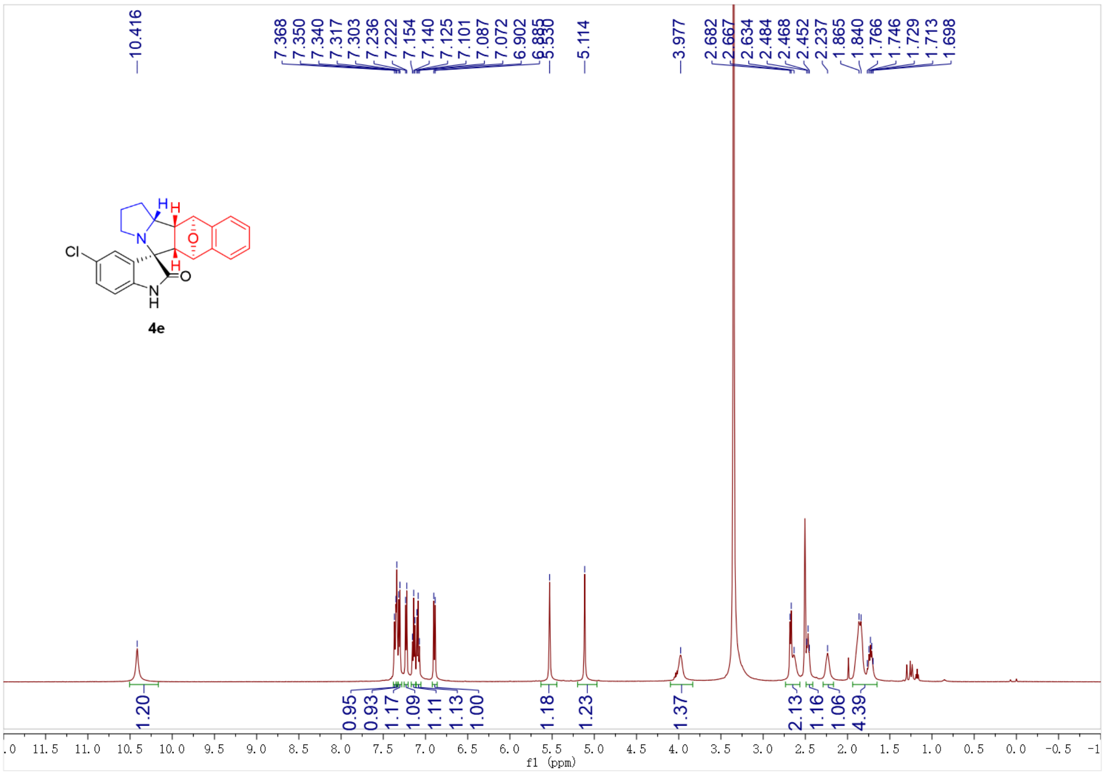


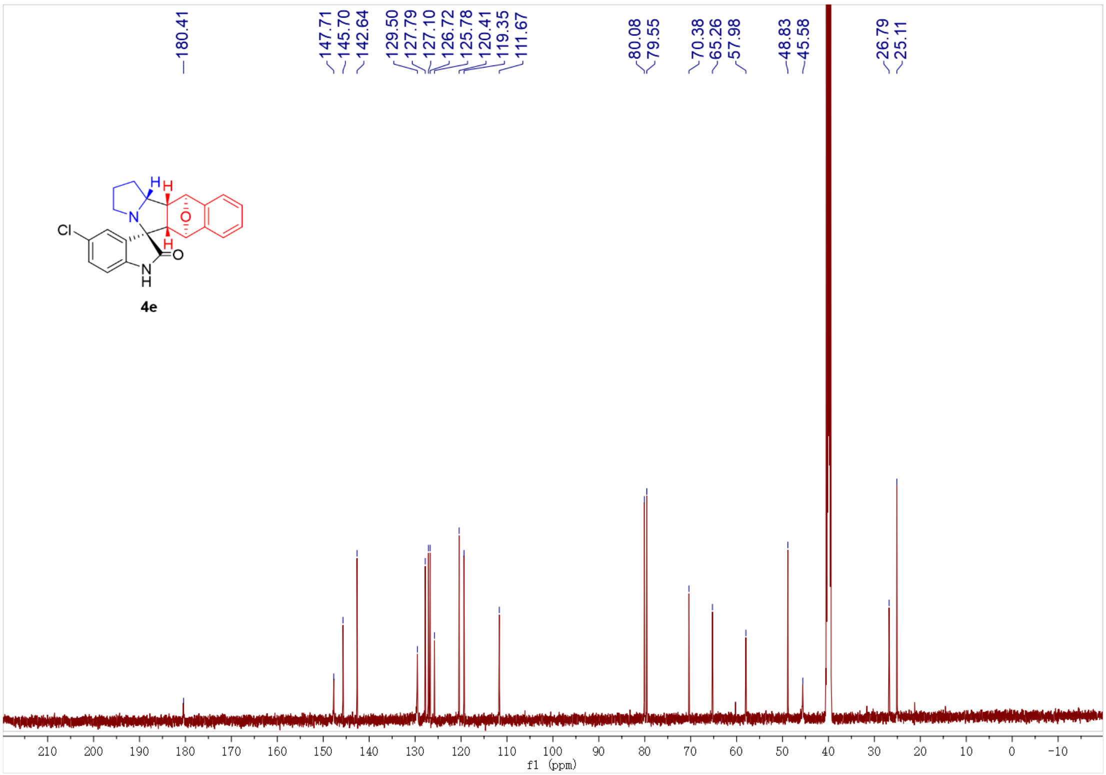


**$^{1}$H NMR** (500 MHz, DMSO-$d_6$), **$^{13}$C NMR** (125 MHz, DMSO-$d_6$) spectrum of **4e**

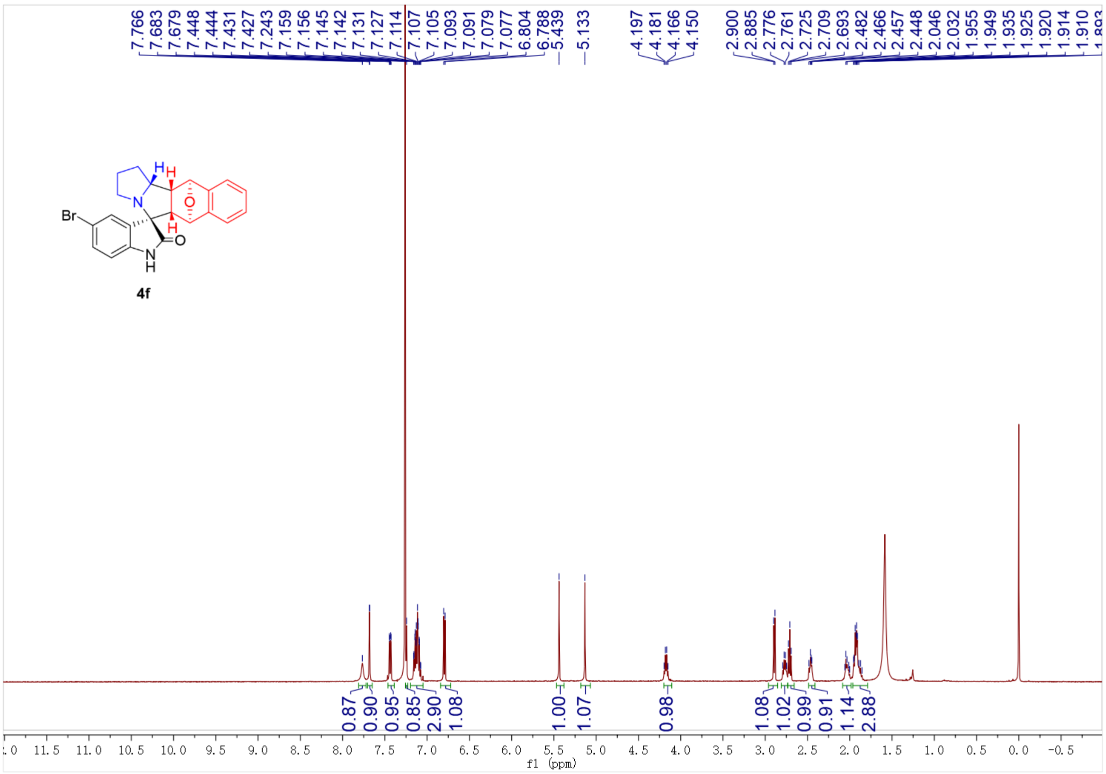

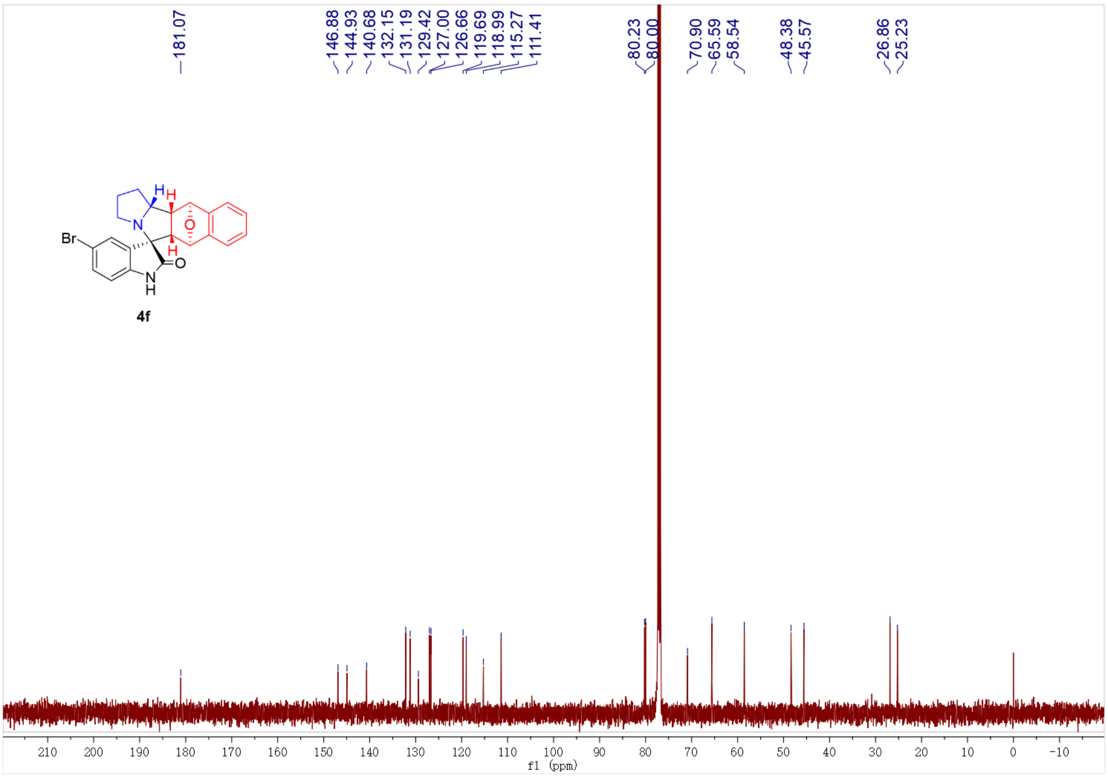

**$^{1}$H NMR** (500 MHz, $CDCl_3$), **$^{13}$C NMR** (125 MHz, $CDCl_3$) spectrum of **4f**

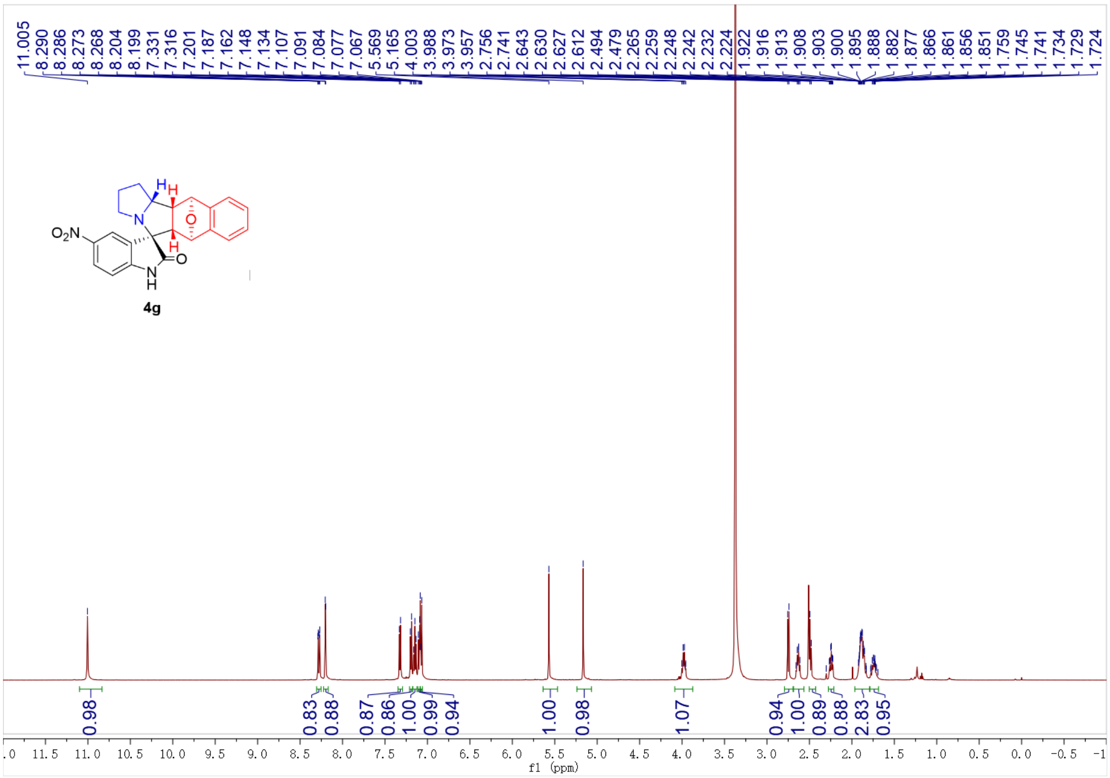


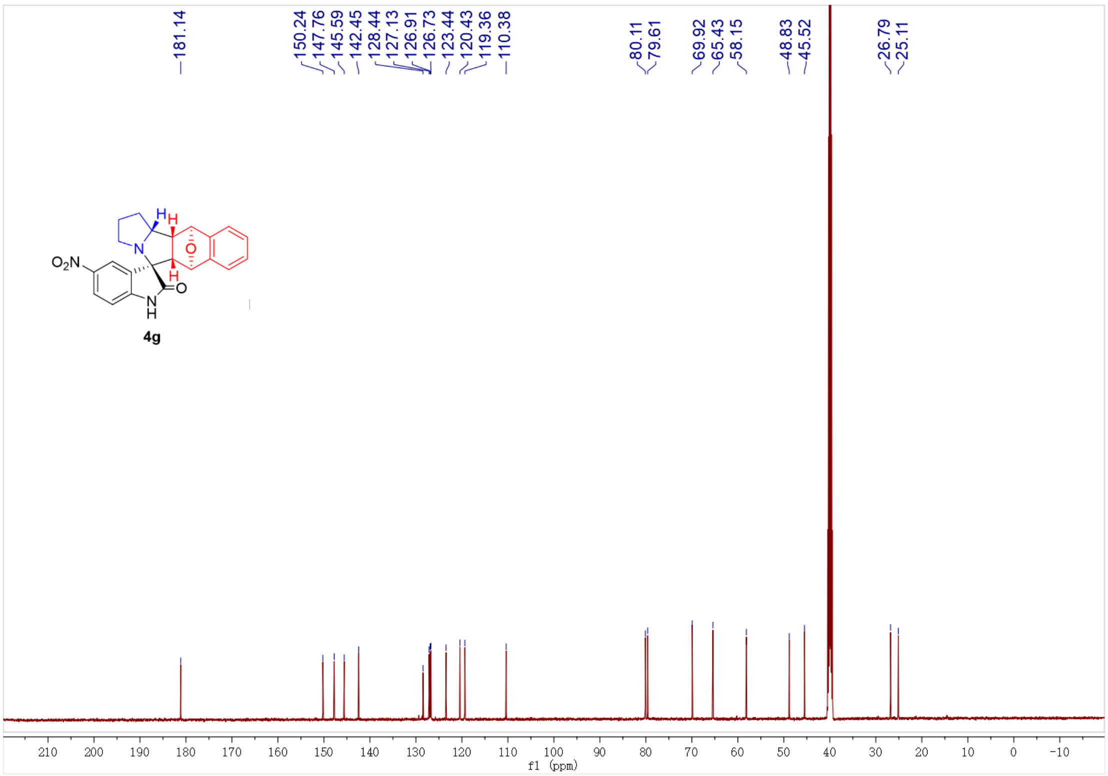


**$^{1}$H NMR** (500 MHz, DMSO-$d_6$), **$^{13}$C NMR** (125 MHz, DMSO-$d_6$) spectrum of **4g**

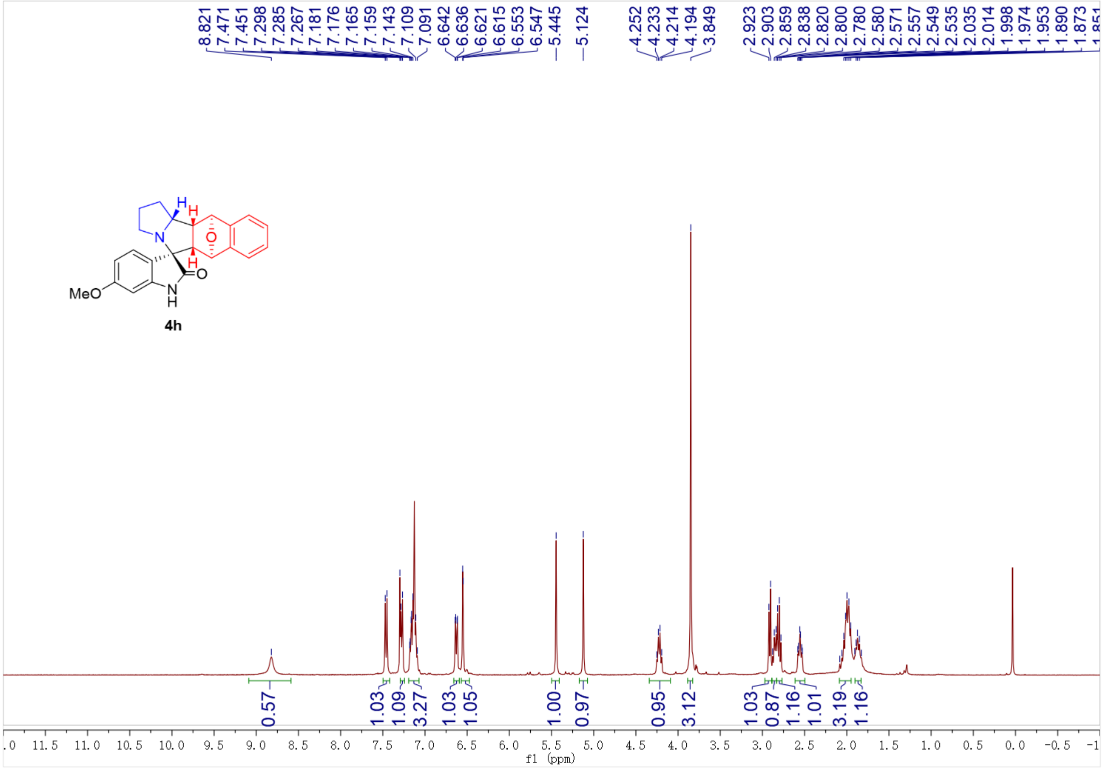


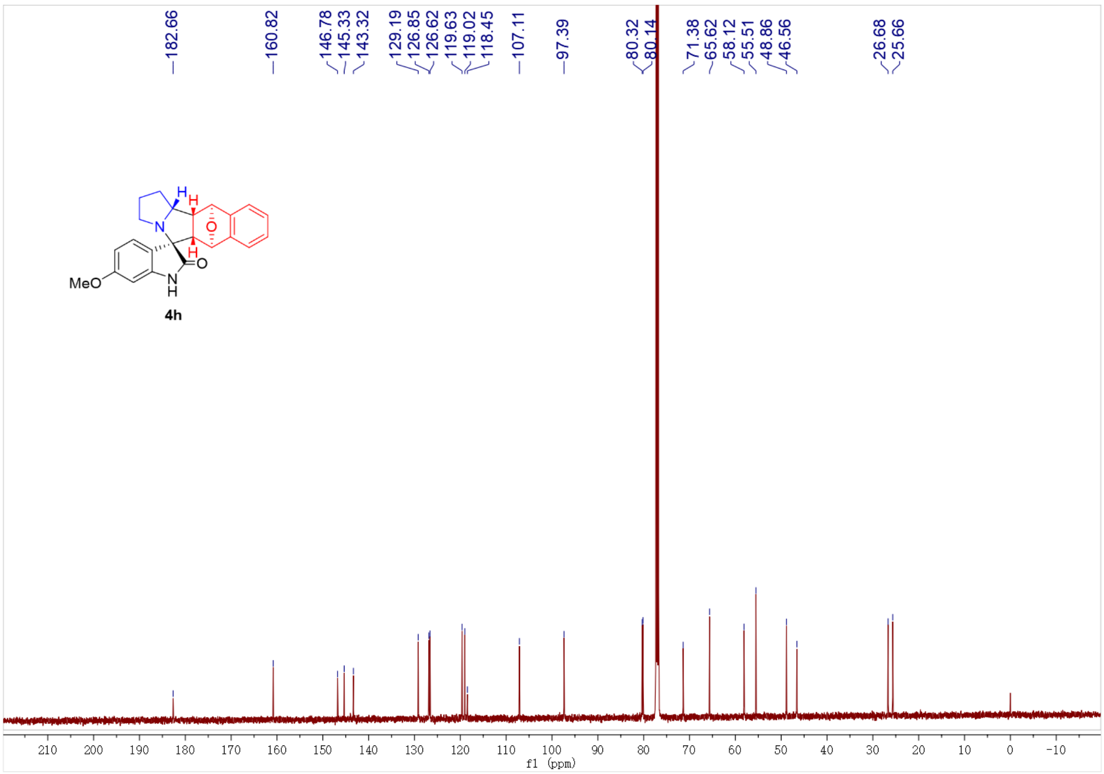


**$^{1}$H NMR** (400 MHz, $CDCl_3$), **$^{13}$C NMR** (125 MHz, $CDCl_3$) spectrum of **4h**

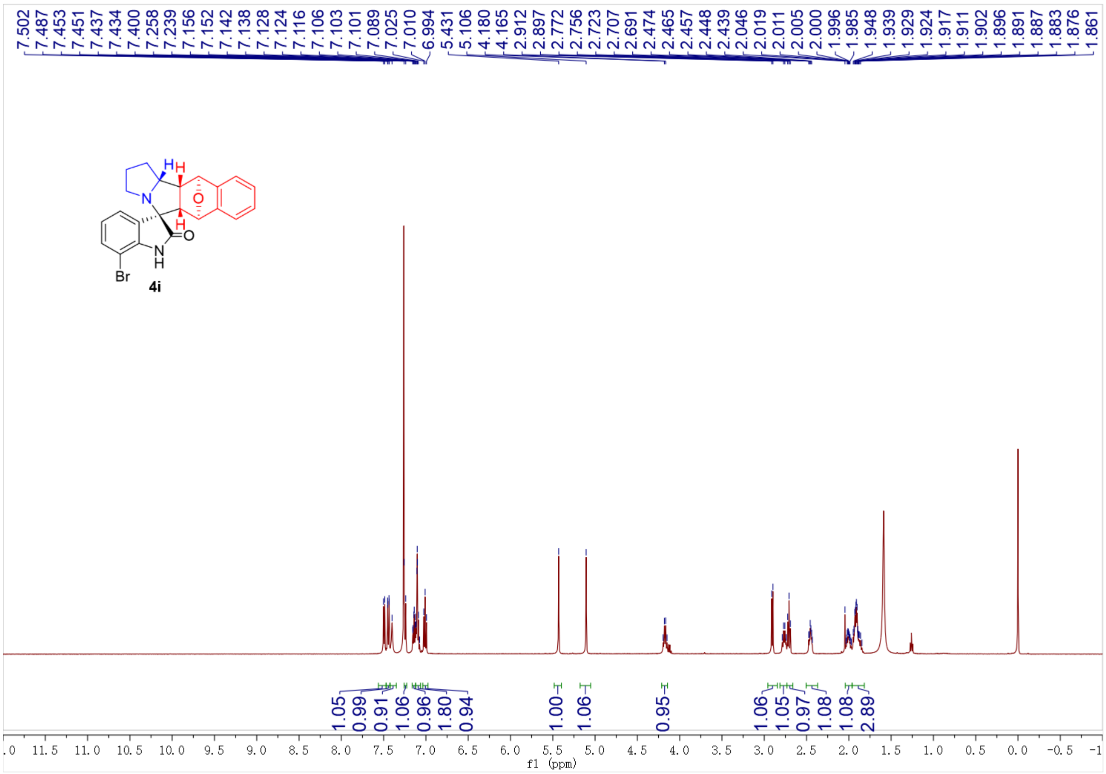


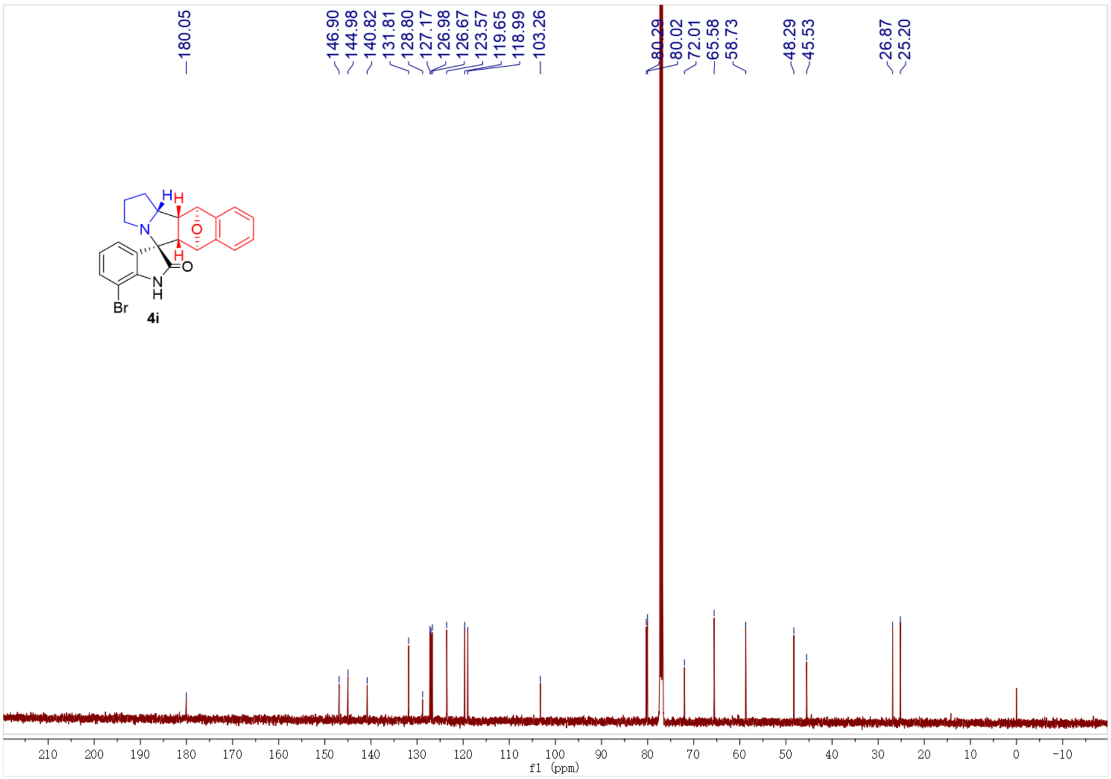


**¹H NMR** (500 MHz, CDCl$_3$), **¹³C NMR** (125 MHz, CDCl$_3$) spectrum of **4i**

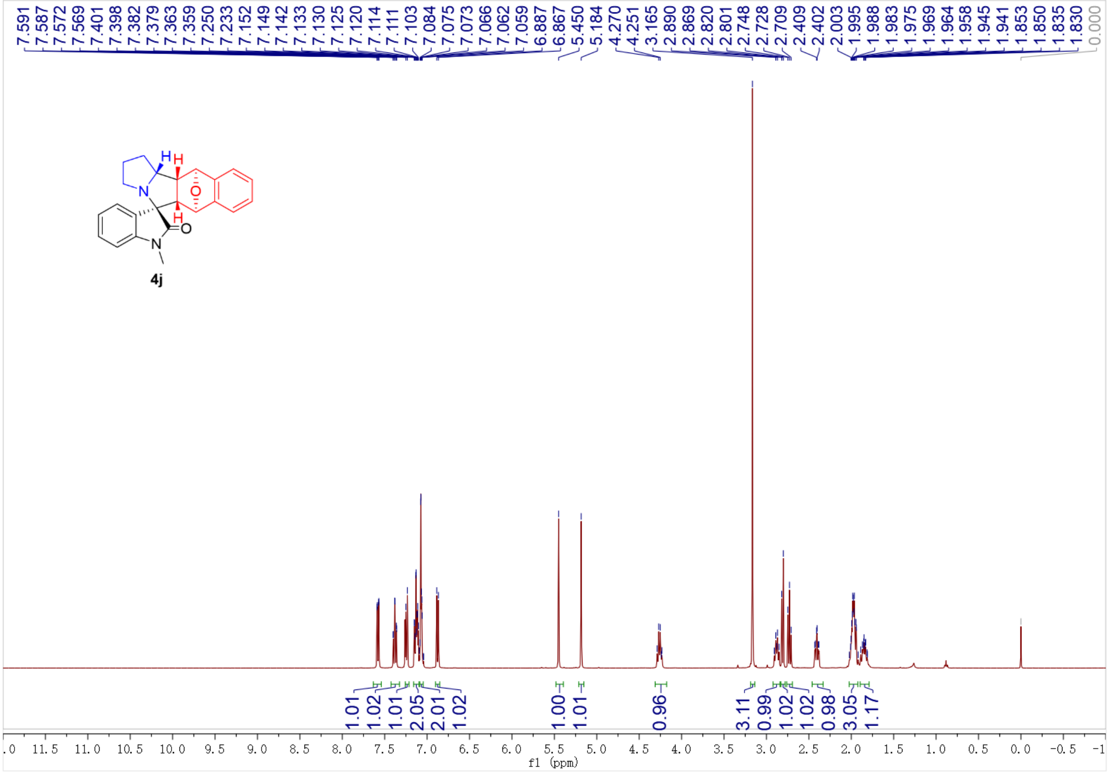


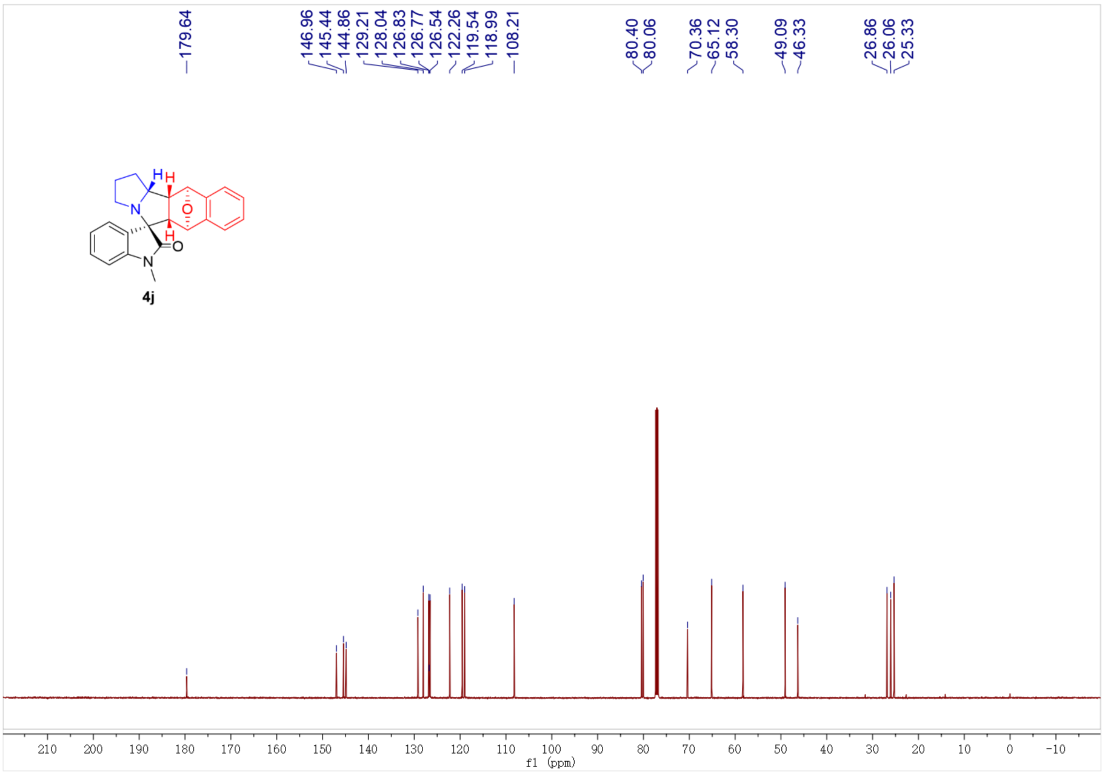


**$^{1}$H NMR** (400 MHz, $CDCl_3$), **$^{13}$C NMR** (125 MHz, $CDCl_3$) spectrum of **4j**

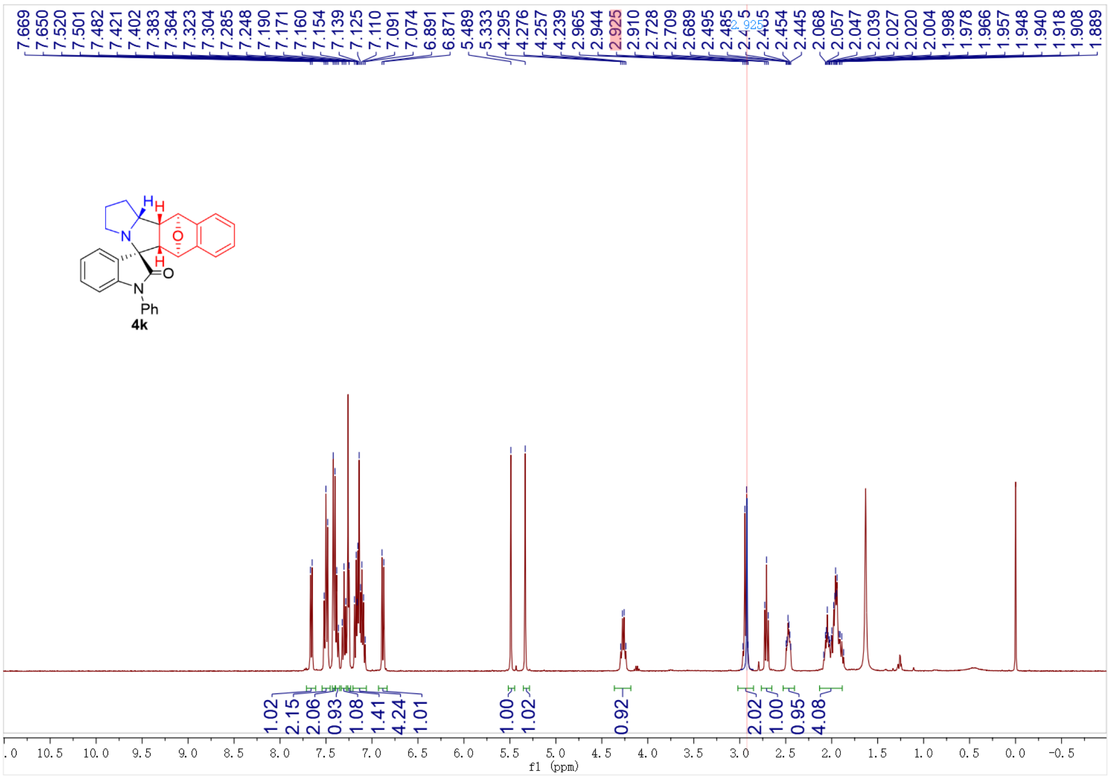

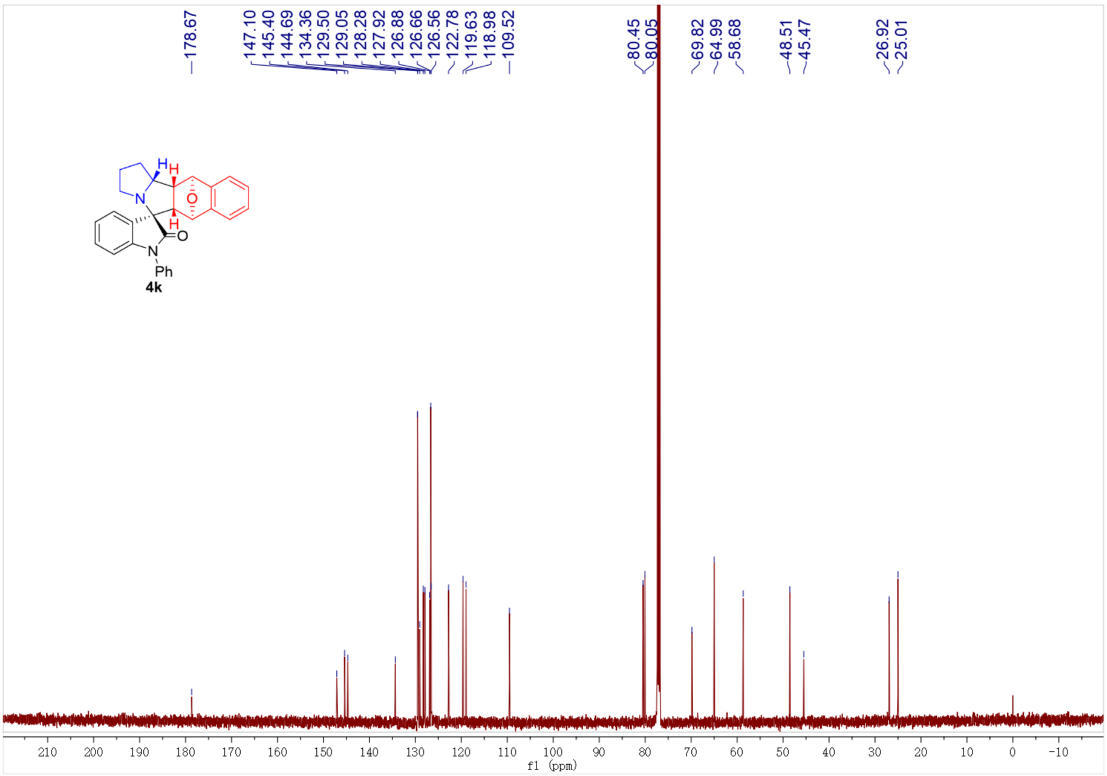

**$^{1}$H NMR** (400 MHz, $CDCl_3$), **$^{13}$C NMR** (125 MHz, $CDCl_3$) spectrum of **4k**

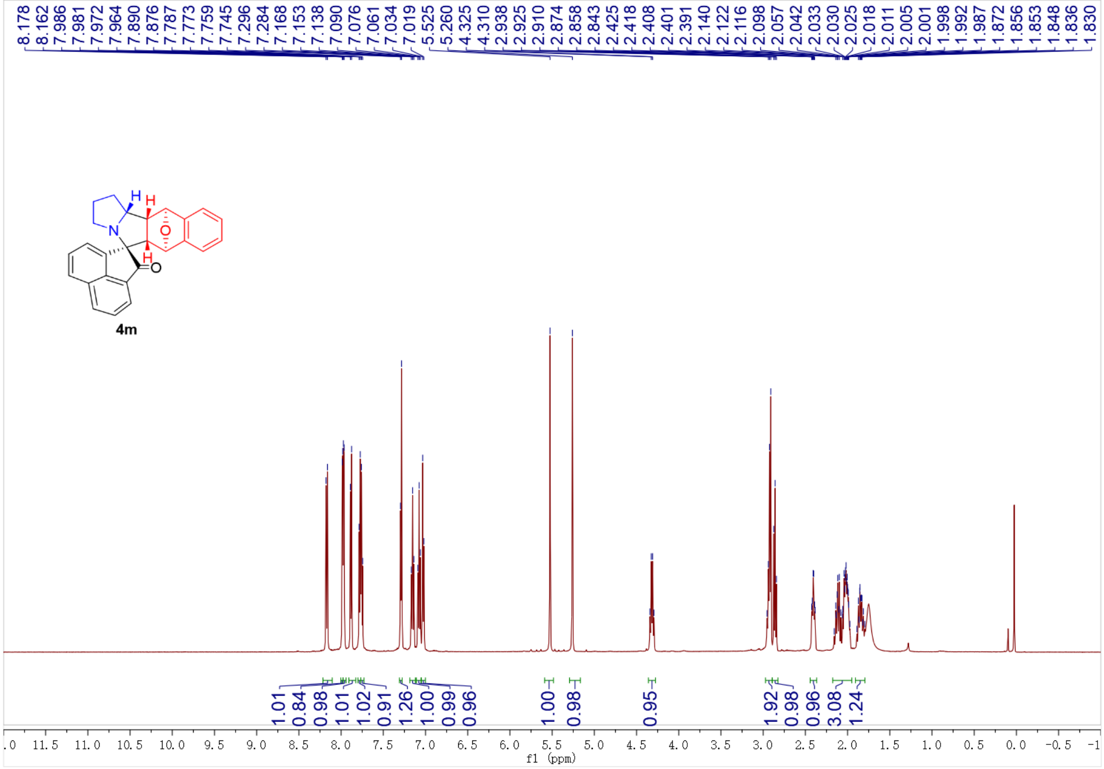

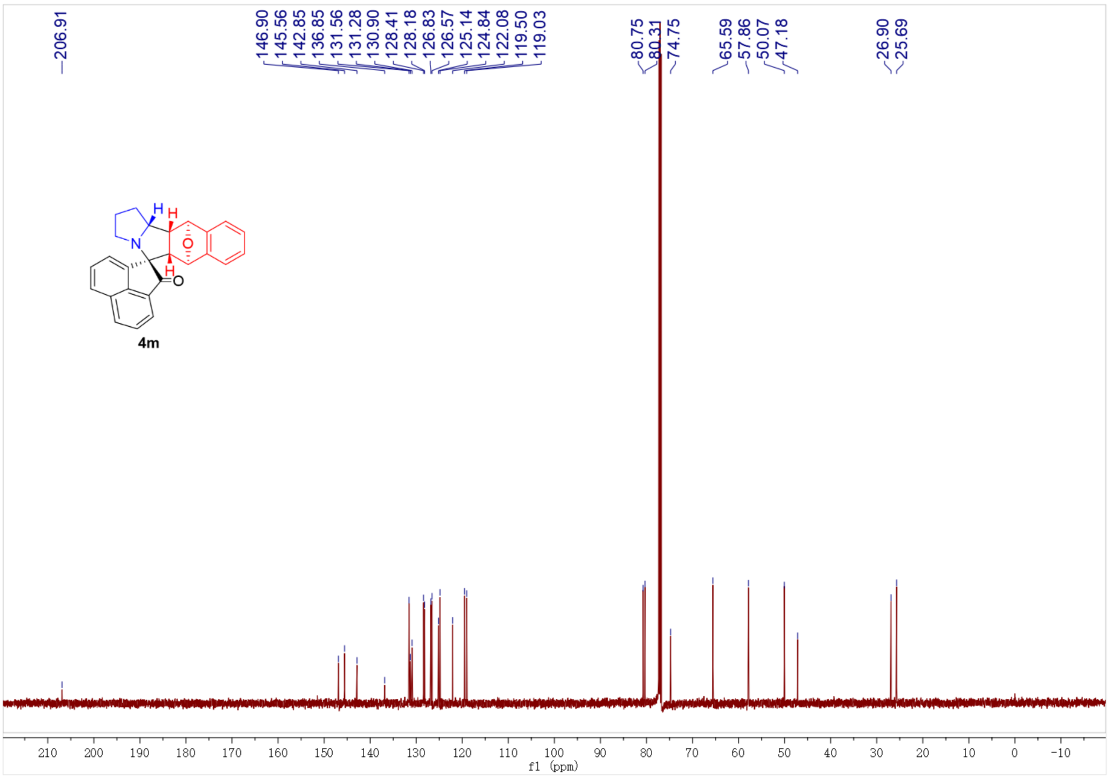


**$^{1}$H NMR** (500 MHz, $CDCl_3$), **$^{13}$C NMR** (125 MHz, $CDCl_3$) spectrum of **4m**

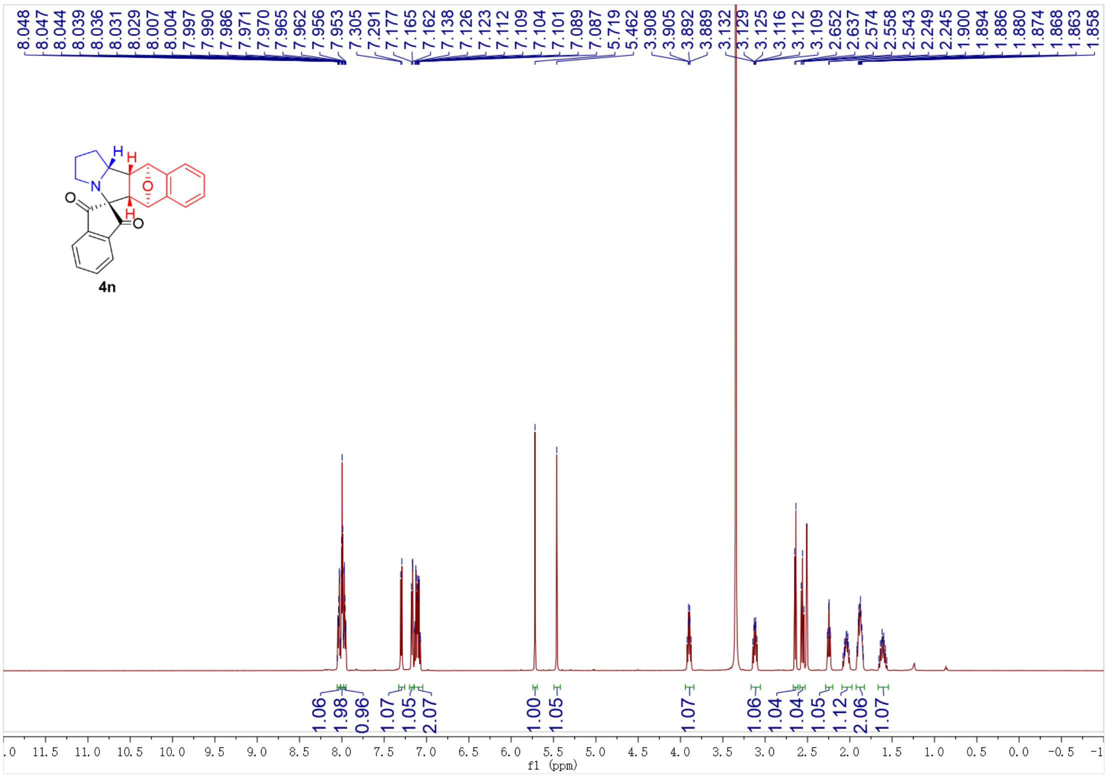

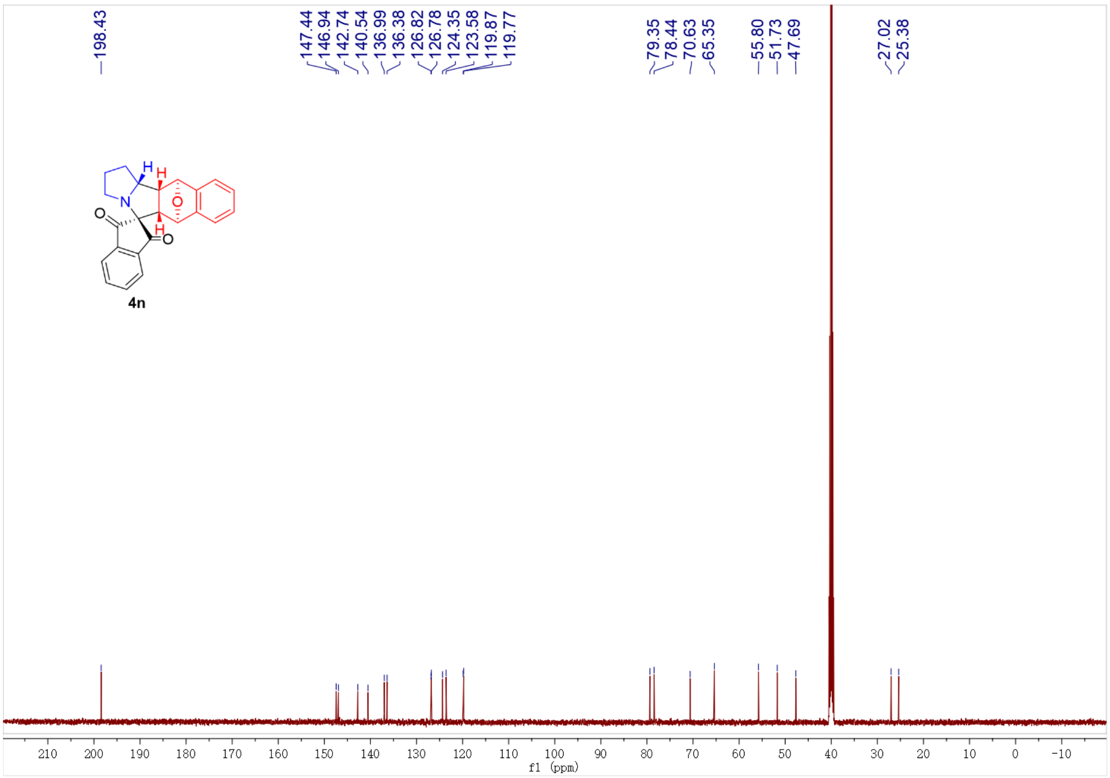

**$^{1}$H NMR** (500 MHz, DMSO-$d_6$), **$^{13}$C NMR** (125 MHz, DMSO-$d_6$) spectrum of **4n**

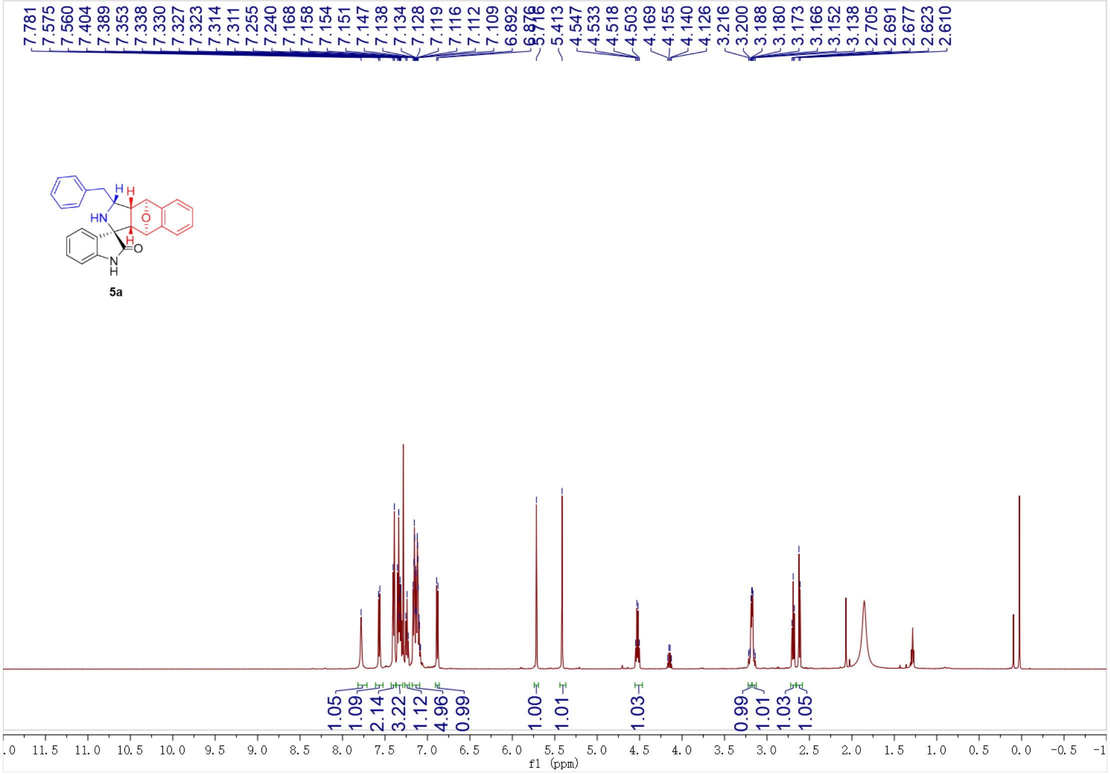

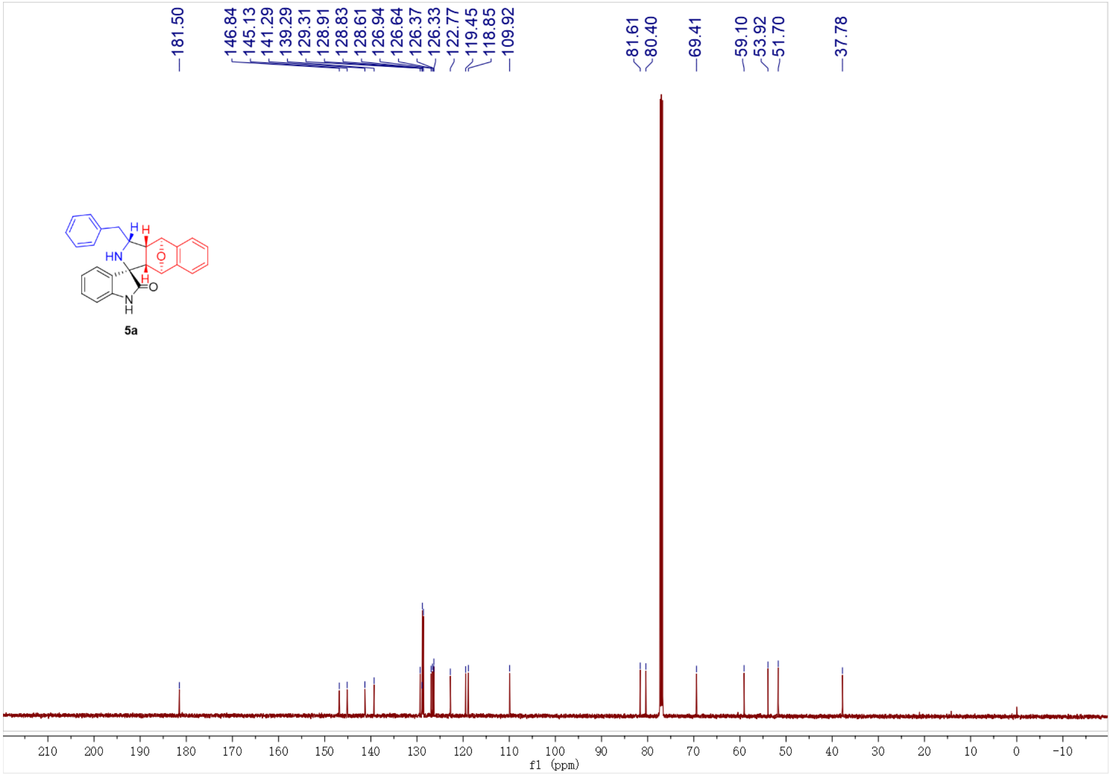

$^{1}$**H NMR** (500 MHz, $CDCl_3$), $^{13}$**C NMR** (125 MHz, $CDCl_3$) spectrum of **5a**

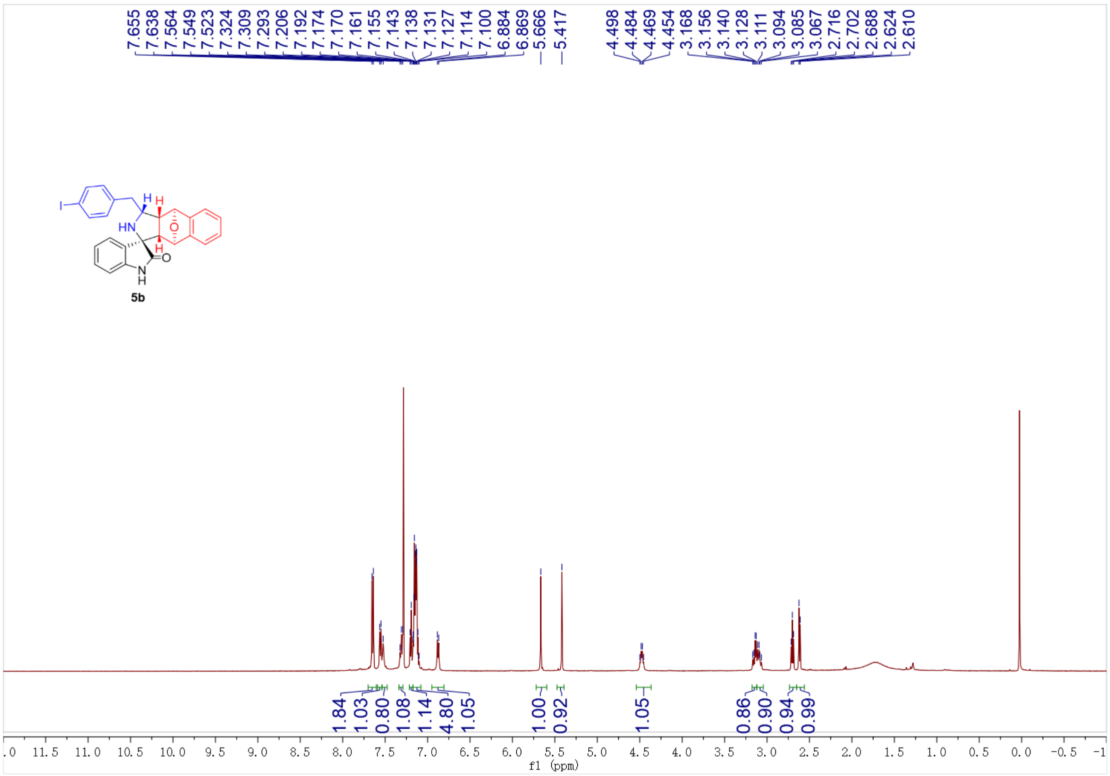


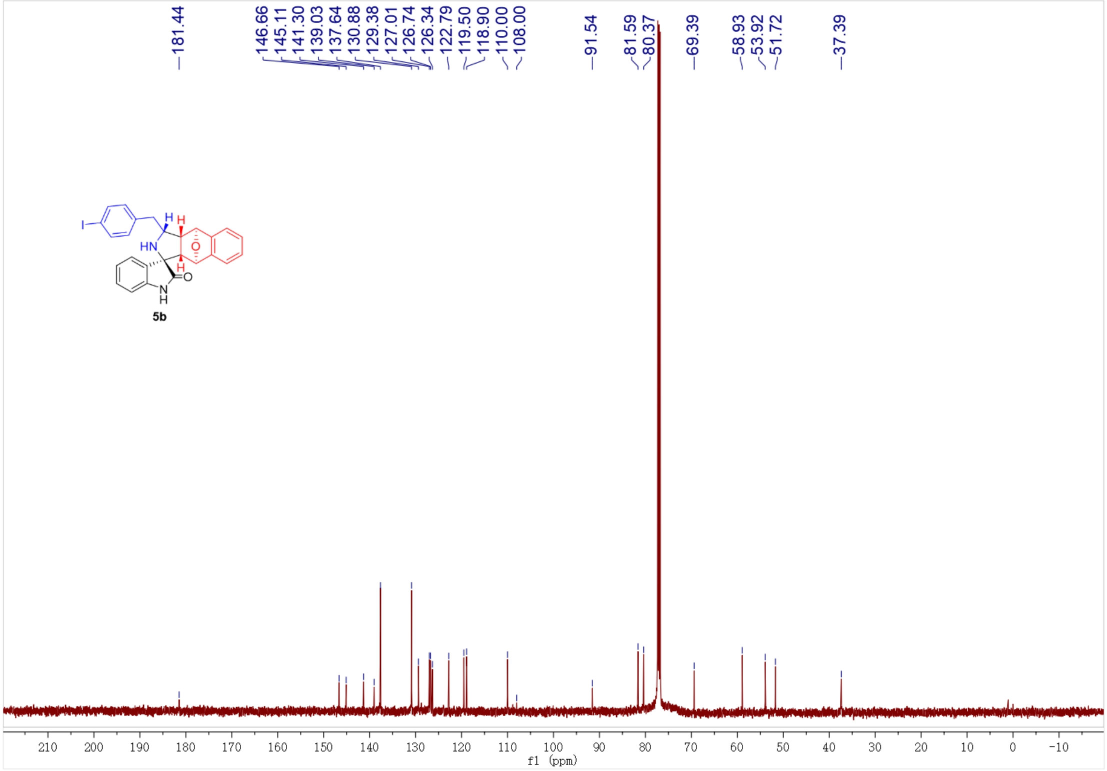


**$^{1}$H NMR** (500 MHz, $CDCl_3$), **$^{13}$C NMR** (125 MHz, $CDCl_3$) spectrum of **5b**

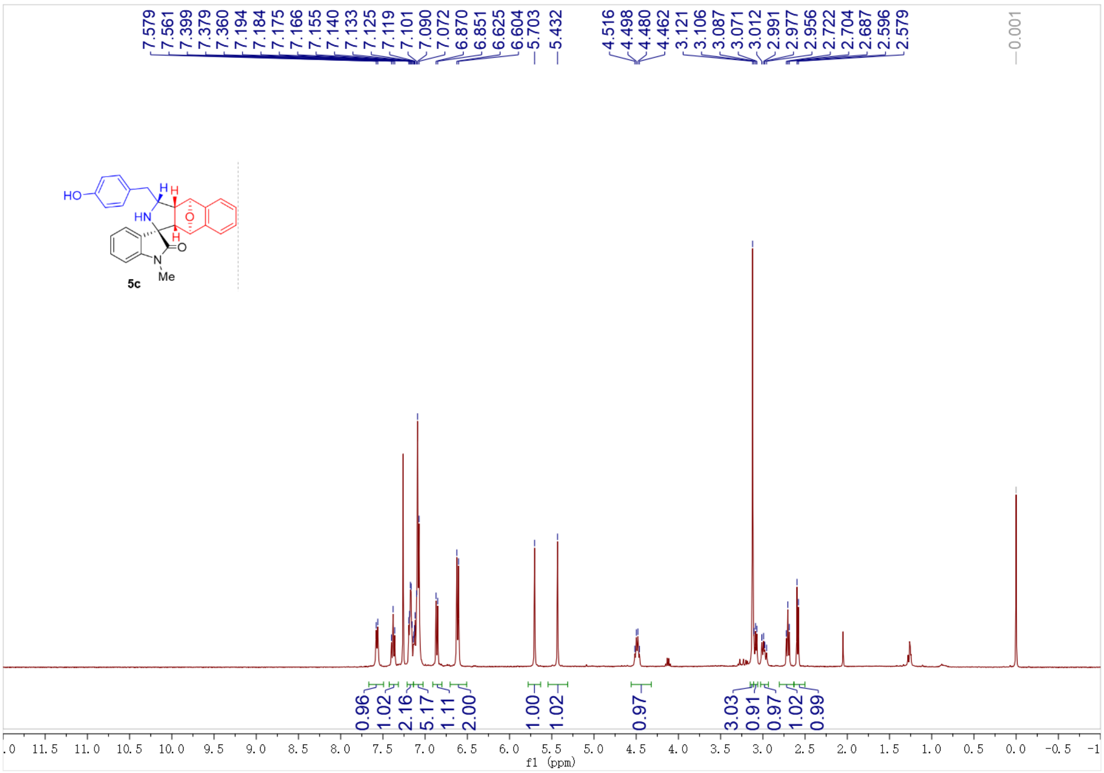

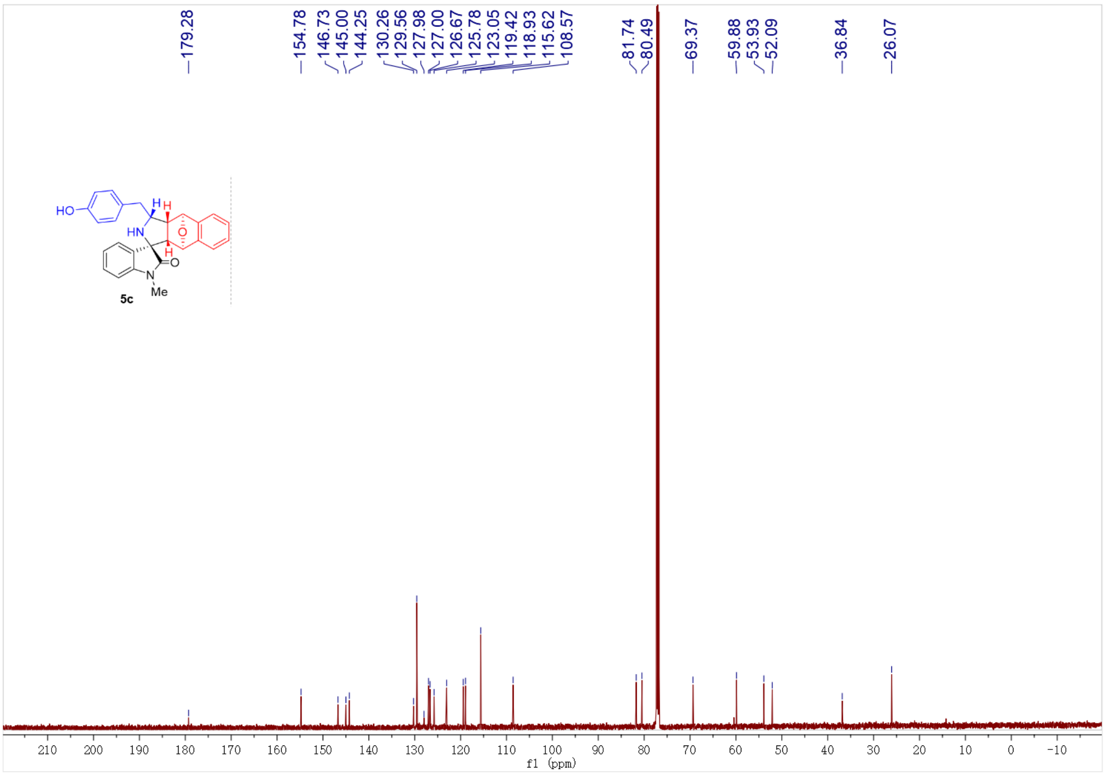

**$^{1}$H NMR** (400 MHz, $CDCl_3$), **$^{13}$C NMR** (125 MHz, $CDCl_3$) spectrum of **5c**

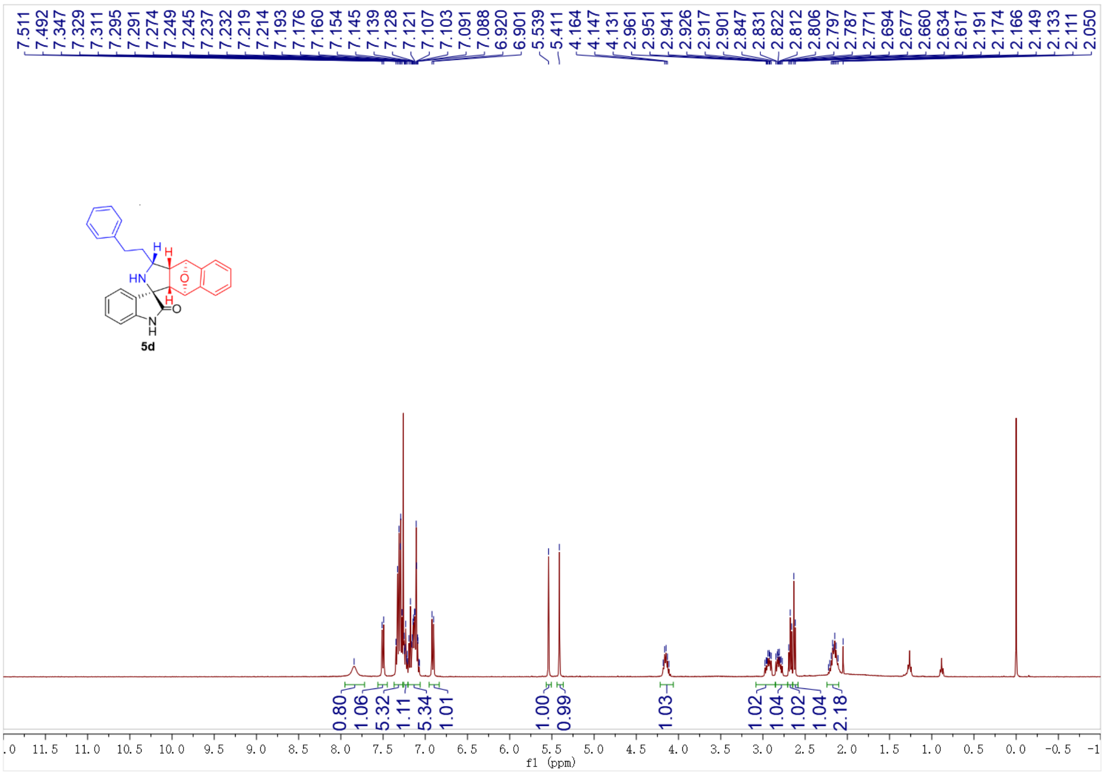


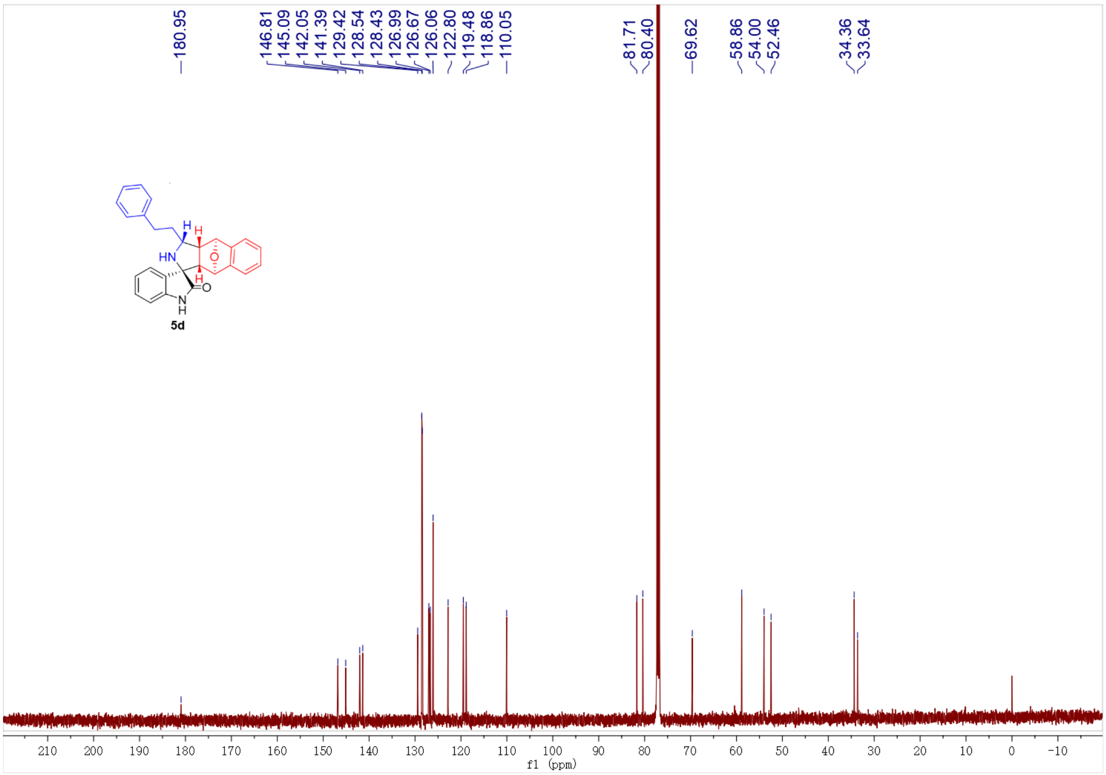


**$^{1}$H NMR** (400 MHz, $CDCl_3$), **$^{13}$C NMR** (125 MHz, $CDCl_3$) spectrum of **5d**

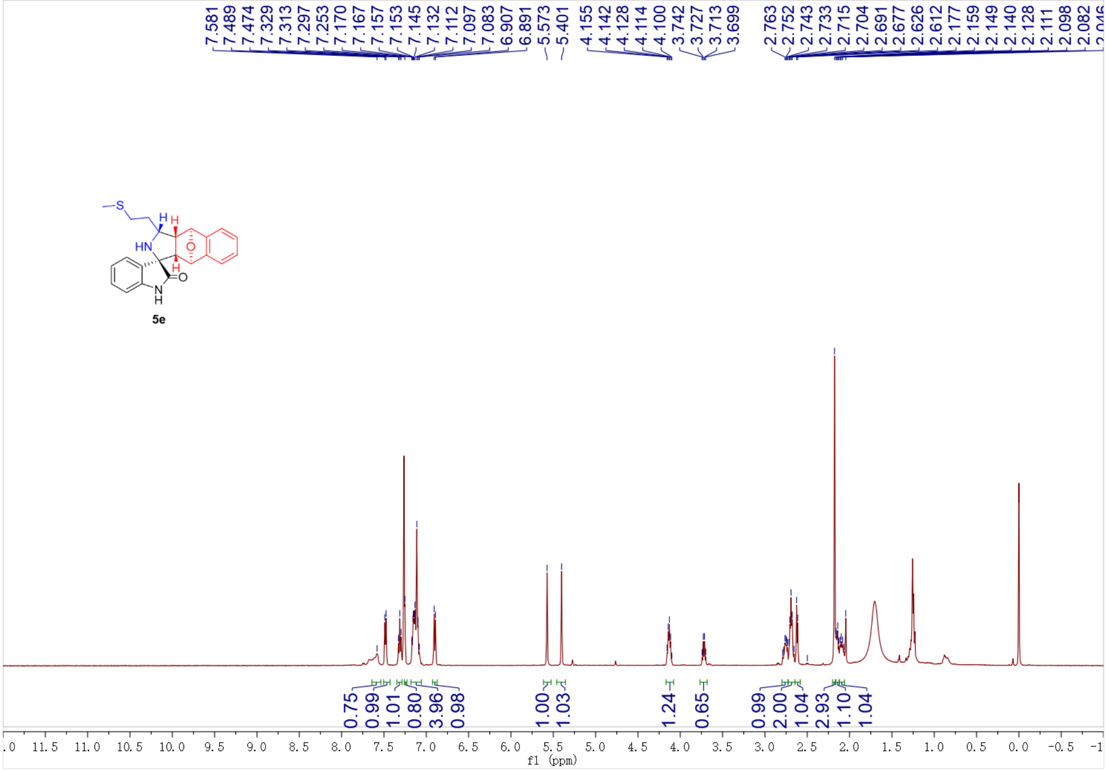


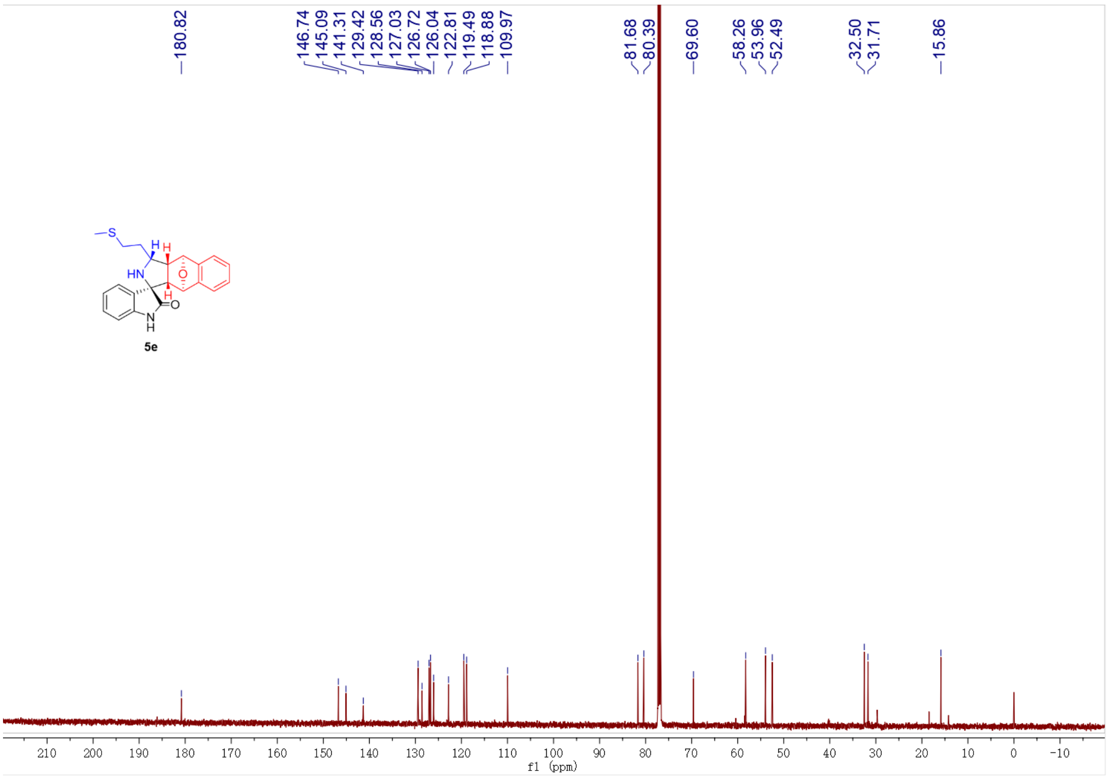


**$^1$H NMR** (500 MHz, $CDCl_3$), **$^{13}$C NMR** (125 MHz, $CDCl_3$) spectrum of **5e**

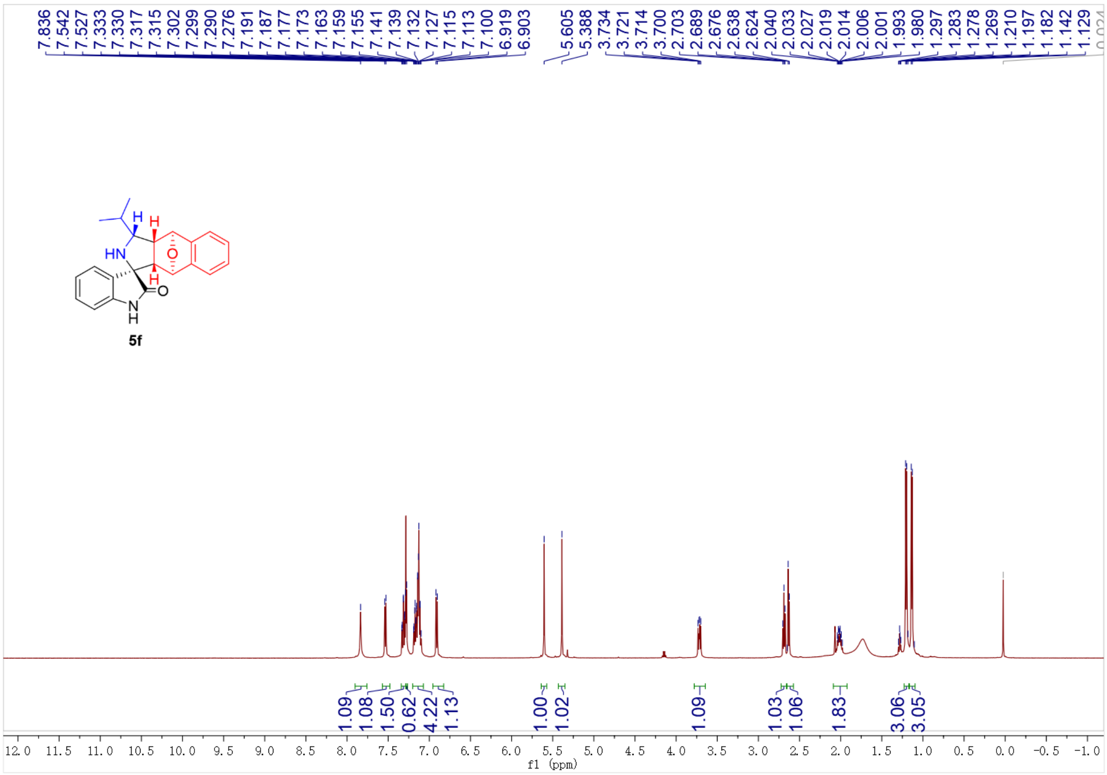

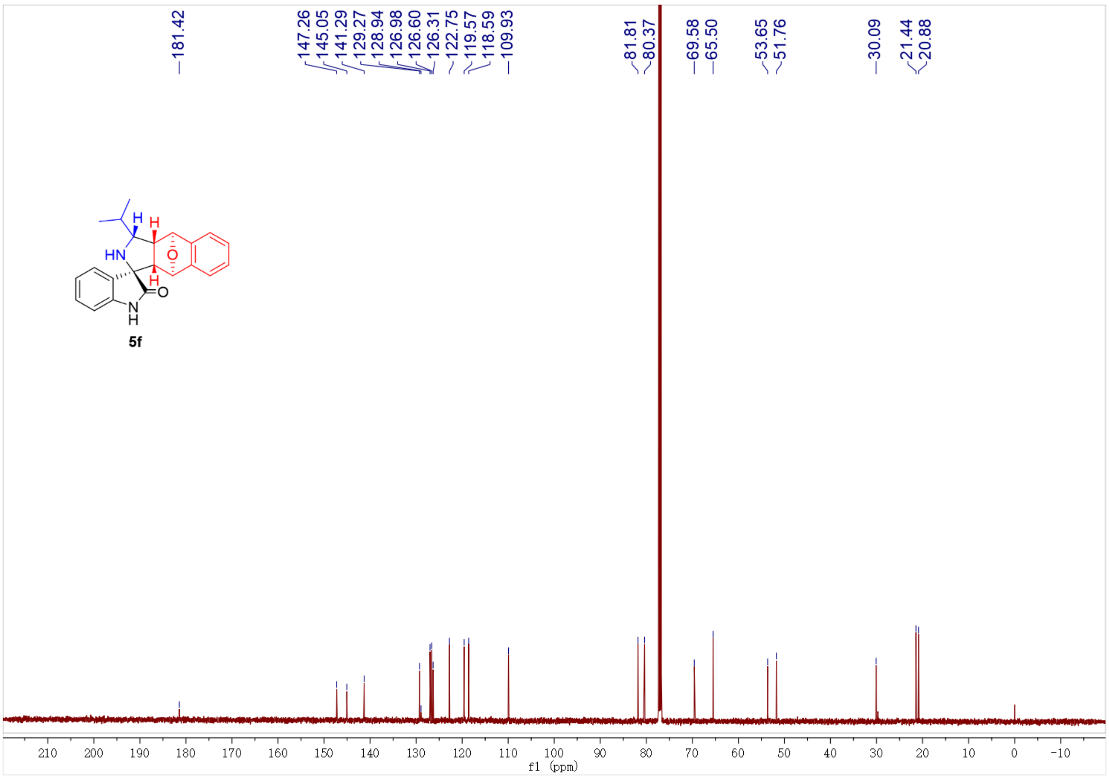

**$^{1}$H NMR** (500 MHz, $CDCl_3$), **$^{13}$C NMR** (125 MHz, $CDCl_3$) spectrum of **5f**

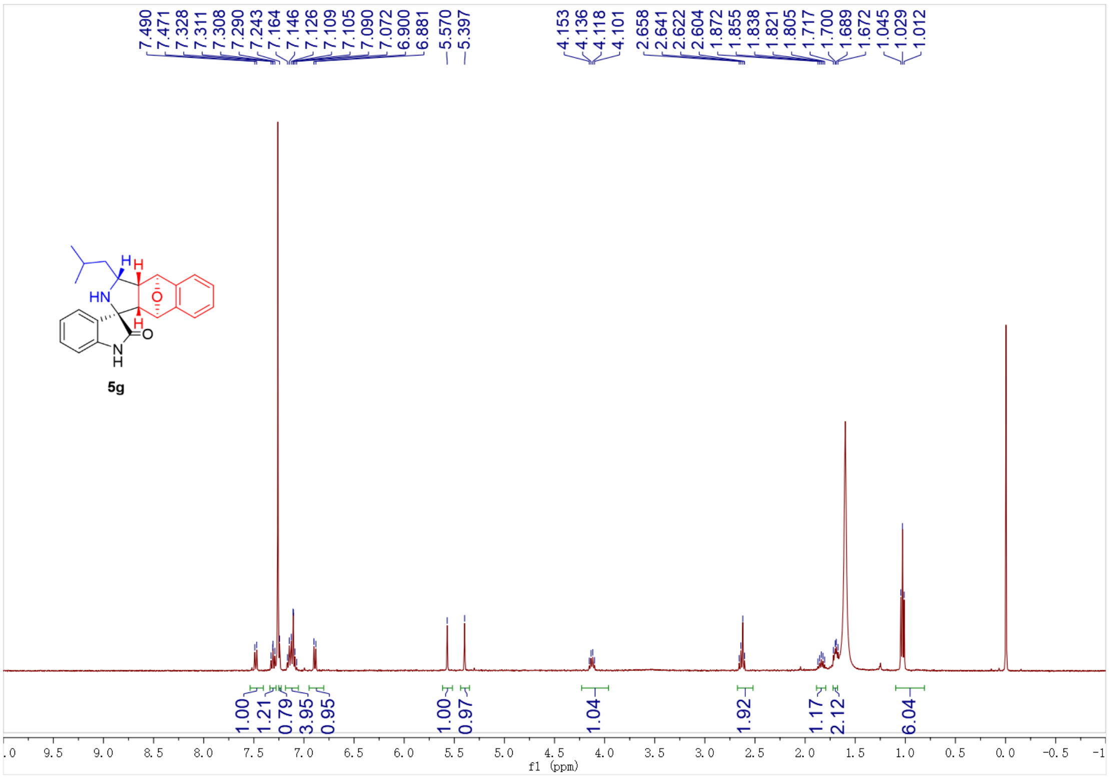


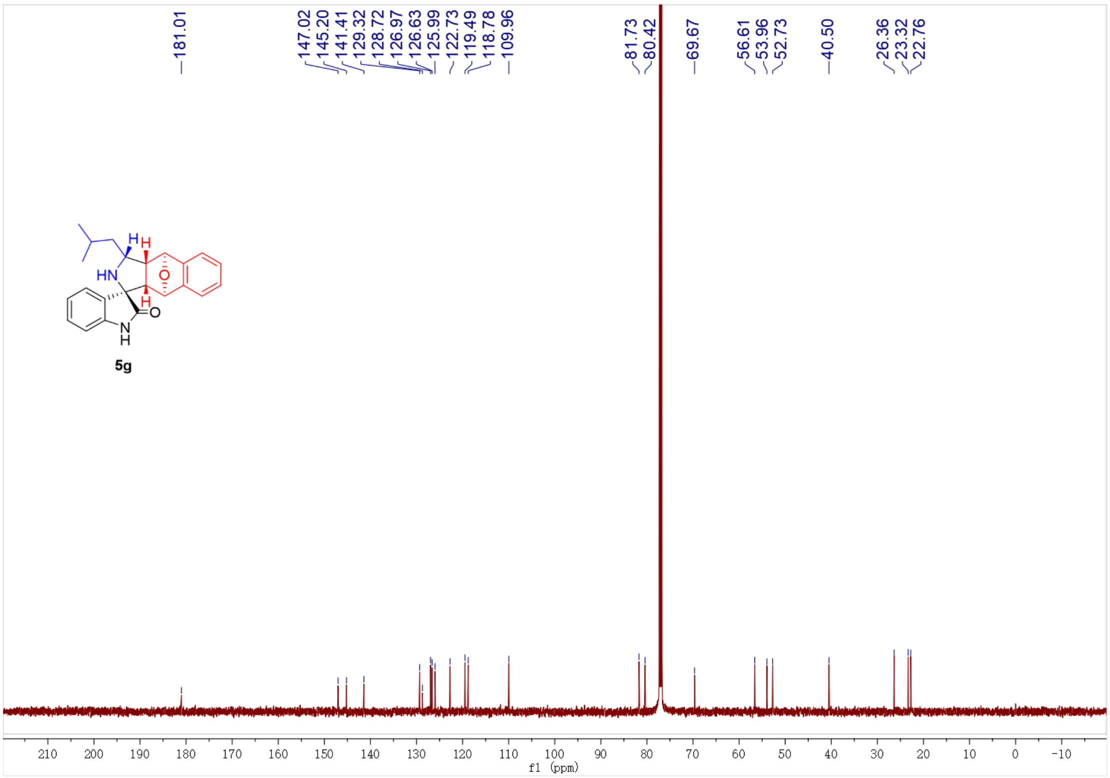


**$^{1}$H NMR** (400 MHz, $CDCl_3$), **$^{13}$C NMR** (125 MHz, $CDCl_3$) spectrum of **5g**

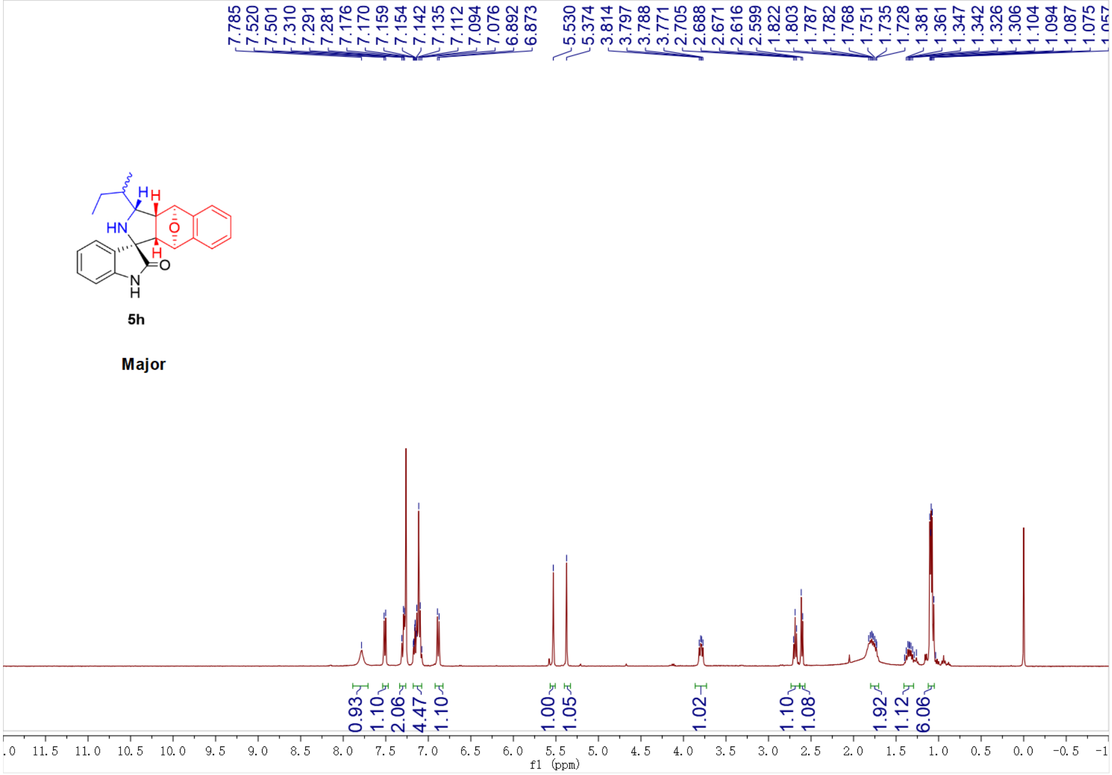

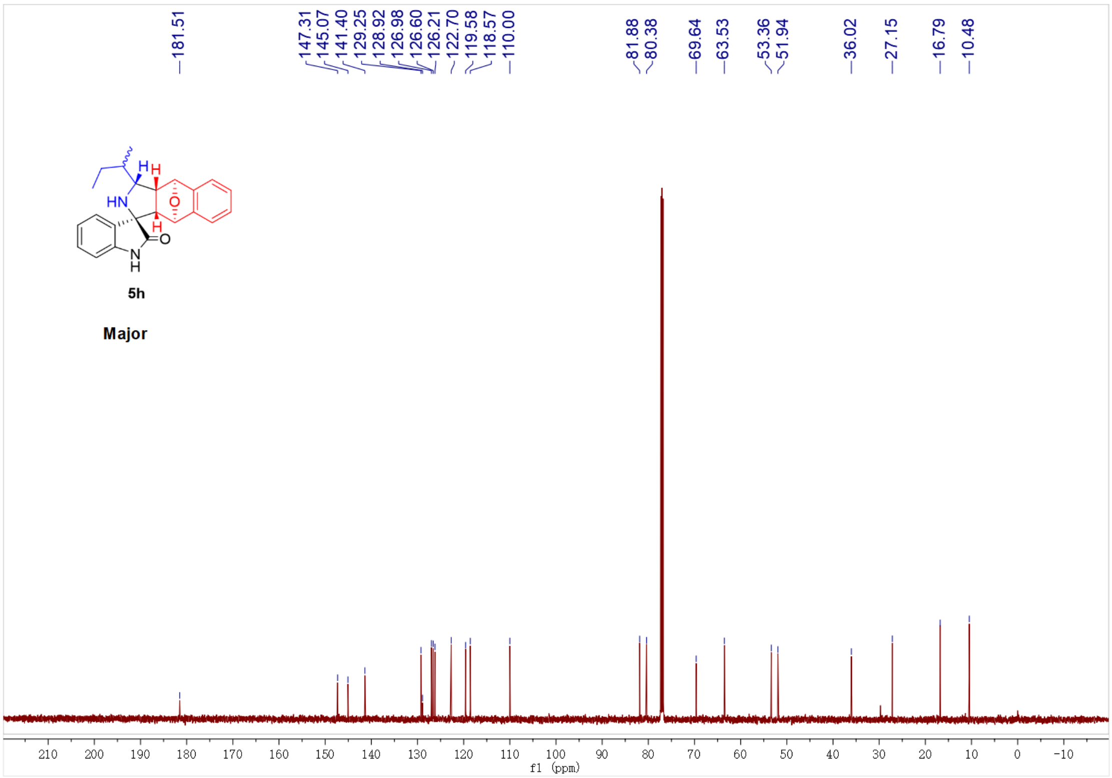

$^{1}$**H NMR** (400 MHz, $CDCl_3$), $^{13}$**C NMR** (125 MHz, $CDCl_3$) spectrum of **5h**

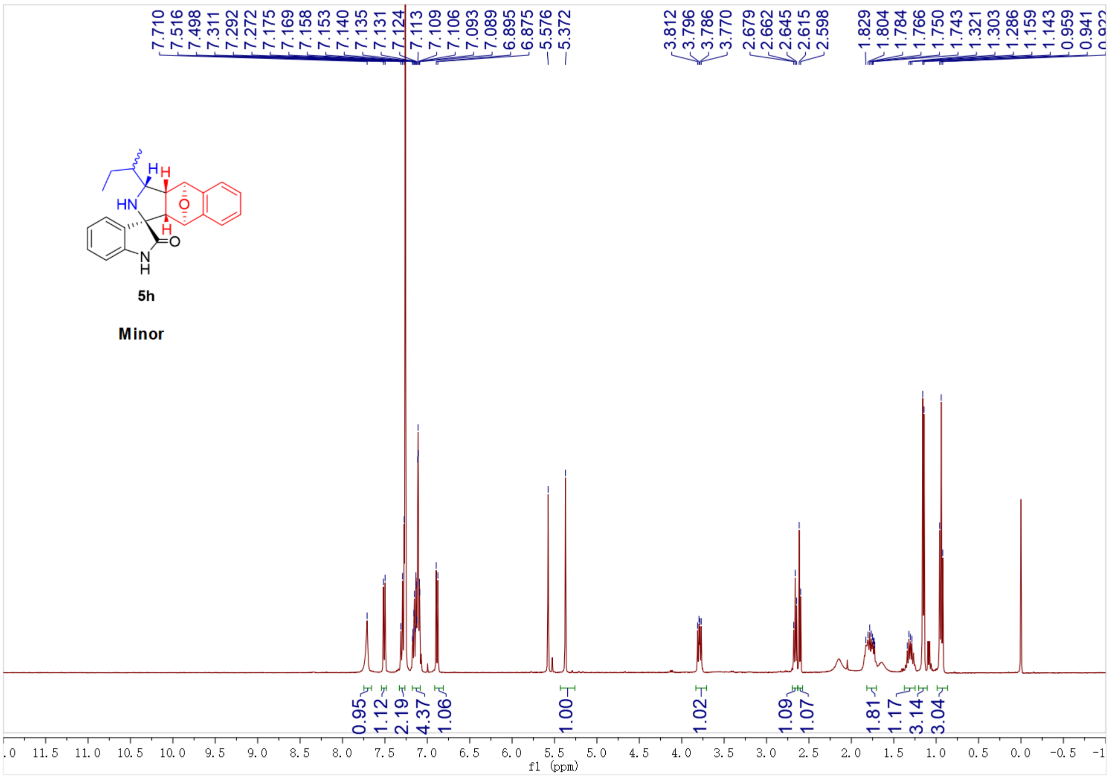


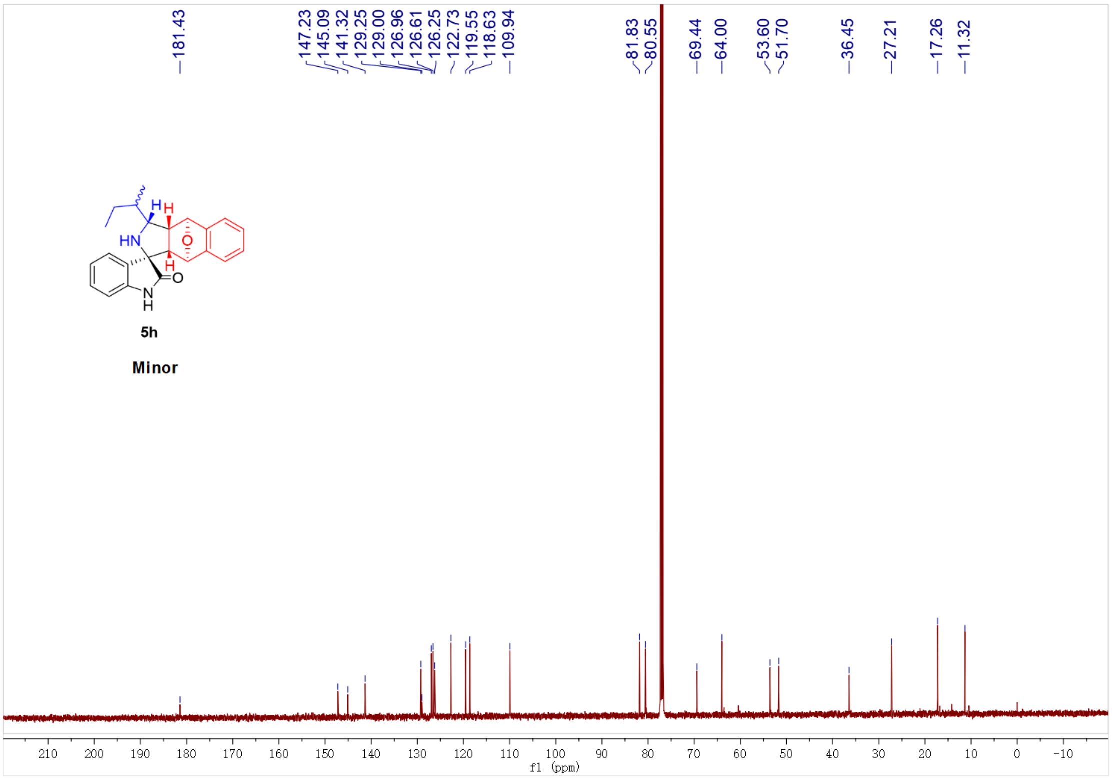


**$^{1}$H NMR** (400 MHz, $CDCl_3$), **$^{13}$C NMR** (125 MHz, $CDCl_3$) spectrum of **5h**

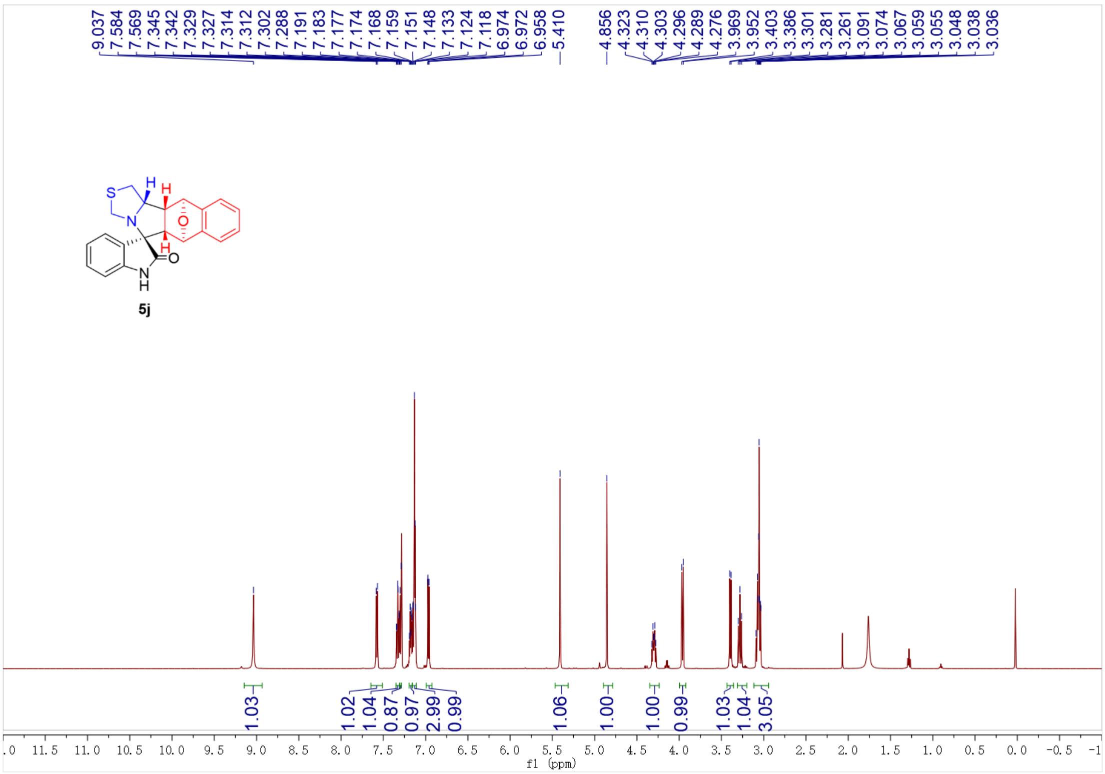


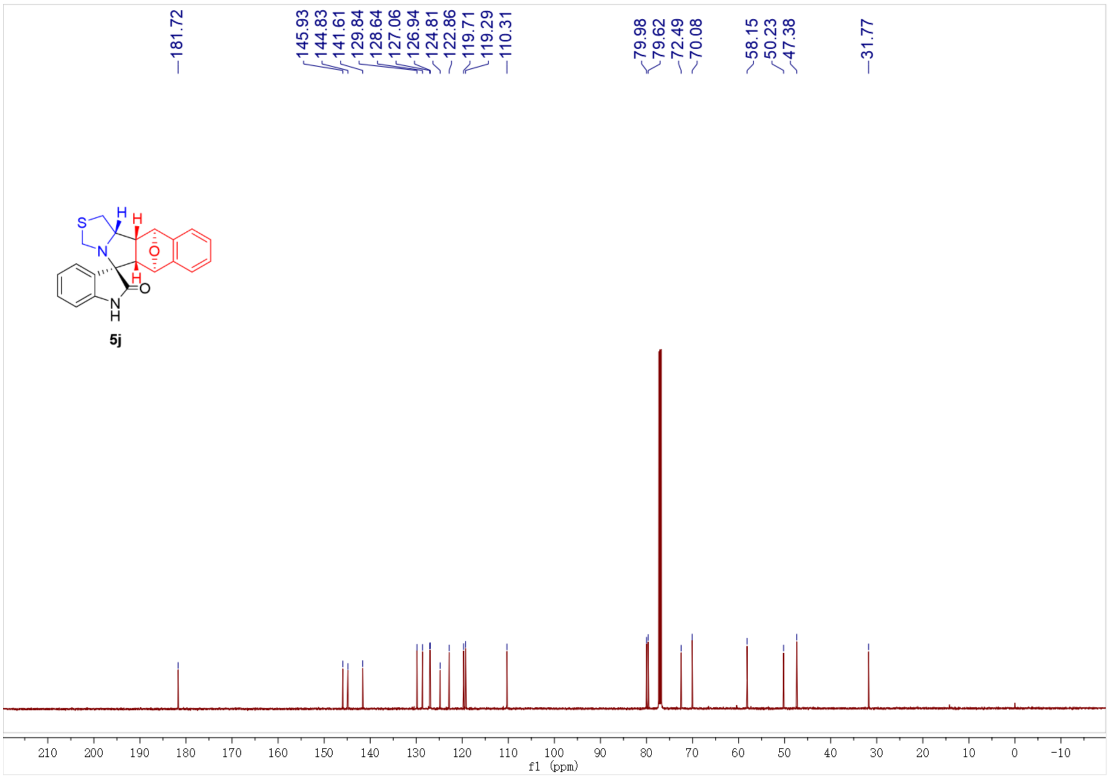


**$^{1}$H NMR** (500 MHz, $CDCl_3$), **$^{13}$C NMR** (125 MHz, $CDCl_3$) spectrum of **5j**

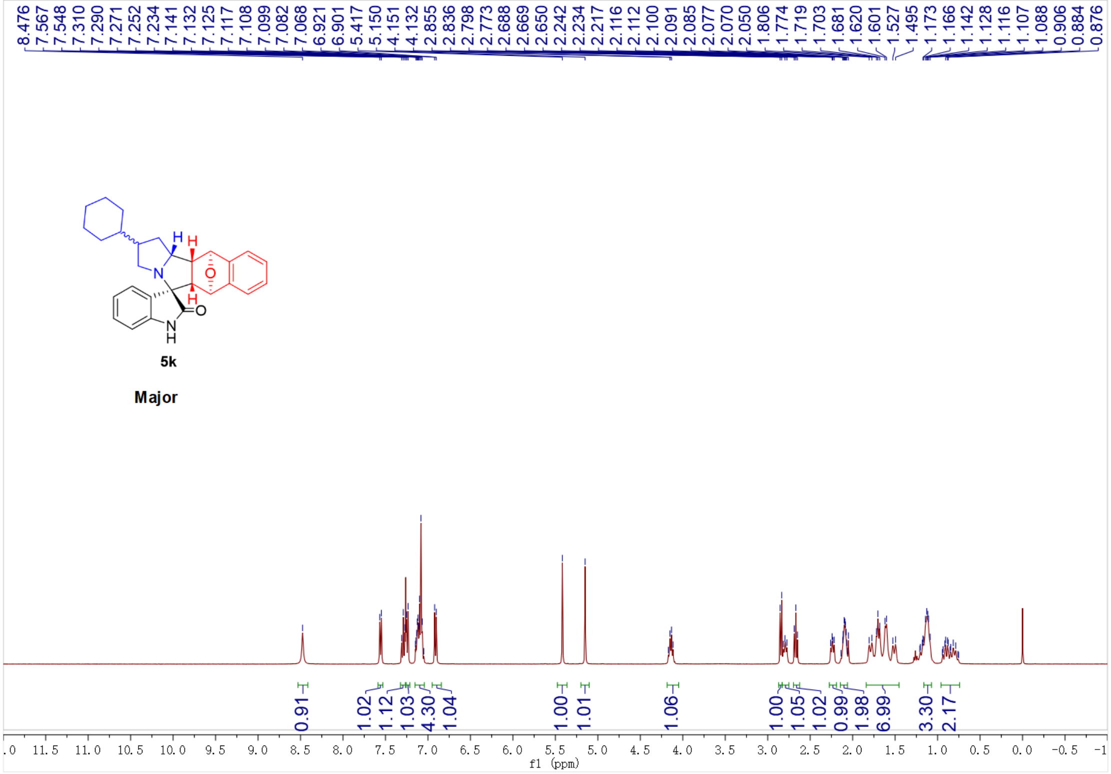

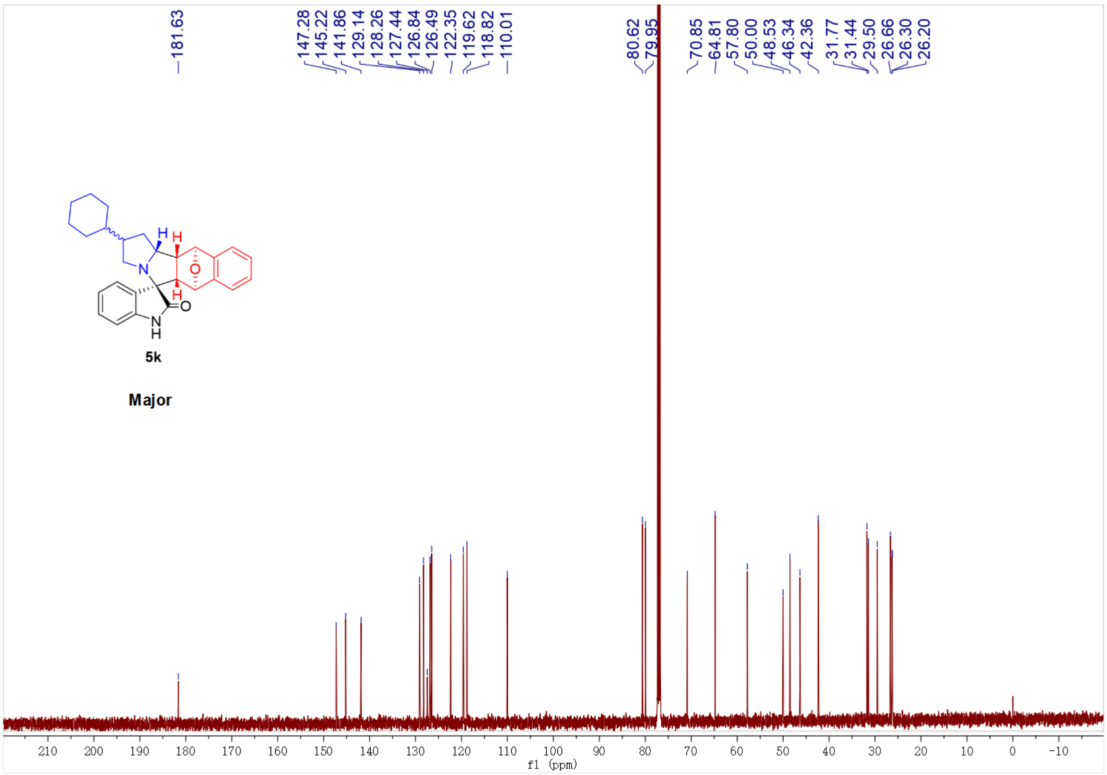

$^{1}$**H NMR** (400 MHz, $CDCl_3$), $^{13}$**C NMR** (125 MHz, $CDCl_3$) spectrum of **5k**

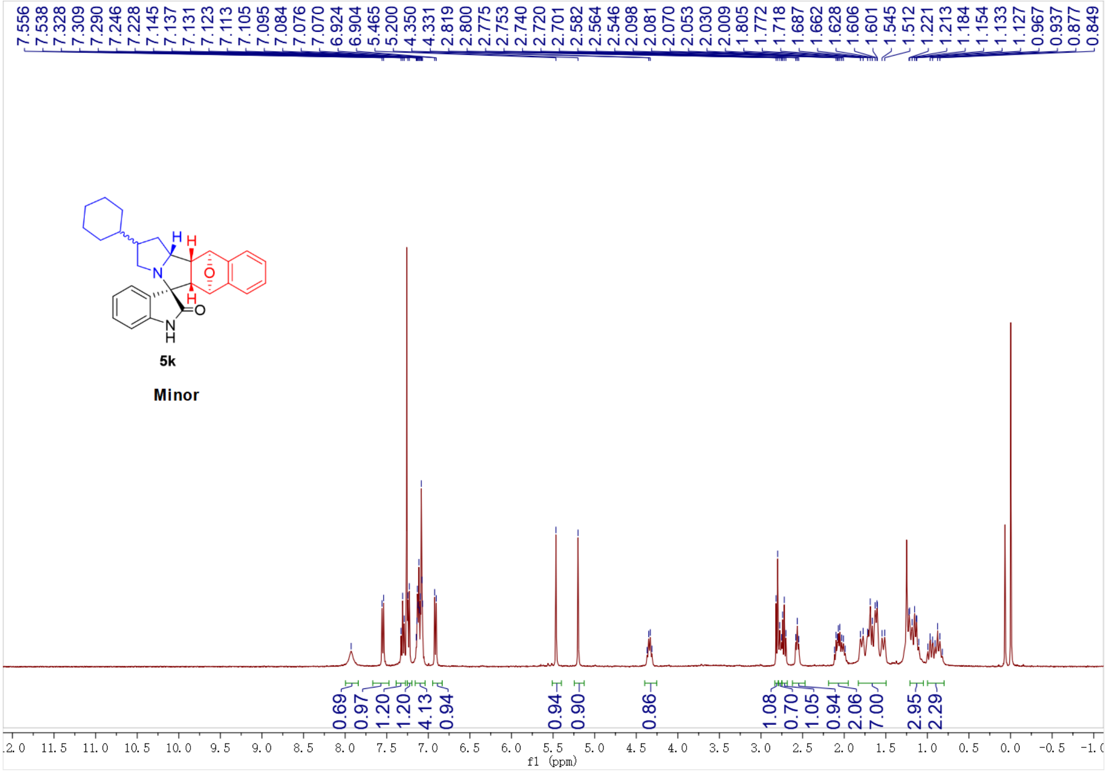


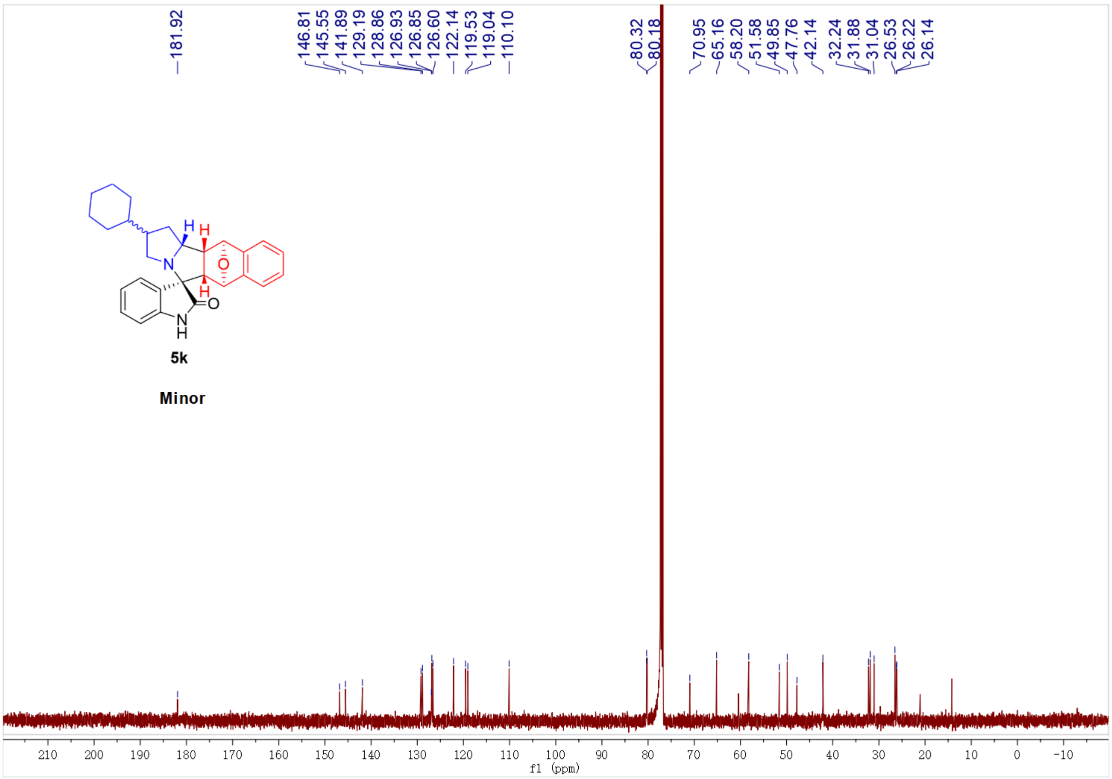


**$^{1}$H NMR** (400 MHz, $CDCl_3$), **$^{13}$C NMR** (125 MHz, $CDCl_3$) spectrum of **5k**

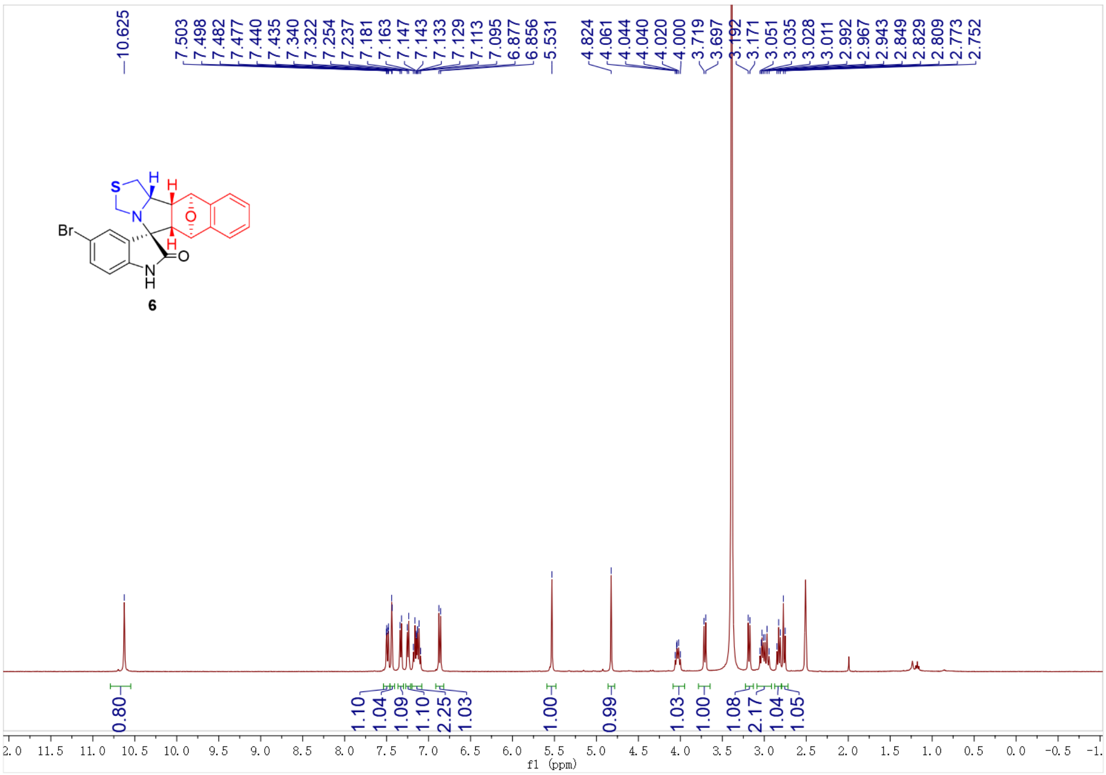

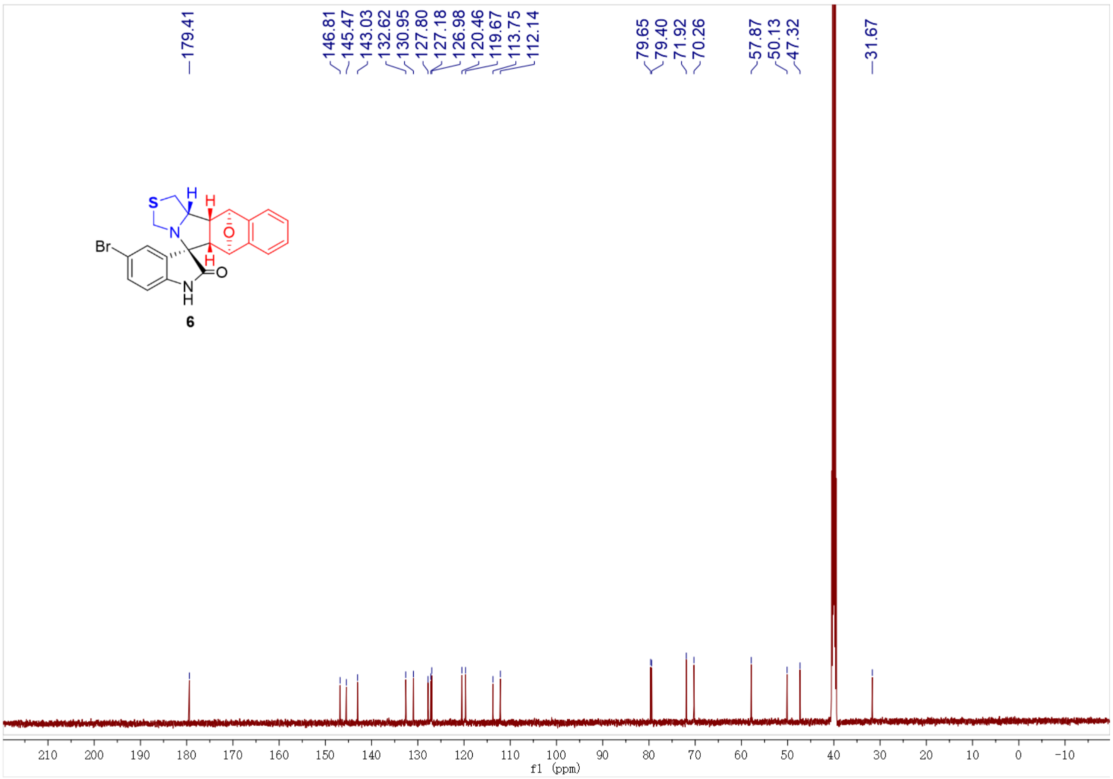

$^{1}$**H NMR** (400 MHz, DMSO-$d_6$), $^{13}$**C NMR** (125 MHz, DMSO-$d_6$) spectrum of **6**

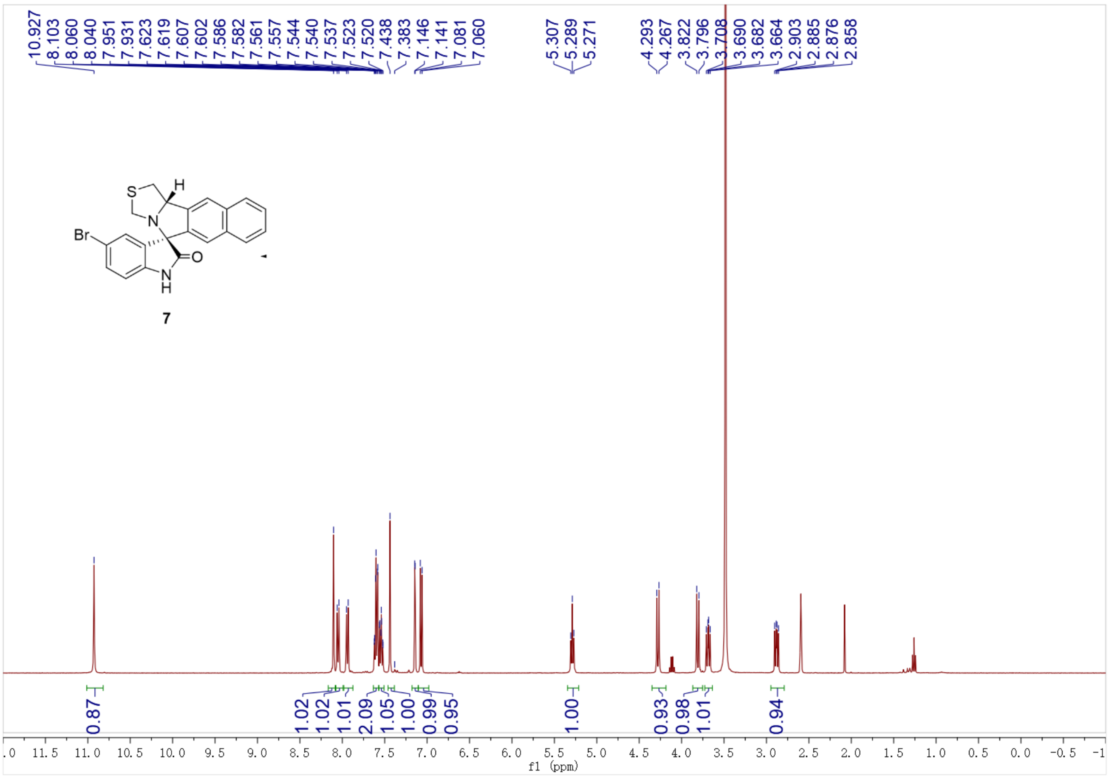

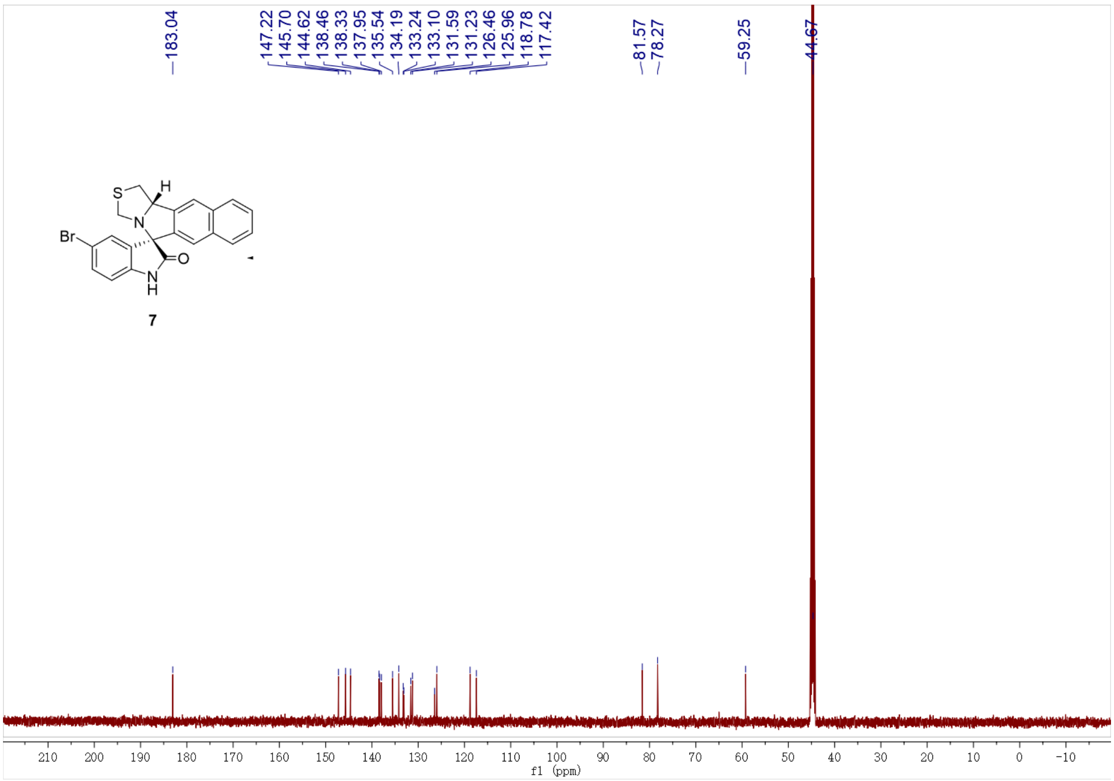

**$^{1}$H NMR** (400 MHz, DMSO-$d_6$), **$^{13}$C NMR** (125 MHz, DMSO-$d_6$) spectrum of **7**

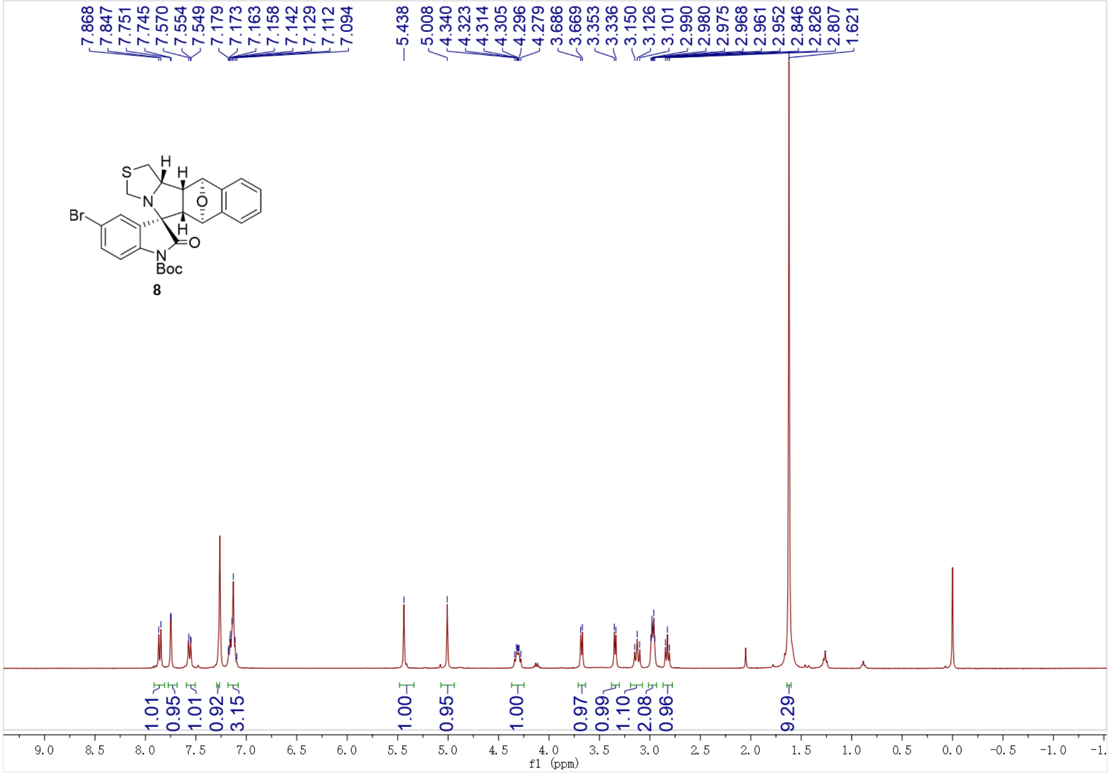


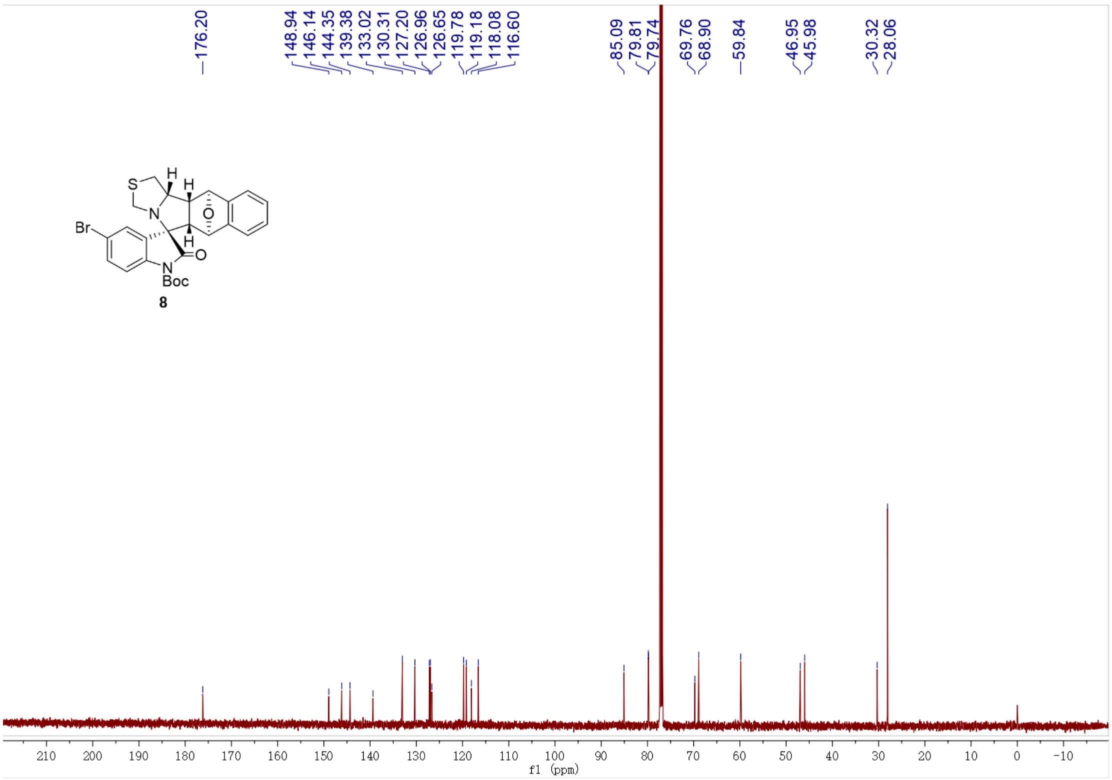


**$^1$H NMR** (400 MHz, $CDCl_3$), **$^{13}$C NMR** (125 MHz, $CDCl_3$) spectrum of **8**

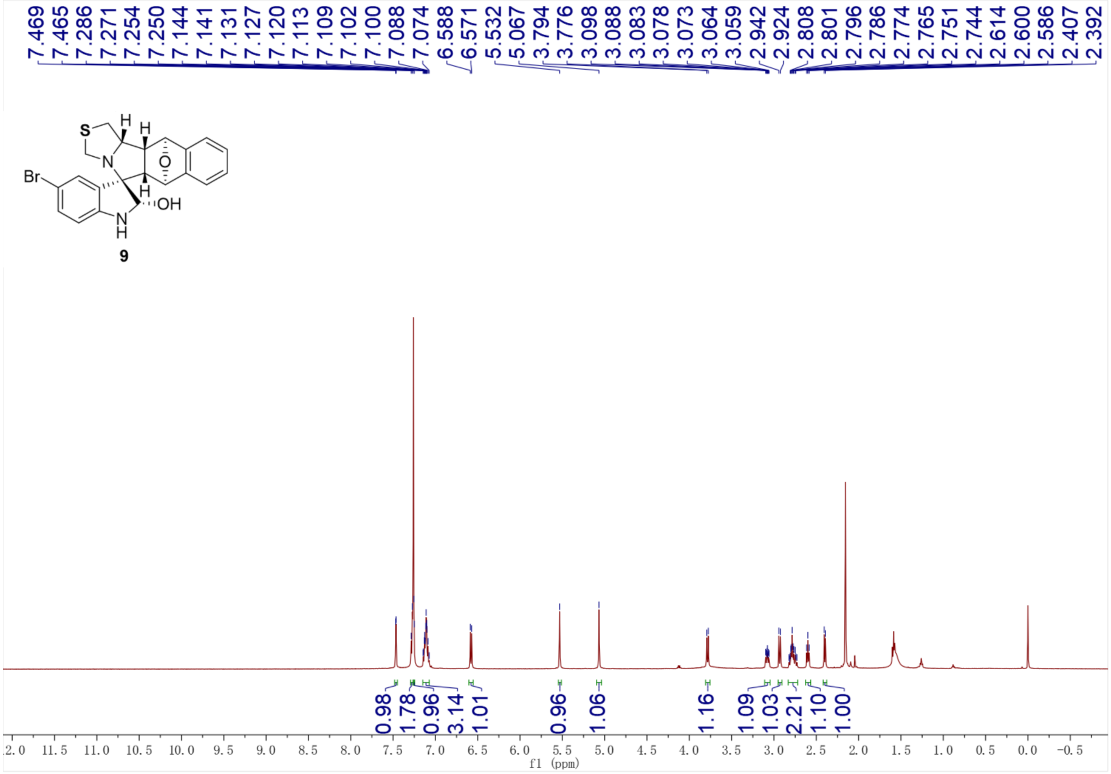


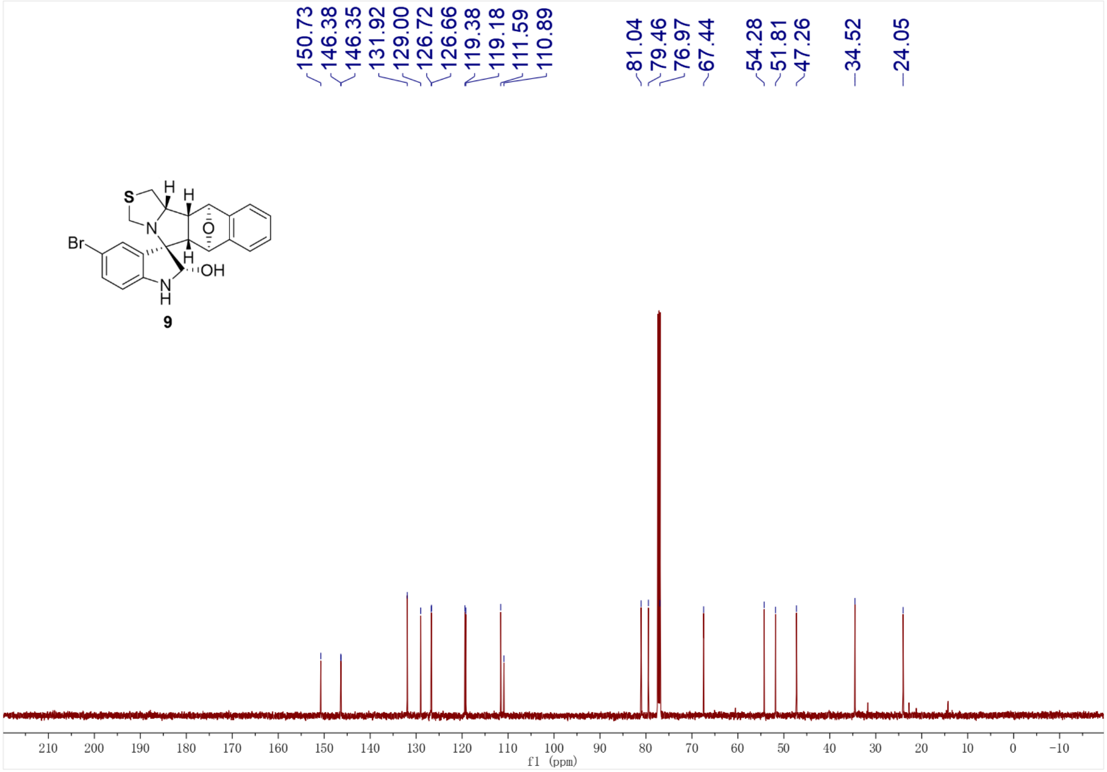


**$^{1}$H NMR** (500 MHz, $CDCl_3$), **$^{13}$C NMR** (125 MHz, $CDCl_3$) spectrum of **9**
